# The Abl1 tyrosine kinase is a key player in doxorubicin-induced cardiomyopathy and its p53/p73 cell death mediated signaling differs in atrial and ventricular cardiomyocytes

**DOI:** 10.1186/s12967-024-05623-8

**Published:** 2024-09-16

**Authors:** Jürgen Borlak, Yari Ciribilli, Alessandra Bisio, Saravanakumar Selvaraj, Alberto Inga, Jung-Hwa Oh, Reinhard Spanel

**Affiliations:** 1 Centre for Pharmacology and Toxicology, Hannover Medical School, Carl-Neuberg-Str. 1, 30625 Hannover, Germany; 2 Department of Cellular, Computational and Integrative Biology (CIBIO), University of Trento, Trento, Italy; 3 Department of Predictive Toxicology, Korea Institute of Toxicology, Daejeon, Republic of Korea

**Keywords:** Toxic cardiomyopathy, Heart failure, Animal models of human disease, Abl1/p53/p73 cell death signaling

## Abstract

**Background:**

Doxorubicin is an important anticancer drug, however, elicits dose-dependently cardiomyopathy. Given its mode of action, i.e. topoisomerase inhibition and DNA damage, we investigated genetic events associated with cardiomyopathy and searched for mechanism-based possibilities to alleviate cardiotoxicity. We treated rats at clinically relevant doses of doxorubicin. Histopathology and transmission electron microscopy (TEM) defined cardiac lesions, and transcriptomics unveiled cardiomyopathy-associated gene regulations. Genomic-footprints revealed critical components of Abl1-p53-signaling, and EMSA-assays evidenced Abl1 DNA-binding activity. Gene reporter assays confirmed Abl1 activity on p53-targets while immunohistochemistry/immunofluorescence microscopy demonstrated Abl1, p53&p73 signaling.

**Results:**

Doxorubicin treatment caused dose-dependently toxic cardiomyopathy, and TEM evidenced damaged mitochondria and myofibrillar disarray. Surviving cardiomyocytes repressed Parkin-1 and Bnip3-mediated mitophagy, stimulated dynamin-1-like dependent mitochondrial fission and induced anti-apoptotic Bag1 signaling. Thus, we observed induced mitochondrial biogenesis. Transcriptomics discovered heterogeneity in cellular responses with minimal overlap between treatments, and the data are highly suggestive for distinct cardiomyocyte (sub)populations which differed in their resilience and reparative capacity. Genome-wide footprints revealed Abl1 and p53 enriched binding sites in doxorubicin-regulated genes, and we confirmed Abl1 DNA-binding activity in EMSA-assays. Extraordinarily, Abl1 signaling differed in the heart with highly significant regulations of Abl1, p53 and p73 in atrial cardiomyocytes. Conversely, in ventricular cardiomyocytes, Abl1 solely-modulated p53-signaling that was BAX transcription-independent. Gene reporter assays established Abl1 cofactor activity for the p53-reporter PG13-luc, and ectopic Abl1 expression stimulated p53-mediated apoptosis.

**Conclusions:**

The tyrosine kinase Abl1 is of critical importance in doxorubicin induced cardiomyopathy, and we propose its inhibition as means to diminish risk of cardiotoxicity.

**Supplementary Information:**

The online version contains supplementary material available at 10.1186/s12967-024-05623-8.

## Background

The emergence of cardiooncology with its focus on cardiotoxicity is a hot topic [1], and in a state-of-the-art review, Sheng and colleagues summarized the cardiovascular complications arising from anticancer therapies [2]. Many anticancer drugs but also radiation therapies cause myocardial injury. Unexpectedly, and despite high specificity, molecular targeted therapies also induce a wide spectrum of cardiovascular complications. Prominent examples include antibody-based therapies targeting HER2, VEGF and the checkpoint inhibitors PD1/PD-L1 in addition to tyrosine kinase and mTOR inhibitors. Similarly, immunomodulators, alkylating agents, taxanes and antimetabolites received drug labels for their potential harmful effects on the myocardium [2].

The anthracycline doxorubicin is highly effective in hematological as well as solid malignancies. However, its dose-dependent induction of cardiotoxicity limits its use. Doxorubicin blocks RNA and DNA synthesis equally, and cells in the S-phase of the cell cycle are most sensitive to the drug. The drug causes cell death by two main mechanisms: (A) Intercalation, i.e. doxorubicin intercalates between opposite nucleotides along the DNA and forms tight DNA-drug bonds, which disrupt DNA synthesis and gene transcription. (B) Enzyme inhibition, i.e. doxorubicin binds to and inhibits topoisomerase II, i.e. a key enzyme in strand unwinding of supercoiled DNA, which is a critical step for gene transcription and DNA replication. Moreover, metabolism of the drug generates free radicals, which damage DNA and triggers programmed cell death.

As recently summarized, several molecular events run in parallel in doxorubicin-induced cardiotoxicity [3]; nonetheless, the production of free radicals is considered to be a key event. Oxidative stress induces mitochondrial permeability transitions and alterations in mitochondrial calcium transport. Given that a single cardiomyocyte contains about ∼ 7000 mitochondria [4], it is of no surprise that drugs causing mitochondrial toxicity are prone to elicit cardiotoxicity and eventually impair cardiac contraction [5]. However, the notion that doxorubicin causes cardiotoxicity primarily via the production of reactive oxygen species (ROS) has been challenged, partly because ROS detoxifying agents failed to mitigate cardiotoxicity in animal models [6]. Furthermore, oxidative stress may damage the plasmalemma. Indeed, a complex relationship exists between ROS and inflammation and involves various immune cells of the myocardium, especially macrophages, monocytes, dendritic cells and lymphocytes [7] in addition to interferon gamma signaling [8]. Following doxorubicin treatment the morphological changes of cardiomyocytes include reduced caliber size and density of the myofibrillar bundles [9], alterations in the Z-disc structure, disarray and depolymerization of actin filaments. Moreover, doxorubicin stimulates cell death programs [10], alters iron homeostasis [11] and activates p53, i.e. a key transcriptional regulator of programmed cell death [12, 13]. Note that a recent study reported allosteric inhibition of BAX to protect mice from doxorubicin-induced cardiomyopathy [14].

Although pegylated liposomal drug formulations have a better cardiac safety profile, the paradox of why patients on doxorubicin single or combination therapy develop cardiac complications, even after years of treatment cessation and remission from disease, remains unresolved. In fact, an important contraindication for this drug is pre-existing heart disease, and to diminish the risk of cardiotoxicity, the lifetime cumulative dose should not exceed 550 mg/m² [15, 16]. Next to acute toxicity, an understanding of delayed cardiotoxicity is required, and we need to identify patients at high risk of developing cardiomyopathy and congestive heart failure. Together, there is unmet need for an improved understanding of the causes of doxorubicin-induced cardiomyopathy and to develop strategies to circumvent dose-limiting side effects.

To gain insight into doxorubicin-induced cardiomyopathy, we conducted a functional genetic and genomic study on histologically well-defined cardiac lesions. We were particularly interested in determining the genetic events in the onset of cardiotoxicity after treatment of rats with doxorubicin, and we searched for possible gene targets for attenuation of cardiotoxicity. The animal model is highly sensitive to doxorubicin treatment, enables mechanistic studies and recapitulates the disease pattern seen in patients at clinically relevant doses. Furthermore, an analysis of gene promoters of disease-regulated genes allowed us to determine molecular rules for their transcriptional regulation and to obtain clues for mechanisms underlying cardiotoxicity.

We observed considerable heterogeneity in cardiomyocyte responses to doxorubicin treatment, and the data are highly suggestive for cardiomyocyte subpopulations while genomic-footprints identified Abl1 and p53 binding sites in promoters of regulated genes. Owing to its functions in cardiac growth and development [17], we investigated Abl1’s role in doxorubicin-induced cardiomyopathy, and through gene reporter assays established Abl1’s cofactor activity on p53 target genes. It is well known that Abl1 activates p73 in response to DNA damage [18–20]. However, there are only a few reports suggesting Abl1 to directly stimulate p53-dependent cell death programs [21–23]. One study showed Abl1 to bind in-vitro to the carboxyl-terminal regulatory domain of p53 and to prevent its dissociation with the DNA complex [24]. Another study reported a protective role of Abl1 in neutralizing the inhibitory effects of Mdm2 on p53 [25]. In fact, Abl1 phosphorylates MDM2 and its posttranslational modification impairs binding of MDM2 to p53. As a result, p53 is protected from MDM2-dependent degradation, and this defines an Abl1-Mdm2-p53 signaling axis. Moreover, Abl1 may stimulate p53 activity indirectly through p38-p53-Mdm2 signaling as shown in cancer cells [26].

Together, our study highlights a critical role of Abl1 in cardiac cell death which differed between atrial and ventricular cardiomyocytes. Furthermore, we observed heterogeneity of cellular responses, and based on transcriptomics, obtained evidence for cardiomyocyte subpopulations. We propose a strategy to alleviate doxorubicin induced cardiomyopathy by blocking Abl1 activity. Additionally, we gained deep insight into the genomic responses of cardiomyocytes as they relate to mitochondrial toxicity, programmed cell death, inflammation and toxic cardiomyopathy that is orchestrated, at least in part, by Abl1 transcriptional cofactor activity.

## Materials and methods

### Animals and tissue

We strictly followed the Public Health Service (PHS) Policy on Humane Care and the Use of Laboratory Animals of the National Institutes of Health, USA and obtained approval to perform experiments from the animal welfare ethics committee of the State of Lower Saxony, Germany (‘Lower Saxony State Office for Consumer Protection and Food Safety’, LAVES). The approval ID is Az: 33.9-42502-04-06/1081.

We divided 30 Sprague-Dawley (SD) rats (Charles River, Sulzfeld, Germany) into 5 groups, consisting of 6 animals each. We treated 24 rats with varying doses of doxorubicin dissolved in 0.9% NaCl. The controls (*N* = 6) received an i.p. injection of physiological saline.

The treatment groups comprise the following study arms: T1 (1 mg/kg, daily i.p. for 3 days, *N* = 6), T2 (10 mg/kg, daily i.p. for 3 days, *N* = 6), T3 (20 mg/kg, daily i.p. for 3 days, *N* = 6) and T4 (20 mg/kg, daily i.p. for 6 days, *N* = 6). Such treatments translate to human doses of 6, 60 and 120 mg/m^2^ body surface area ([27–29], https://www.fda.gov/media/72309/download). Except for the low-dose (T1), all doses are clinically relevant which typically range between 50 and 80 mg/m2 (monotherapy) and 30–60 mg/m2 (combination chemotherapy). Correspondingly, the cumulative doses are 18, 180, 360 and 720 mg/m2 body surface area. Additionally, we examined the ultrastructure of ventricular tissue by transmission electron microscopy following daily i.p. dosing of rats (*N* = 5) at 5 mg/kg i.p. for five consecutive days.

### Organ harvest

We anesthetized rats by intraperitoneal injection of ketamine (10 mg/100 g body weight) and rompun (0.5 mg/100 g body weight) followed by an i.p. injection of 200 I.E. heparin. Upon complete anesthesia, we performed an abdominal and thoracic incision and harvested the organs carefully. Prior to processing for histopathology, we examined the heart for suspicious macroscopic abnormalities and took biopsies from the ventricles for the scheduled genomic studies. The biopsies were immediately frozen in liquid nitrogen.

### Histopathology

We recorded the whole-body weight and the weights of large parenchymal organs and stored the organs in buffered 4% formaldehyde. For each treatment group, we cross-sectioned formalin fixed hearts from the tip to the base and prepared five lamellae for *N* = 3 animals. For the remaining 3 animals, we prepared three lamellae following longitudinal and frontal sectioning of the heart. We used standard operating procedures for the paraffine-embedding and stained 3-micrometer thick sections with H&E to assess cardiac toxicity following doxorubicin treatment. Furthermore, we visualized myofibrils and cardiac muscle striations by employing the Crossmon and the Haidenhain’s iron hematoxylin stain and used the Luxol fast blue stain to investigate doxorubicin induced myofibrillar degeneration and contraction bands [30].

### Immunohistochemistry

1–2 μm thick heart sections were deparaffinized and rehydrated in a descending alcohol series followed by a washing step in distilled H_2_O. To achieve antigen retrieval the sections were incubated in a Tris-EDTA buffer (pH 9.0) in a water bath at 98 °C for 30 min. We used the ZytoChem-Plus HRP Polymer-Kit of Zytomed Systems, Germany for IHC staining. The slides were rinsed with distilled H_2_O and after a 5 min incubation step in tris-buffered saline (washing buffer), endogenous peroxidase activity was blocked with 3% peroxidase blocking reagent (Merck, Germany) for 5 min followed by a second washing step. Thereafter, we applied the protein-block serum free reagent and allowed to react for 5 min (ZytoChem-Plus HRP Polymer-Kit, reagent 1) followed by an incubation step with primary antibodies for 60 min.

**Table:** 

Antibody	Vendor	Cat no.	Lot number	Dilution	Antigen retrieval	Ab-titer
Abl1	abcam	ab15130	GR3201356-2	1:50	ph9 (30 min)	1:150
p53	Santa Cruz	sc-98	#J1116	1:25	ph9 (1,5 h)	1:150

We incubated the bound primary or bridging antibody with labeled polymer HRP anti-rabbit secondary antibody (ZytoChem-Plus HRP Polymer-Kit, reagent 2) for 20 min and added reagent 3 of the ZytoChem-Plus HRP Polymer-Kit to finally place the slides in a moist chamber at room temperature allowing an incubation time of 30 min. After completion of the HRP reaction, we counterstained the sections with Hematoxylin for 5 min, washed the slides under running warm tap water for 10 min and dehydrated the sections in a cabinet at 60 °C for 20 min. The sections were coverslipped and examined under a light microscope (Nikon Ni-E microscope, Japan), and we captured images with the Nikon NIS basic research microscopic imaging software version 4.3.

### TEM analysis of cardiomyocytes and mitochondria

Using standard protocols, we examined the ultrastructure of cardiomyocytes and mitochondria of control and doxorubicin-treated animals by transmission electron microscopy (TEM). Initially, we prepared the left ventricle of the heart and fixed the tissue with 2.5% glutaraldehyde in 0.1 M phosphate (pH 7.3) at room temperature for 2 h followed by 1% OsO4 plus 1.5% potassium ferrocyanide in 0.1 M phosphate buffer (pH 7.3) at 4 °C in the dark for 1 h. The dehydration step consisted of an ascending ethanol and propylene oxide series, and we placed the tissue in the EMbed 812 resin. We performed the infiltration step and the polymerization of the resin according to SOPs and prepared ultrathin sections (70 nm) with an ultramicrotome (UltraCut-UCT, Leica, Austria). We applied the sections onto 150 mesh copper grids. After staining with 2% uranyl acetate (10 min) and lead citrate (5 min), we examined the sections by transmission electron microscopy at 120 kV (Technai G2 Spirit Twin, FEI, Hillsboro, USA).

### Genomic experiments

We extracted RNA from heart tissue biopsies of doxorubicin and saline (= control) treated animals with the RNeasy kit (Qiagen, Hilden, Germany) and performed whole genome scans with the Agilent Rat Oligo Microarray G4130A (Agilent Technologies, Palo Alto, USA) according to the manufacturer’s recommendations. This platform consists of 60-mer oligonucleotides with sequences representing over 20,000 well-characterized rat transcripts. We hybridized duplicate microarrays for each experiment and labeled the treatment and control samples with the Cy3 and Cy5 fluorophores (color swap). Further details on the experimental procedures are given in our previous studies [31–33].

### Analysis of genomic data

We retrieved gene expression data from scanned images using the Feature Extraction Software (Agilent Technologies). For each experiment, we combined the gene expression data of duplicate microarray experiments using the software Rosetta Luminator according to the manufacturer’s recommendation (Rosetta Inpharmatics LLC, Seattle, WA).

We employed analysis of variance (ANOVA) to determine statistical significance of regulated genes. We calculated fold-changes based on the intensity values of controls (vehicle-treated rats) and of doxorubicin-treated animals. Additionally, we interrogated promoter sequence of genes regulated > 2-fold as detailed below. We classified doxorubicin-regulated genes systematically according to their roles in cellular biology by considering the Gene Ontology (GO) terms and by querying the AmiGO browser (http://www.godatabase.org/cgi-bin/amigo/go.cgi) and GeneCards (http://bioinfo.weizmann.ac.il/cards/ index.shtml) database. If a rat gene was not assigned to a specific biological process, we used the information for the human ortholog (http://www.ncbi.nlm.nih.gov/entrez/query.fcgi? db = homologene).

In order to identify cardiotoxicity co-regulated genes, we performed hierarchical gene cluster analysis. To log-transformed data, we applied the average linkage method using the uncentered correlation (a variant of Pearson correlation) as implemented in the software cluster. The results were visualized with the software TreeView (http://rana.lbl.gov/EisenSoftware.htm).

### Gene enrichment analysis

We used the software Metascape [34] and GeneXplain (https://genexplain.com) to identify enriched Gene Ontology (GO) terms. Only terms with an FDR-adjusted p-value < 0.05 were considered.

### Sequence retrieval

We used the UCSC Genome Browser (www.genome.ucsc.edu/) to extract the 5´-upstream sequences of the transcription start sites (TSS) of doxorubicin-regulated RefSeq-annotated genes and the 5´-upstream sequences of the non-regulated RefSeq-annotated control genes. We extracted 2900 bp upstream and 100 bp downstream of TSS, respectively.

### Promoter analysis

We only considered gene promoter sequence of RefSeq annotated genes. We found 15 genes to be regulated > 4-fold and 85 genes regulated between < 4-fold and > 2-fold. From the same experimental data, we selected 97 non-regulated genes as controls. In order to confirm the relevance of the extracted 5´-upstream sequences, we applied the tool Dragon Promoter Finder™. The algorithm recognizes functional TSSs in RNA polymerase II promoter regions of vertebrates, and the tool is included in the software package TRANSPLORER^®^ (BIOBASE, Wolfenbuettel, Germany; http://www.biobase.de/). The most widely used method for recognition of transcription factor (TF) binding sites is the application of positional weight matrices (PWMs) [35]. To independently confirm transcription factor binding sites, we used the GENOMATIX tool MatInspector™ (http://www.genomatix.de/) and the MATCH suite which is integrated in the geneXplain platform. Both tools use the most up-to-date library of PWMs derived from the TRANSFAC^®^ databases. The cut-off scores for the PWM matrices were set to a very low value to minimize false-negative predictions. Moreover, to confirm predicted binding sites, we employed electromobility band shift assays and therefore interrogated cognate binding sites of gene specific promoters. Additionally, we developed gene reporter assays to evaluate the functional importance of transcription factor binding sites, as detailed below.

### Positional weight matrix (PWM) for Abl1 and p53

We constructed a PWM for Abl1 on the basis of 44 published experimentally proven DNA binding sequences [36]. Subsequently, we employed a matrix generation tool which is integrated into the BIOBASE tool MATCH™. Supplementary Table S1 shows the PWMs which we used to interrogate promoter sequences for Abl1 and p53 consensus binding sites of doxorubicin regulated genes.

### Identification of composite regulatory modules (CM)

A composite module (CM) is a set of TF weight matrices with defined matrix cut-offs and other parameters associated with a specific nucleotide sequence of a regulatory regions. The TRANSPLORER^®^-integrated CMFinder tool determines CMs in a set of promoters (or other regulatory) sequences of regulated genes.

“Profile 1” was created by comparing sequences up- and downstream of putative Abl1 binding sites (+/- 100 bp) of doxorubicin-regulated genes (“positive” data set, 18 genes) with binding sites of non-regulated genes (“false-positive” data set, 25 genes).

“Profile 2” was created by comparing sequences up- and downstream of putative Abl1 binding sites (+/- 100 bp) of doxorubicin-regulated genes (“positive” data set) with sequences (about 225 bp) of non-regulated genes with no binding site (“negative” data set, 31 genes).

### Confirmation of p53 binding sites in doxorubicin regulated genes

In 2019 Fischer performed a meta-analysis of published p53 gene regulatory networks in mice and humans [37]. This repository compiles independent data sets of p53-dependent gene regulations across a wide range of cell lines and included 9 chromatin immunoprecipitation (ChIP) studies in mice with experimental proof for a gene to be bound by p53 within 5 kb of their transcriptional start site (TSS). We used this information to independently confirm p53 target genes in our cardiac genomic data.

### Electrophoretic mobility shift assay (EMSA)

We used established protocols [31, 32] to isolate nuclear proteins from rat heart and liver tissue of doxorubicin-treated animals. Additionally, we studied Abl1 expression and DNA binding activity in cultures of rat hepatocytes following doxorubicin treatment at 1 µg/ml for 12 h, 24 h, 48 h, 72 h and 96 h. The chosen drug concentration is well within Cpmax (= peak plasma concentration) of patients following intravenous applications of the drug [38].

The oligonucleotide probes harbour Abl1 consensus binding sites in gene specific promoters of doxorubicin-regulated genes, and the sequences are listed in supplementary Table S2. Two oligonucleotides served as positive controls, and the binding of Abl1 to DNA was independently proven [39]. As negative controls, we used two oligonucleotides with the *in silico*-identified Abl1 binding sites from rat gene promoters not regulated by doxorubicin. Besides, we designed 4 oligonucleotides where the binding sites were mutated to abolish Abl1 binding.

We performed EMSA as previously described [40, 41]. In brief, 5 µg of nuclear extract were incubated on ice for 20 min. The binding buffer consisted of 25 mM HEPES (pH 7.6), 5 mM MgCl_2_, 34 mM KCl, 2 mM dithiothreitol (DTT), 2 mM Pefablock (Boehringer Mannheim, Ingelheim am Rhein, Germany), 0.5 µl aprotinin (2.2 mg/ml, Sigma-Aldrich, Deisenhofen, Germany), 50 ng poly (dl-dC) and 80 ng Bovine Serum Albumin (BSA, PAA, Linz, Austria). To demonstrate specificity, we added a 100-fold excess of unlabeled oligonucleotides to the reaction mixture in competition assays. For EMSA super-shift assays, we employed two different antibodies directed against Abl1, i.e. A5844 (Sigma-Aldrich Chemie, Munich, Germany) and sc-131 (Santa Cruz Biotechnology, Heidelberg, Germany) and allowed the antibodies to react with nuclear extracts at 4 °C for 20 min. Gels were blotted to Whatman 3 MM paper, dried under vacuum, exposed to imaging screens (Imaging Screen-K, Bio-Rad, Munich, Germany) at room temperature for 16 h and 72 h, and analyzed with a phosphor imaging system (Molecular Imager FX pro plus; Bio-Rad) and the Quantity One software (Bio-Rad).

### Cell lines and culture culture

We purchased the MCF7 breast cancer and the P19Cl6 mouse cell line, i.e. a clonal derivative isolated from murine P19 embryonic carcinoma cells, from the Interlab Cell Line Collection (ICLC, Genoa, Italy). The P19Cl6 cell line differentiates into beating cardiomyocytes when cultured in 1% dimethyl sulfoxide [42] and therefore served as one of the cardiomyocyte models. The cells were cultured in DMEM medium supplemented with 10% Fetal Bovine Serum (FBS), 2 mM L-glutamine and antibiotics, and the P19Cl6 cells were cultured along with 1% DMSO for 6–9 days to stimulate cardiomyocyte-like differentiation (all reagents were purchased from Gibco, Life Technologies, Milan, Italy). MCF7 cells stably silenced for p53 (MCF7 shp53) and the control (MCF7 empty vector) was cultured in RPMI medium (Gibco) supplemented as described above but with the addition of 1 µg/ml puromycin (Life Technologies) to maintain the selection.

We also obtained the human induced pluripotent stem (iPS) cell-derived cardiomyocytes, iCell^®^ cardiomyocytes, from Cellular Dynamics International (CDI, Madison, USA). The cells were thawed and cultured in iCell cardiomyocytes plating and maintainance media in gelatin-coated containers, according to the manufacturer’s instructions.

### RT-qPCR experiments in human iCell cardiomyocytes, MCF7 and P19Cl6 cell lines

We processed the MCF7 and cardiomyocyte-like differentiated cells as described above. Sixteen hours after treatment, we harvested the cells, transferred them into 1.5 ml tubes and washed them once with ice-cold PBS. We extracted total RNA with the RNeasy Kit (Qiagen) according to the manufacturer’s instructions. The human iCell^®^ cardiomyocytes were seeded onto 96-well plates at a 70–80% confluence. Twenty-four hours after seeding, we treated the cell cultures with doxorubicin for 16 h (1.5 µM or 0.1 µM, respectively for MCF7 and differentiated P19Cl6 cells or cardiomyocytes), lysed them by adding the TRIzol reagent (Life Technologies), and extracted RNA according to the manufacturer’s recommendation. Typically, we generated cDNA from 1 µg to 0.5 µg (human iCell^®^ cardiomyocytes) of RNA by following the manufacturer’s protocol of the RevertAid M-MuLV First Strand cDNA Synthesis Kit (Fermentas, ThermoFisher Scientific, Milan, Italy). We run real-time qPCR on RotorGene 6000 (Corbett Life Science, Ancona, Italy) or CFX384 (Bio-Rad, Munich, Germany) qPCR systems by adding the Kapa SYBR green-based master mix (KapaBiosystem, Resnova, Rome, Italy) to the reaction mixture. The sequences of primers are given in supplementary Table S3, and transcript quantification is based on the ΔΔCT method with glyceraldehyde 3-phosphate dehydrogenase (GAPDH) and the β_2_microglobulin (B2M) as housekeeping/normalizer genes [43].

### Cloning of human Abl1 into inducible expression vectors for yeast

We utilized a PCR-based approach to clone the human full-length Abl1 cDNA into the pLSG and pLSG-TAD (= topologically associating domain) plasmid expression vectors [44]. The vectors originated from the plasmid pRS315 and contained centromer and origin of replication for stable propagation at low copy number in yeast cells, the LEU2 selection marker and the *GAL1*,*10* promoter enabled inducible expression of cloned cDNAs [45]. The plasmid pLSG-TAD also contained an acidic transactivation domain, derived from the human p53 gene and was cloned downstream of the *GAL1*,*10* promoter. Both vectors contained a terminator sequence, derived from the *CYC1* gene and an intervening multi-cloning site.

To obtain genomic Abl1, we extracted total RNA from the human MCF7 breast cancer and the HCT116 colon cancer cell line using standard protocols (RNeasy mini, Qiagen). We initiated cDNA synthesis from 1 µg of RNA with the First Strand DNA synthesis kit and random decamers (ABgene, Resnova, Milan, Italy). To facilitate the amplification reaction, the 3,394 nt Abl1 cDNA was amplified as two separate fragments (N-ter and C-ter) with an overlap of ∼ 700 nt. The forward primer for the N-ter amplicon and the reverse primer for the C-ter amplicon contained 45 nt homology tails for the pLSG-TAD plasmid (TAD and CYC1 terminator sequences, respectively). The homology between the PCR products and the plasmid enabled direct cloning in yeast using a “gap repair” approach that exploits the high proficiency of *S. cerevisiae* for homologous recombination [46]. We co-transformed the Abl1 templates in yeast with pLSG-TAD linearized at the multi-cloning site and selected transformants on plates lacking leucine. By DNA sequencing, we verified the correct integration of both PCR fragments and reconstitution of full-length wild type Abl1 cDNA in-frame with the TAD (pLSG-TAD-Abl1) in yeast transformant clones. To remove the acidic transactivation domain from the pLSG-TAD-Abl1 plasmid, we utilized another gap repair strategy. The vector was linearized using a restriction site between the *GAL1*,*10* promoter and the TAD domain and transformed in yeast together with a PCR product of the Abl1 N-ter region that was generated with a forward primer and a homology tail for the *GAL1*,*10* promoter. Through homologous recombination, this PCR product replaced the region of the vector containing the TAD domain. Finally, plasmid recovery, restriction pattern analysis and DNA sequencing confirmed the correct construction of the pLSG-Abl1 vector. See supplementary Table S4A for a list of primers used for the cloning and sequencing experiments.

### Construction of Abl1 yeast reporter strains

Starting from the luciferase-based reporter strain yLFM-ICORE, we prepared a panel of isogenic Abl1 yeast reporter strains [45]. Putative Abl1 binding sequences were chosen based on a published Position Weight Matrix (PWM) [39], and we constructed a perfect consensus (CONS: AAAAAACAACAA) binding site consisting of the most frequent bases at each position in the PWM, and a weak consensus (DEG: CACAACAAAGAG) consisting of the second most frequent bases at each position of the PWM. By following the delitto-perfetto in vivo mutagenesis protocol [47] two copies of CONS and DEG sequences were introduced upstream of the minimal CYC1 promoter which controls the luciferase reporter in the yLFM-ICORE strain. We also developed a reporter strain that contained the CONS sequence adjacent to the p53 response element found in the promoter of the human *PUMA* gene (*BBC3*) (CONS-PUMA). We present the sequence of the primers for reporter strain development in supplementary Table S4B.

### Luciferase assays in yeast one-hybrid (Y1H) assay

We transformed the yeast Abl1 reporter strains with the pLSG-TAD-Abl1 and pLSG-Abl1 and, when needed, with the human p53 expression plasmid [47] by the LiAc-based protocol. We performed Luciferase assays upon induction of Abl1 expression at moderate (0.016%) and high (2%) galactose levels. We obtained soluble protein extracts of lysed cell cultures and quantified the extracts with the BCA assay (Pierce Biotechnology, Thermo Fisher Scientific; Waltham, USA). We measured the activity of the luciferase reporter using the BrightGlo assay reagent (Promega, Milan, Italy) on a multilable Mithras LB-940 plate reader (Berthold technologies, Milan, Italy).

### Western blotting and immunofluorescence microscopy

We examined CDK1 (BM2206P, Acris Antibodies GmbH, Hiddenhausen, Germany which has been acquired by OriGene Technologies) and Abl1 protein expression (A5844, Sigma-Aldrich Chemie, Munich, Germany) in nuclear extracts of heart and liver tissue and cell culture assays by Western immunoblotting. In addition, we investigated Abl1 intracellular localization by immunofluorescence microscopy. In the yeast one-hybrid (Y1H) assay, we observed induced Abl1 expression in cell cultures containing galactose for 6 h. Specifically, for the Western blots, soluble proteins were prepared from pLSG-TAD-Abl1 yeast transformants by mechanical cell disruption with 0.5 mm acid-washed glass beads (Sigma-Aldrich) in lysis buffer (100 mM NaCl, 20 mM Tris-HCl pH 7.2, 5 mM EDTA, 2 mM DTT, 0.1% NP40, 10% Glycerol, 2% SDS, 100 µg/ml PMSF, 1 µg/ml Leupeptin, 2 µg/ml Aprotinin, 1 µg/ml Pepstatin A). After washing in NET buffer (100 mM NaCl, 50 mM Tris-HCl pH 8, 5 mM EDTA) the protein extracts were quantified (BCA assay, Pierce Biotechnology, ThermoFisher Scientific) and loaded onto 7.5% acrylamide gels. We performed SDS-PAGE at the constant voltage of 180 V, followed by protein transfer onto PVDF membrane (Amersham, GE Healthcare, Chicago, USA) according the manufacturer’s recommendations (Bio-Rad). We used the sc-126 primary antibody (Santa Cruz Biotechnology) for immuno-detection of the TAD domain and visualized the protein with the ECL plus kit (Amersham).

Immunofluorescence microscopy: We fixed log-phase growing galactose cultures of Abl1 transformants in 4.5% formaldehyde (Sigma-Aldrich) for 1 h followed by a centrifugation step at 3,000 rpm for 5 min. The pellets were washed twice, re-suspended in phosphate buffer (1 M sorbitol, 0.1 M KH_2_PO_4_, pH 7.5) and incubated at 37 °C with lyticase (20,000 U/ml stock solution, Sigma-Aldrich) in the presence of 2β-mercaptoethanol (Sigma-Aldrich) for 40 min. Spheroplasts were allowed to settle onto SuperFrost plus-coated microscope slides (Menzel Glaser, Braunschweig, Germany), and we carefully eliminated excess solution. Subsequently, we fixed the cells in pre-chilled methanol for 6 min and acetone for 30 s. The slides were washed three times with PBS-1%BSA and incubated with the sc-126 primary antibody at a 1:100 dilution. Immuno-complexes were detected by adding a FITC-conjugated anti-mouse secondary antibody (1:300 dilution in PBS-1% BSA, Sigma-Aldrich), and the nuclei were counterstained with DAPI (1:250 dilution, Life Technologies). The slides were mounted with Moviol and visualized on a fluorescence microscope (Zeiss, Arese (MI), Italy).

#### p53 gene reporter assays in human cells

We used the pCI-Neo vector backbone and cloned full-length human Abl1 cDNA into the T7 and T3 RNA polymerase promoter region between the XhoI and SalI restriction sites. We confirmed the correct insertion of Abl1 cDNA by restriction pattern analyses and DNA sequencing. We used the pC53-SN3 p53 expression vector (gift from Dr. B. Vogelstein, Johns-Hopkins-Universität, Baltimore, USA) for ectopic expression of p53. The reporter plasmid PG13 (gift from Dr. B. Vogelstein) contains 13 copies of a p53 response element upstream of the luciferase reporter gene [48]. We used the plasmid pRL-SV40 (Promega) to normalize transfection efficiency. Specifically, we seeded MCF7 cells in 12-well plates, and 250 ng of reporter plasmid, 100 ng of p53 plasmid and 400 ng of Abl1 expression plasmid were transfected with the Fugene6™ (Roche, Basel, Switzerland) reagent as previously reported [49]. To keep the DNA amount transfected constant, we used the pCMV Neo-Bam empty vector in controls without the p53 or Abl1 plasmids. Thirty-two hours post-transfection, we initiated doxorubicin treatment and assayed luciferase activity after additional 16 h with the Dual Luciferase Assay system (Promega) according to the manufacturer’s protocol. We measured the luciferase activity on an Infinite M200 multiplate reader (Tecan, Milan, Italy).

### Apoptosis assay in MCF7 cells

We seeded MCF7shp53 or empty vector control cells onto 60 mm plates (Corning, Merck/Sigma-Aldrich) and allowed the cultures to reach 70% confluence prior to the transfection. Twenty-four hours later, the cells were transiently transfected with either TransIT-LT1 (Mirus Bio, Tema Ricerca, Bologna, Italy) or Lipofectamine 3000 (Life Technologies) using 1, 2 and 5 µg of the empty (pCI-neo) or the Abl1 expression vector according to manufacturer’s recommendations. Eight hours post-transfection, the cells were treated with 1.5 µM doxorubicin for 48 h and analyzed for apoptosis induction by flow cytometry (Annexin V staining) and Western blotting of PARP (= poly ADP-ribose polymerase). For the flow cytometry assay, we resuspended 1 × 10^6^ cells/ml in 1X Annexin V Binding Buffer (0.01 M Hepes/NaOH pH 7.4, 0.14 M NaCl, 2.5 mM CaCl_2_) and transferred 100 µl of the solution into a 5 ml polystyrene FACS tube (BD Biosciences, Franklin Lakes, USA). The samples were stained with the FITC-Annexin V antibody (BD Biosciences) and TO-PRO-3 Iodide (ThermoFisher Scientific) dye to allow the identification of live/necrotic/apoptotic cells. The flow cytometry assays were performed on a FACS Canto A station (BD Biosciences) as previously described [50].

To determine PARP cleavage fragments, we lysed the cells in RIPA buffer (150 mM sodium chloride, 1% NP-40, 0.5% sodium deoxycholate, 0.1% SDS, 50 mM Tris, pH 8.0) supplemented with 1X Protease Inhibitors (PIs) (Roche). We loaded 30–50 µg protein extracts onto 10% acrylamide (Bio-Rad) gels and analyzed PARP fragments by Western blotting. Nitrocellulose membranes (Amersham) were blocked with 5% non-fat milk dissolved in a solution of PBS-0.1% Tween20 (PBS-T). We obtained antibodies for Abl1 (clone 8E9, sc-56887) and GAPDH (clone 6C5, sc-32233) from Santa Cruz Biotechnology, and PARP from Cell Signaling Technology (Euroclone, Milan, Italy; #9542). To confirm p53 activation in response to doxorubicin treatment, we assayed p53 (clone DO-1, sc-126) and the p53 targets p21 (clone C-19, sc-397) and MDM2 (SMP14, sc-965) by Western blotting. All antibodies were purchased from Santa Cruz Biotechnology. We diluted primary as well as HRP-conjugated secondary antibodies (Sigma-Aldrich) in 1% non-fat milk in PBS-T. We detected immunoreactive bands with an ECL Select (Amersham) reagent and Alliance LD2 (UVITec, Cambridge, UK) documentation system and quantified the blots with the Image J64 software.

#### Statistical testing

We used the Shapiro-Wilk test to examine normal distribution and performed a t-test to determine statistical significance. We performed power calculations based on the balanced one-way analysis of variance by considering the heart weight of control and doxorubicin treated animals and used the following criteria: Group size k = 3 (control, 10 & 20 mg/kg treatment group), effect size f = 1.052626, power = 0.8 and significance level = 0.05. This defined a minimum of *N* = 4 animals per treatment group. Notwithstanding we used *N* = 6 animals per group. Except for the power analysis which was computed in R, all statistics were tested with the software GraphPad Prism 8.

## Results

### Doxorubicin-induced multi-organ toxicity

**Fig. 1:**
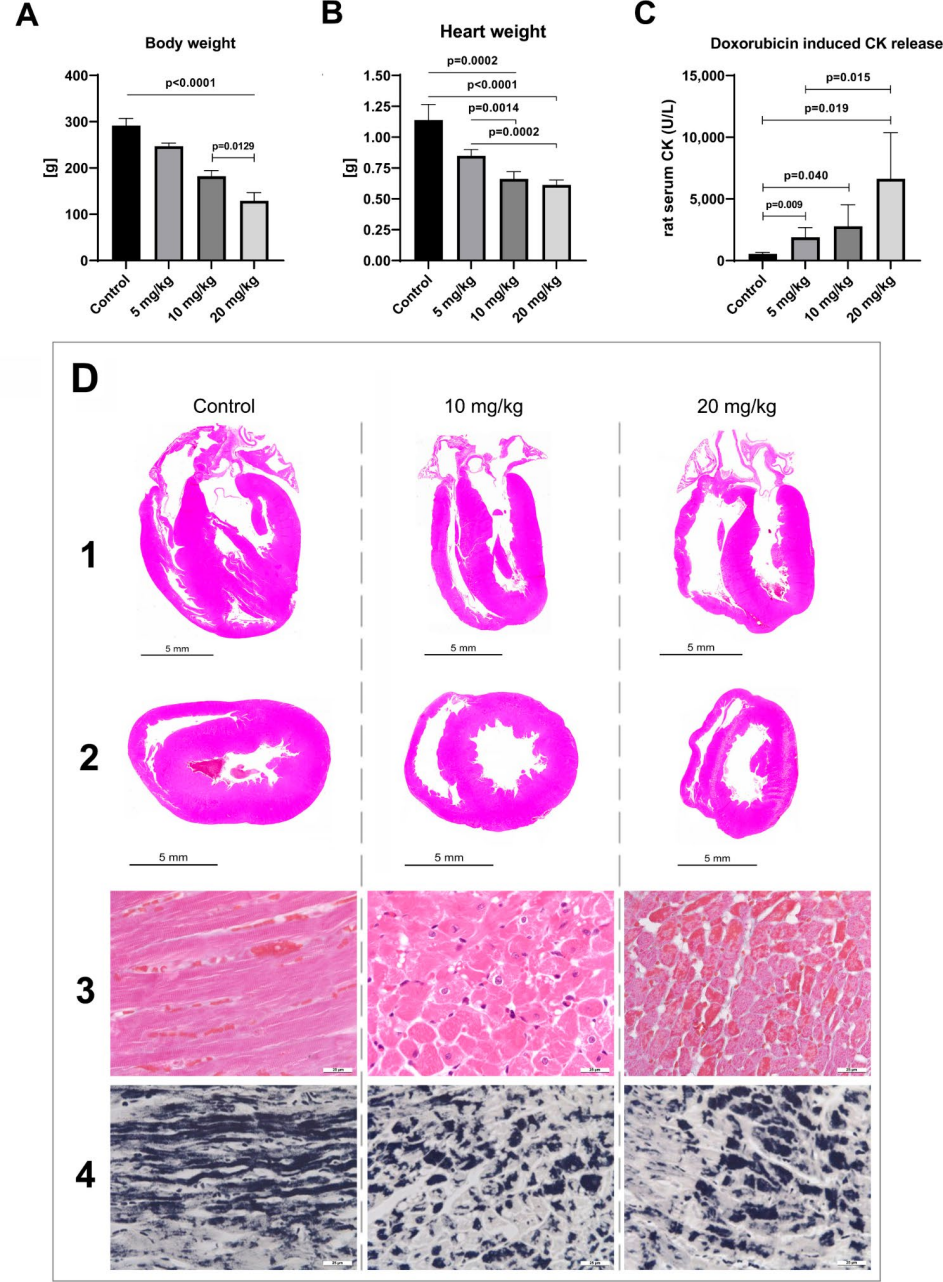
Doxorubicin induced cardiomyopathy: Gross-morphology and cardiac muscle cell striations. Depicted in panels A-C are the clinicopathological features of doxorubicin-treated rats. **Panel A**: Body weight of doxorubicin-treated rats. When compared to controls, doxorubicin treatment caused a clear dose-related and highly significant reduction in body weights. The difference between treatments is also significant. **Panel B**: Heart weight of doxorubicin-treated rats. When compared to controls, the reduction in heart weight is highly significant and dose-related. The difference between treatments is also significant. **Panel C**: Serum creatinine kinase activity. Myocardial injury caused a clear dose-related and extraordinary increase in CK activity. The difference between treatments is also significant. **Panel D** line 1 and 2: Gross morphology of the heart. Depicted are the complete longitudinal (line 1) and cross sections (line 2) of the hearts of control and doxorubicin-treated animals. The hearts of control animals in the longitudinal section (line 1, control) display an ovally contracted chambers in the cross section (line 2, control). Conversely, atrophied hearts of treated animals are characterized by circular and semicircular dilatative shapes and a decreased chamber wall thickness of the left and right ventricules. Note the dose-related changes with a cylindrical and even balloon-like dilated appearance (line 2, 10 and 20 mg/kg). **Panel D** line 3 and 4: Crossmon and Heidenhain’s iron hematoxylin stain. Line 3 (Crossmon stain): With controls, the sarcomers of cardiomyocytes are intact, and the cardiac muscle cell striations are regular. Doxorubicin treatment caused marked damage of the myofilaments with the loss of cross-striations. Severely harmed cardiomyocytes are hallmarked by an intense orange-red stained and lumpy changed cytoplasm (Crossmon stain, line 3, 20 mg/kg). Depicted in line 3, row 2 is an H&E stained section of a 10 mg/kg treated animal. Note the focal vacuolar degeneration of cardiomyocytes and otherwise early band like aggregates of their cytoplasm. Line 4 (Heidenhain’s iron hematoxylin stain): With controls, the cross striations of cardiac muscle cells are well preserved and are stained in black. Following doxorubicin treatment (10 mg/kg and 20 mg/kg) the cross striation of cardiac muscle cells is lost and the fibers are transformed into irregular aggregates

**Fig. 2:**
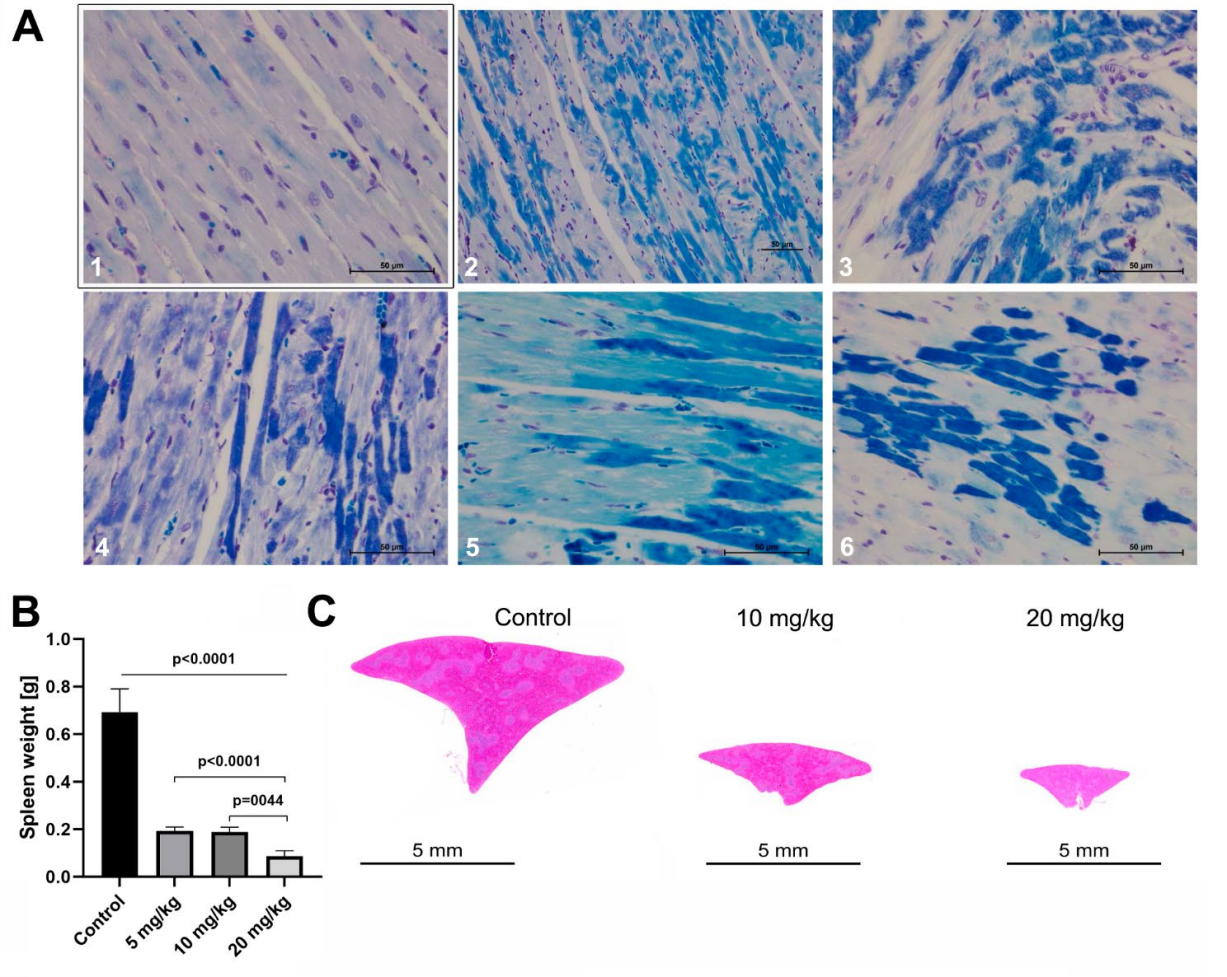
Histopathology of cardiac myofibrillar degeneration, contraction bands and spleen organ toxicity. **Panel A** (Luxol fast blue stain): The LFB stain is a specific marker of myofibrillar degeneration, and harmed cardiomyocytes are stained in different shades of blue. A1 is the section of a control animal, and the dye is not taken up by healthy cardiomyocytes. In strong contrast, doxorubicin treatment (A2-A6) caused myofibrillar degerneration as indicated by the dark blue staining and advanced irregular cytoplasmic coagulations (= contraction band necrosis as the organ specific form of an apoptotic degeneration). The light blue stained cells with their fine granular pattern (A4-A6) are initial stages of cardiomyocyte degeneration and are characterized by the splitting of myofibrils which progress to lumpy aggregates (= contraction bands, dark blue stained cells). Together, the LFB stain reveals the vast extent of myocardial degeneration. **Panel B**: Spleen organ weight. The spleen of rodents is of critical importance in extramedullary hematopoiesis; it therefore enables an assessment of hematoxicity and is extremely sensitive to p53 dependent cell death signaling. When compared to controls, doxorubicin caused a clear dose-related and highly significant reduction in spleen weights. Moreover, the difference in organ weight between treatments is significant. **Panel C**: Gross-morphology of the spleen. Depicted are the complete cross sections of controls and animals dosed at 10 mg/kg and 20 mg/kg. Note the severe dose-dependent atrophy of the spleen

At clinically relevant doses, doxorubicin treatment of rats caused marked cell death as denoted by the highly significant and strict dose-related reduction in body and heart weights (Fig. 1A and B). Especially the T4-treatment caused excessive cardiac injury with 30–40% loss of heart muscle and a clear dose-related significant increase in serum CK activities (Fig. 1C). Additionally, doxorubicin treatment caused dose-dependent toxicity of lung, liver and kidney (supplementary Figure S1). In the case of spleen, the toxicity was drastic, i.e. 70–85% loss in organ weight (Fig. 2B&C). Note, independent research showed differences in tissue sensitivities for p53-dependent cell death programs, with thymus and spleen being extraordinarly sensitive to p53 transcriptional responses [51]. After 6 days of treatment, animals of the T4-treatment presented clinical symptoms of congestive heart failure, i.e. dyspnea with signs of Cheyne-Stokes respiration, fatigue and exercise intolerance, as observed with patients.

Gross morphological examination and histopathology revealed diminished thicknesses of the chamber walls with the left and the right ventricles displaying features of dilative cardiomyopathy (Fig. 1D, lines 1&2). Unlike controls showing a pronounced tip and an oval shaped cross-section of the well-contracted chambers, the hearts of treated animals appeared cylindrical or even balloon-like dilated (Fig. 1D, line 1&2). The changes were dose-related. We observed disseminated foci of advanced degenerative changes of the myocardium predominantly in areas close to the heart tip. The septum seemed more affected than the outer ventricle walls, and the left ventricle gradually more than the right. Cellular changes were obvious anywhere, even within atrial walls.

All doxorubicin-treated animals showed slight to moderate interstitial edema of the myocardium, and after 6 days of treatment, most T4 animals presented clinical signs of heart insufficiency. Meanwhile, T3 animals received the same dose as T4 animals, i.e. 20 mg/kg; however, for 3 instead of 6 days, and the shorter T3 treatment period was less harming. This underpins the importance of cumulative doses in doxorubicin-induced cardiomyopathy.

Shown in Fig. 1D line 3 is the Crossmon stain for a control animal. The sarcomeres are intact as evidenced by the regular cardiac muscle cell striation. In strong contrast, doxorubicin treatment caused marked damage of the myofilaments and the loss of cross-striations. Severely harmed cardiomyocytes are hallmarked by intense orange-red staining of the cytoplasm as exemplified for the 20 mg/kg treatment. Depicted in Fig. 1D line 3 (2nd image, H&E stained cardiac section) is an example of vacuolar degenerated cardiomyocytes with contraction bands. The results for the Heidenhain’s iron hematoxylin stain are depicted in Fig. 1D line 4. With controls, the cross striation of cardiac muscle cells is well preserved and stained in black. Following doxorubicin treatment, the cross striation of cardiomyocytes is lost and the myofibrillar degeneration progressed to lumpy aggregates. To confirm contraction bands, we employed the Luxol fast blue (LFB) stain (Fig. 2A) and observed splitting of myofibrils as shown by diffusely stained cells with fine granular pattern (line 1). Intriguingly, the cellular responses to doxorubicin treatment differed significantly with some cardiomyocytes showing myofibrillar degeneration [30] whereas severely harmed cardiomyocytes are characterized by contraction bands (Fig. 2A). Together, we observed, side-by-side, the various phases of cellular degeneration (Fig. 2A, line 2). However, some cardiomyocytes are more prone to cardiotoxicity than others, and differences in cellular responses are likely due to cardiomyocyte subpopulations. Indeed, a recently published landmark study identified distinct cardiomyocyte populations of ventricles and atria [7]. The fact that up to 40% of cardiac tissue is lost underscores differences in survival/reparative capacity of cardiomyocytes following doxorubicin treatment (see also the differences in transcriptional profiles described below). An advanced myofibrillar degeneration is characterized by narrower and shrunken cardiomyocytes, and we noted regional myocyte disarray while severely harmed cardiomyocytes displayed signs of an initial lysis. The nuclei of harmed cardiomyocytes were either ovoid enlarged and vesicular or shrunken and dense, whereas the pyknotic changes hallmark cellular senescence and contraction bands as part of an apoptotic process. At the time of organ harvest most of the cellular debris were cleared.

Additionally, we observed a drastic dose-dependent splenic atrophy (Fig. 2B and C), and doxorubicin treatment caused hepatotoxicity and nephrotoxicity (supplementary Figure S1).

### Electron microscopy of doxorubicin-induced cardiomyopathy

**Fig. 3:**
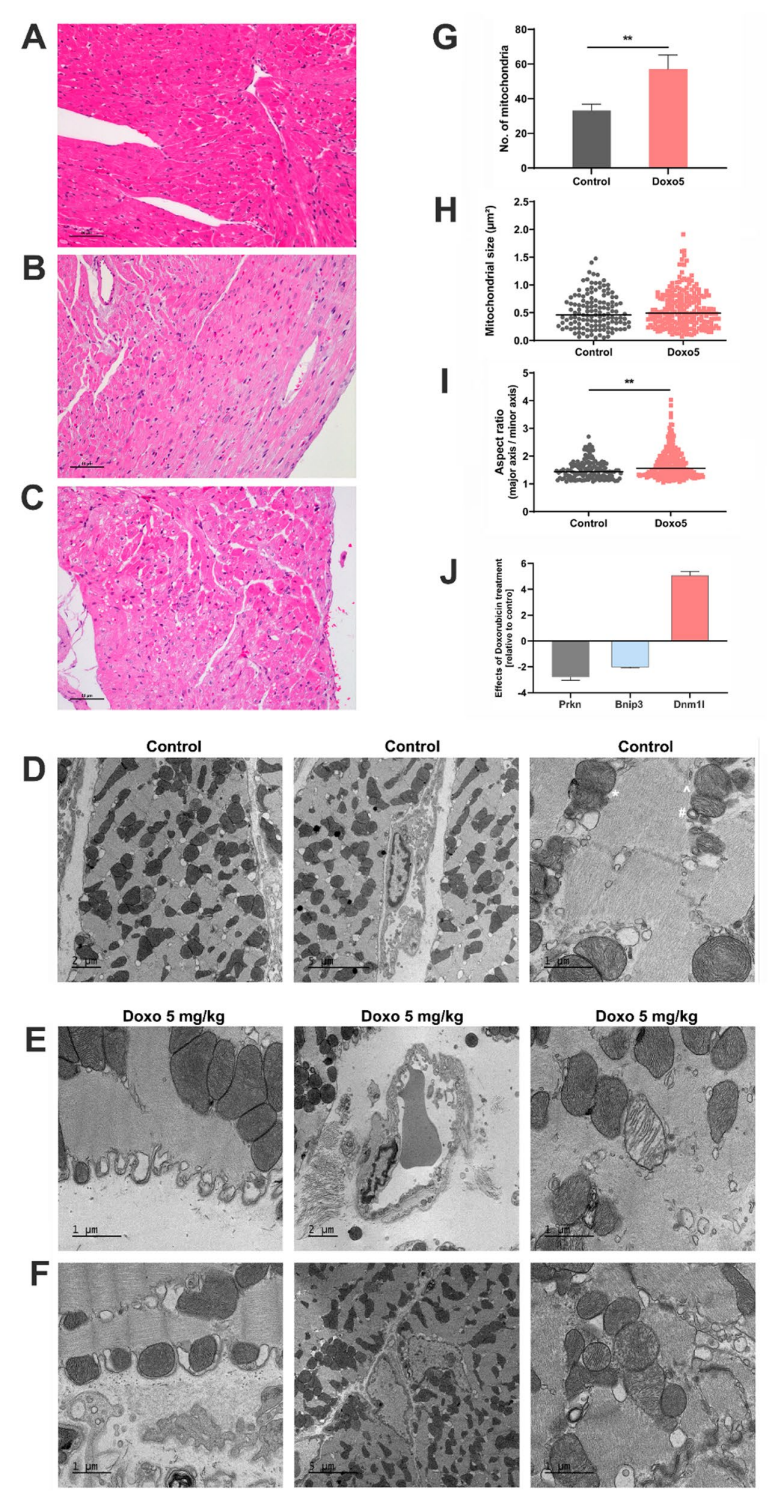
Light and transmission electronmicroscopy of doxorubicin induced cardiomyopathy. Animals were given daily i.p. administrations of 5 mg/kg doxorubicin for five consecutive days. **Panel A**: Hematoxylin & eosin stained left ventricular sections of control rat hearts. The cardiomyocytes are rod-shaped with no sign of myofibrillar damage or change in the calibre of myofibrils. **Panels B**&**C**: H&E stained sections of the heart. The cardiomyocytes are shrunken and vacuolated. A significant proportion of cardiomyocytes displayed pyknotic nuclei (chromatin and nucleus shrinkage) which hallmark cells undergoing programmed cell death. Note the inflammatiory infiltrates and activated macrophages in panel **C**. **Panel D**: Electron micrographs of the heart of control animals. TEM of vehicle treated controls revealed a regular arrangement of the myofibrils; the mitochondria vary in size and shape and form smaller aggregates (D1–3). The contractile apparatus is intact, and the Z bands of the cardiac sarcomere are clearly visible (D3). Especially for larger mitochondria, the demarcation of the inner membrane, the matrix and the cristae are readily visible. Depicted in panel D3 is an ongoing mitochondrial fusion marked by the star (panel D3, *) with connecting cristae between the two mitochondria as well as a case of mitochondrial fission (panel D3 marked by ^). Note the membrane incursions at juxtapositions to mitochondria (panel D3 #). Panel DII highlights a vascular endothelial cell with a regular nucleus. **Panel E**&**F**: Electron micrographs of heart sections of doxorubicin-treated animals. Doxorubicin treatment caused marked ultrastructural changes of cardiomyocytes with sub-sarcolemmal bleb formation (EI) and protrusions of sarcolemma surrounding individual mitochondria (EI, FI). Depicted in panel EII is a swollen vascular endothelial cell (EII) as well as myofibrillar disarray (EIII), which is characterized by the loss of regular cross striations. The distinct organisation of sarcomeres into bands, zones and lines is vanished, and harmed mitochondria display loss of the cristae structure. Some mitochondria appear swollen (EIII, FIII), and autophagic vacuoles are visible. Panel FII depicts an activated and juxtapost fibroblast as part of the wound healing process and scaring of the cardiac muscle. **Panel G**: Histogram of the number of mitochondria per field of view (50 µm^2^) in control and doxorubicin-treated animals. Doxorubicin treatment caused a significant increase in the number of mitochondria. **Panel H**: Scatter plots of the size of mitochondria in control and doxorubicin-treated animals. Doxorubicin treatment did not result in significant changes in the mitochondrial size. **Panel I**: Scatter plots of the aspect ratio which measures the extent of networked mitochondria. Doxorubicin treatment caused a significant increase in the aspect ratio and therefore elicited increased fusions of mitochondria. **Panel J**: Gene expression of Parkin, Bnip3 and dynamin 1 like in cardiac tissue of doxorubicin treated rats. The bar graph shows fold changes relative to vehicle treated controls, and the data are mean ± SD

Shown in Fig. 3 are hematoxylin & eosin stained sections of the left ventricle of control (A) and doxorubicin-treated animals (B, C) in addition to electron micrographs of controls (D) and treated animals (E-F). With controls, the light microscopic examination did not reveal morphological abnormalities. The cardiomyocytes are rod-shaped with no sign of myofibrillar damage or change in the calibre of myofibrils and cell width. Following daily i.p. administrations of 5 mg/kg doxorubicin for 5 days, the cardiomyocytes appeared shrunken, vacuolated, the rod-shaped architecture is lost, and the pyknotic nuclei (chromatin and nucleus shrinkage) earmark cells destined for programmed cell death (Fig. 3B&C).

Clearly visible is an acute inflammatory reaction which is characterized by an endothelial and fibroblast cell activiation in addition to an interstitial edema. Furthermore, different stages of cardiomyocyte apoptosis are seen and apoptotic cardiomyocytes present as focally increased (panel C). Notwithstanding, activated macrophages and lymphocytes are scarcely seen by light microscopy. Note, recent research provided strong evidence for cardiac resident macrophages to function in repair whereas monocytic infiltrates maintain an inflammation-related signaling in doxorubicin induced cardiomyopathy [52]. Collectively, the inflammatory reactions contributed to the pathological sequelae of doxorubicin induced cardiomyopathy.

The ultrastructure of control animals reveals a regular arrangement of the myofibrils; the mitochondria vary in size and shape and form smaller aggregates (D1–3). The contractile apparatus is intact, and the Z bands of the cardiac sarcomere are clearly visible (D3). Especially for larger mitochondria, the demarcation of the inner membrane, the matrix and the cristae are readily visible. Depicted in panel D3 is an example of an ongoing mitochondrial fusion with connecting cristae between the two mitochondria as well as a case of mitochondrial fission. Additionally, there are membrane incursions at juxtapositions to mitochondria. Panel D2 highlights an endothelial cell with a regular nucleus in a capillary.

Doxorubicin treatment caused marked ultrastructural changes of cardiomyocytes (panels E and F) with sub-sarcolemmal bleb formation (E1) and protrusions of sarcolemma surrounding individual mitochondria (E1, F1). Panel E2 and E3 depict a swollen vascular endothelial cell and myofibrillar disarray (E3), which is characterized by the loss of regular cross striations. The distinct organisation of sarcomeres into bands, zones and lines is vanished, and harmed mitochondria display loss of the cristae structure. Some mitochondria appear swollen (E3, F3), and autophagic vacuoles are visible. Shown in panel F2 is a myofibroblast as part of the wound healing process and the scaring of the tissue.

To obtain quantitative data, we counted the number of mitochondria per field of view (50 µm^2^) and determined the mitochondrial size and the aspect ratio, which quantifies the extent of networked mitochondria. A higher aspect ratio implies an increased fusion of mitochondria as originally reported by Luz and colleagues [53]. Following doxorubicin treatment, we observed a significantly increased number of mitochondria in surviving cardiomyocytes (G), and the aspect ratio was likewise increased (I). For the same cardiac tissue, we determined > 5-fold induced expression of dynamin like 1 (Fig. 3J, supplementary Table S5), and this protein is a major regulator of mitochondrial fission. We also observed 3-fold repressed parkin expression (Fig. 3J, supplementary Table S5). Parkin is a p53 target gene and a positive regulator of mitophagy [54]. Note, harmed mitochondria are cleared either by Parkin-mediated ubiquitination or through mitophagy receptors, and to clear damaged mitochondria, the mitophagy receptor Bnip3 utilizes the Rab5-endosomal pathway [55]. We observed opposite regulation of Rab5a in T1 (induced) and T4 treated animals (repressed, supplementary Table S5). Doxorubicin treatment repressed expression of the mitophagy receptor Bnip3 significantly (Fig. 3J, supplementary Table S5). Together, this leads us to propose an impaired mitophagy but increased mitochondrial fission in cardiac cells who survived doxorubicin toxicity.

### Genomics of doxorubicin-induced cardiomyopathy

We performed whole genome scans with RNA isolated from morphologically well-characterized cardiac tissue of control and doxorubicin-treated rats. Based on the criteria of ≥ 2-fold changes and FDR adjusted p-values of < 0.05, we identified 306 diffentially expressed genes of which 181 and 125 were up- and down-regulated (supplementary Table S5). Among the DEGs there are seven genes oppositely regulated in the various treatments (supplementary Table S6). Additionally, we evaluated the gene expression of Hif1alpha by RT-qPCR, which served as a positive control, and this factor is highly inducible upon doxorubicin treatment [56]. The expression of Hif1alpha in heart tissue was significantly (*p* < 0.01) induced, i.e. >8- and > 7-fold, respectively in response to T3 and T4 treatments.

**Fig. 4:**
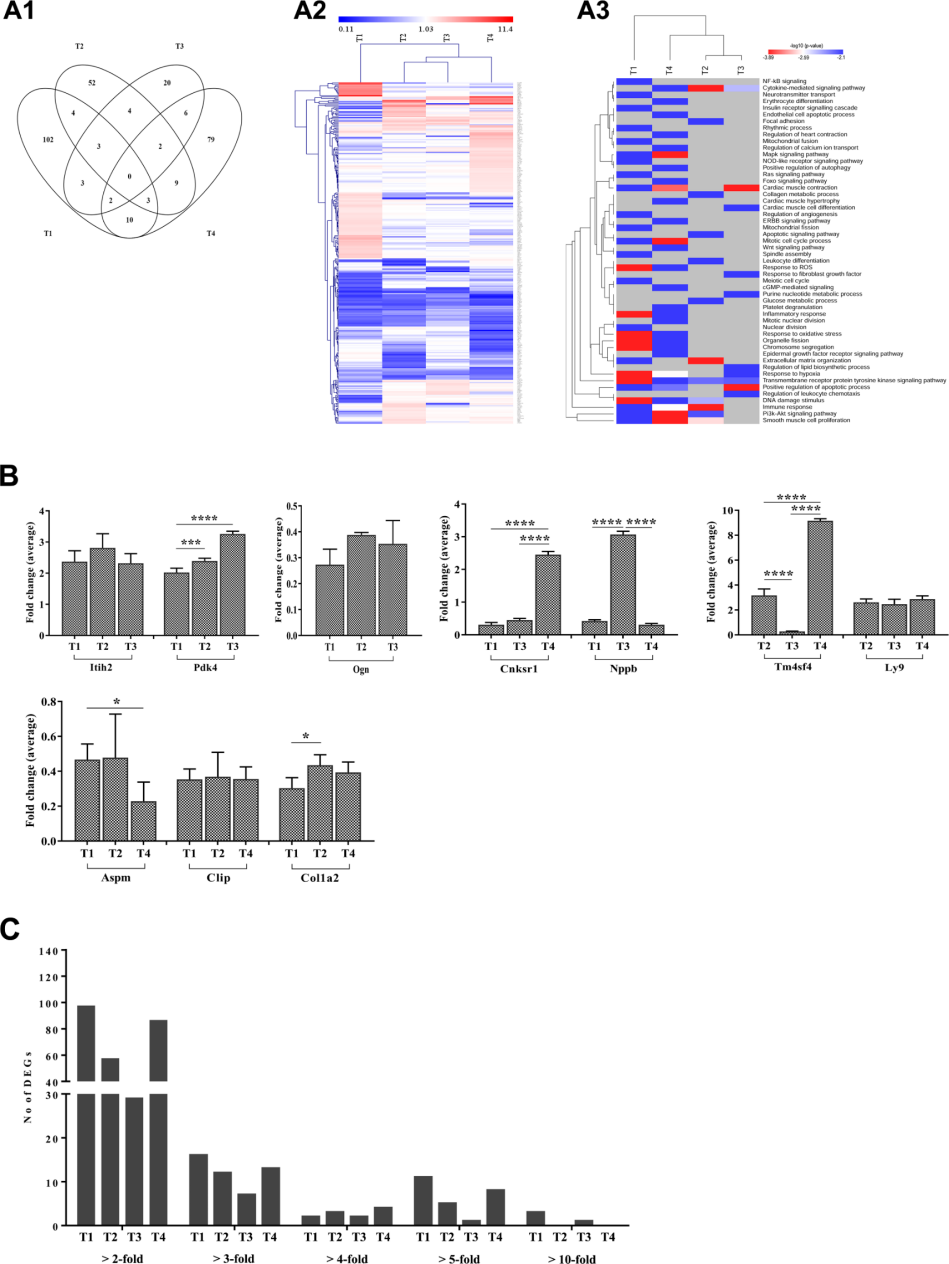
Genomic responses of the heart following doxorubicin treatment. **Panel A**: Differentially expressed genes and enriched biological processes after doxorubicin treatment. (A1) Venn diagram of common and doxorubicin treatment specific (T1-T4) DEGs in the heart. (A2) Heatmap of differentially expressed genes. The heatmap was generated with an average-linkage Euclidean distance hierarchical clustering algorithm, and depicted is the fold change for DEGs. (A3) Heatmap of significantly enriched biological processes (p-value < 0.01) for treatment specific DEGs. Gene ontologies are Metascape web-tool based. **Panel B**: Commonly regulated genes in cardiac tissue among the different treatment groups. **Panel C**: Magnitude of gene expression changes and their frequency in the different treatment groups

Figure 4A1 depicts a Venn diagram of the transcriptomic data, the gene cluster analysis for co-regulated genes (Fig. 4A2) and enriched gene ontology terms (Fig. 4A3). Based on the expression pattern of co-regulated genes the T1-T4 treatments are clearly segregated (Fig. 4A2). Notwithstanding, co-expressed genes of the T3 and T4 regimen are more closely related, and both treatments share the same dose, i.e. 20 mg/kg, but differ in the treatment duration, i.e. 3 versus 6 days. Together, the four treatments (T1-T4) resulted in 102, 52, 20 and 79 unique and therefore distinct gene regulations with no overlap between them (Fig. 4A1, Venn diagram and supplementary Table S5). Nonetheless, we identified up to 3 commonly regulated genes in the comparison of three different treatments (Fig. 4B), whereas the results of a comparison between two treatments are given in supplementary Figure S2. Some genes are oppositely regulated in the various treatments. For instance, T2 caused repressed and T4 induced expression of dedicator of cytokinesis protein 9 (Dock 9), i.e. a protein highly expressed in heart tissue that functions in the signaling networks of small G proteins. Collectively, we observed strict treatment dependent genomic responses with little overlap between the different regimens, and the results imply considerable heterogeneity in cardiomyocyte responses to doxorubicin treatment. Importantly, a recently published single cell RNAseq study reported cardiomyocyte subpopulations and the existence of different cardiomyocyte populations even within anatomical location, i.e. atria versus ventricle [7]. Therefore, we regard differences in transcriptional responses as indication for cardiomyocyte subpopulations which differed in their vulnerability to doxorubicin treatments.

Figure 4C depicts a histogram of gene expression changes among the four treatments, and even at the lowest dose of 1 mg/kg (T1) we observed robust gene regulations with nearly 9% of DEGs > 5 to 10-fold regulated. For comparison, about 7% of DEGs were regulated to a similar extent at the 20 mg/kg (T4) regimen. We linked clinical signs of cardiomyopathy to genomic responses, and this provided a rationale for defining MTDs (= maximal tolerated doses with reduced risk for cardiotoxicity). As described above, histopathology defined damages of the myofibrillar apparatus, contraction bands and the vacuolization of cardiomyocytes. Given the marked clinical signs of T4 animals, we explored the genomic data for possible links to heart insufficiency. Among the various disease candidate genes, we noted atrial natriuretic peptide, myosin heavy chain 6, myosin light chain 7, troponin 2, cytochrome c oxidase and the heart morphogenesis regulators Hopx homeobox and T-box transcription factor 6 as highly regulated (supplementary Table S5). Additionally, the marked upregulation of platelet factor 4 (> 4-fold) signifies risk for thromboembolic events as seen in clinical cases, and owing to its role in coronary artery disease and myocardial infarction, the highly significant repression of the phosphatase and actin regulator is another remarkable finding [57]. Moreover, epidermal growth factor (EGF) is > 3-fold induced (supplementary Table S5) which signals through the EGF receptor and is of critical importance in heart development and function [58]. EGFR was reported to be highly regulated in patients with atrial fibrillation [59]. Conversely, the > 3-fold induced expression of angiopoietin like 4 facilitates macrophage polarization to support cardiac repair as demonstrated in inflammation-related animal models [60]. These examples highlight the knowledge gain from a genomic study to investigate toxic cardiomyopathy, and we report the results of the gene set enrichment analysis in supplementary Table S7.

**Fig. 5:**
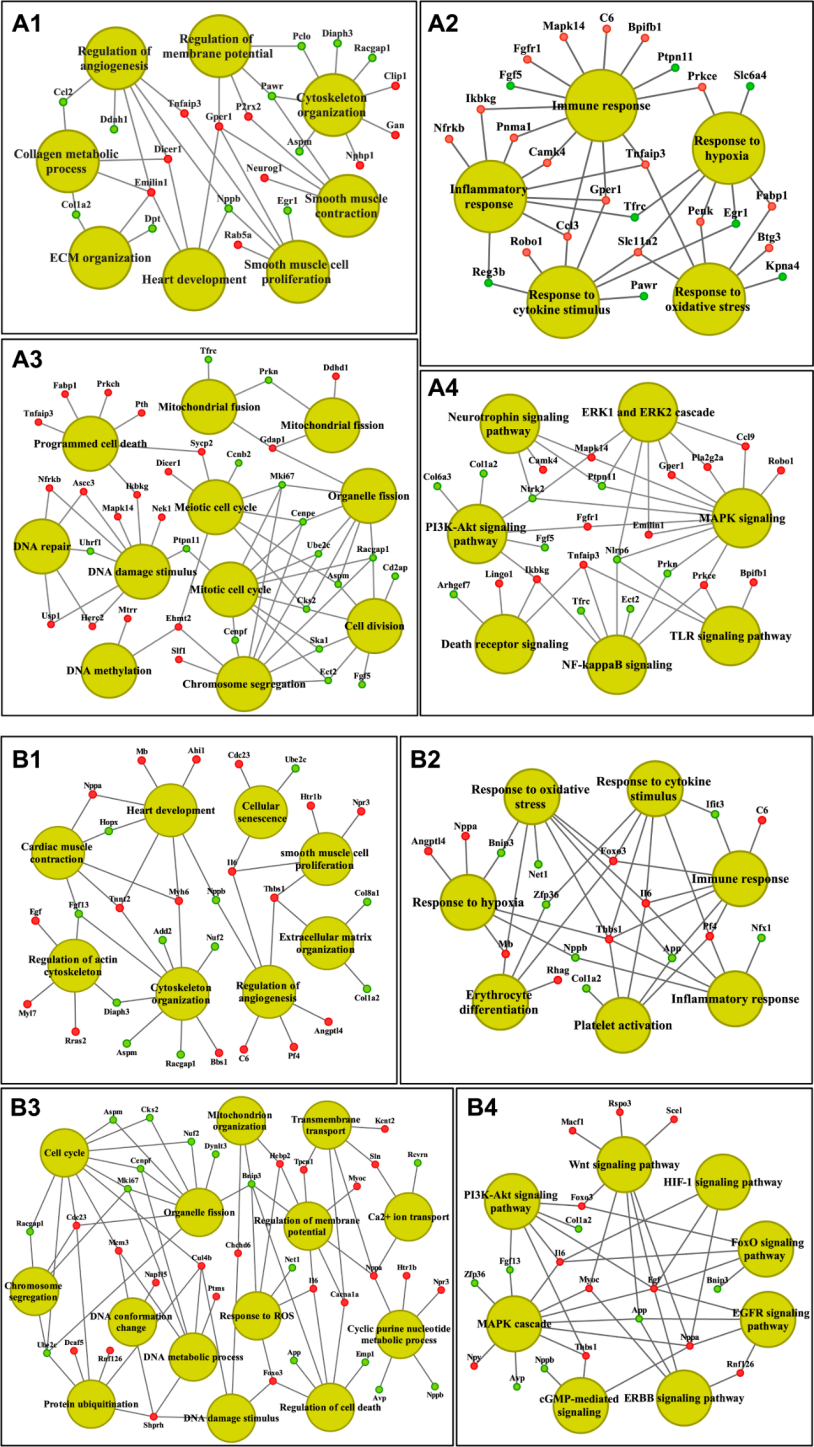
Gene enrichment and pathway mapping of doxorubicin responsive cardiac genes. The enriched biological terms and pathways were computed with the ClueGO plugin of Cytoscape version 3.9. The red and green color nodes illustrate up- and down-regulated genes. **Panel A**: T1 (1 mg/kg for 3 days) regulated genes. (A1) Biological processes associated with cardiac muscle contraction. (A2) Immune and inflammation response genes and their associated network. (A3) Cell cycle and apoptotic processes gene network A4) A regulatory network of signaling cascades. **Panel B**) T4 (20 mg/kg for 6 days) regulated genes. (B1) Biological processes associated with cardiac muscle contraction. (B2) Immune and inflammation response genes and their associated network. (B3) Cell cycle and apoptotic processes gene network. (B4) A regulatory network of signaling cascades

We compile significantly regulated genes associated with the pathogenic sequelae of doxorubicin-induced cardiomyopathy in supplementary Table S5, and Fig. 4A3 depicts the corresponding clusters of enriched ontology terms. T1 is well separated from the other treatments. Likewise, T4 clearly segregates from T2 and T3. Here, highly enriched terms are cardiac muscle cell development, cardiac muscle contraction and hyperthrophy, cell death and cellular senescence, mitochondrial fission, mitochondrial organization, regulation of membrane potential, calcium ion transport, apoptotic signaling, protein ubiququitination and cytoskeleton organisation. Additional terms of high relevance are DNA conformation changes, chromosome organisation, DNA repair, response to DNA damage stimulus, DNA methylation as well as immune and inflammatory response, response to cytokine stimulus and MAPK signaling. Figure 5 shows the genes associated with specific ontology terms for T1 (1 mg/kg for 3 days, panels A1-A4) and T4 (20 mg/kg for 6 days, panels B1-B4) with a focus on cardiac muscle contraction (panel A1/B1), immune and inflammation response genes and their associated network (panel A2/B2), cell cycle and apoptotic processes gene network (panel A3/B3) and signaling networks (panel A4/B4).

### Validation of doxorubicin-regulated genes in cell lines and iPS-derived human cardiomyocytes

**Fig. 6:**
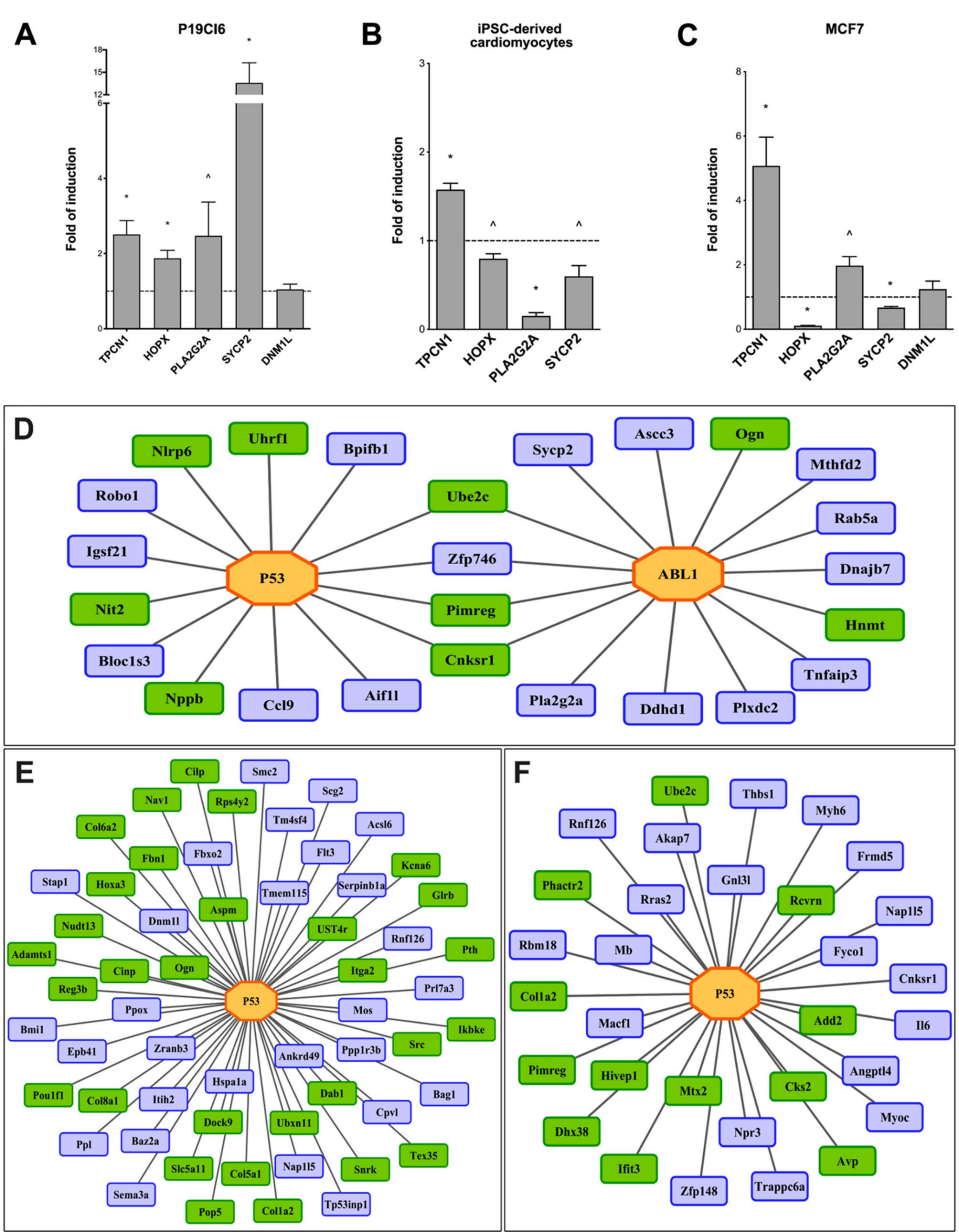
qPCR validation of doxorubicin responsive genes in cell lines and iPS-derived human cardiomyocytes and networks of p53 and Abl1 regulated genes in the heart. Based on the genomic scans of heart tissue, we selected five doxorubicin responsive genes for validation. The results were obtained by the ∆C_t_ method, and the data are fold-changes relative to controls. GAPDH and B2M, respectively served as normalizers for mouse or human cells. **Panel A**: P19Cl6 mouse cells differentiated into a cardiomyocyte phenotype. The cells were cultured as described in the method section and treated with 0.5µM doxorubicin for 16 h. **Panel B**: iPS-derived human cardyomyocytes. The cells were cultured as described in the method section and treated with 0.1 µM doxorubicin for 16 h. **Panel C**: MCF7, p53-wild-type expressing breast adenocarcinoma-derived human cell line. The cells were cultured as described in the method section and treated with 1.5µM doxorubicin for 16 h. The bar charts are averages and standard deviations of three biological replicates. * = *p* < 0.01 and ^ = *p* < 0.05 Student t-test. **Panel D**-**F**: p53 and Abl1 enriched binding sites in promoters of doxorubicin regulated genes. Enriched p53 and Abl1 binding sites were identified within promoters of doxorubicin responsive genes by considering a length of -1000 to + 100 base pairs relative to the transcription start site (TSS). (**D**) Abl1 and p53 regulatory gene network following 1 mg/kg doxorubicin treatment for 3 days. (**E**) A network of p53 target genes following 10 mg/kg doxorubicin treatment for 3 days. (**F**) A network of p53 target genes following 20 mg/kg doxorubicin treatment for 6 days. The green color refers to repressed and the blue for upregulated genes

We employed different mouse and human cell models to validate doxorubicin-induced gene expression changes by RT-qPCR (Fig. 6A-C) and selected genes based on their regulation at different time points and biological functions in cardiomyocytes. The selected genes contain Abl1 and p53 binding sites and are specifically upregulated in T1 (Pla2g2a, Sycp2), T2 (Dnm1l) and T3/T4 (Tpcn1). Conversely, Hopx is specifically regulated in T4 and repressed following doxorubicin treatment. Apart from the fact that the genes contain consensus binding sites for Abl1 and p53, we selected these genes for the following reasons: Tpcn1 is a member of the voltage-gated two-pore ion channels and contributes to the release of Ca^2+^ from the endoplasmic reticulum [61]. The gene is abundantly expressed in heart and kidney [62], and its increased expression correlates with heart failure [63]. We queried the Fischer database of p53 regulated genes [37] and found 18 human cell lines with p53 dependent regulation of Tpcn1. Moroever, p53 occupancy in the TSS enhancer region of Tpcn1 was confirmed by ChIPseq [37], supplementary Table S8). In the same way, the p53 dependent gene regulation of Dnm1l was confirmed in 9 human cell lines and was also regulated in human cell lines which express a variant of the p53-related transcription factor p63 ((36), supplementary Table S8). Specifically, Dnm1l is a mitochondrial fission protein and functions in mitochondrial fragmentation and cell death in response to ischemia of cardiomyocytes [64], while a dominant Dnm1l mutation causes abnormal cardiac morphology and aberrant energy production in cardiomyopathy [65].

Furthermore, genetic studies in mice demonstrated phospholipase Pla2g2a to promote myocardial injury in response to ischemia and oxidative damage [66], and we selected Sycp2 for its increased expression in cardiomyopathy [67]. Corroborative evidence for the p53 dependent gene regulation of Pla2g2a and Sycp2 stems from the Fischer database and includes 5 and 14 human cell lines, respectively (supplementary Table S8).

Finally, we selected Hopx (also named as Giig15b, Hod, Hop) for validation studies. Hopx is T4 specific, and its p53-dependent repression was reported in different human cell lines and cells expressing the p53-related transcription factor p63 (supplementary Table S8). Hopx is the rat orthologous gene of human HOP, i.e. an atypical homeodomain protein which lacks DNA binding activity; nonetheless, it modulates activity of the serum response factor in the control of cardiac-specific genes by recruiting chromatin modifiers. In fact, the balance between cardiomyocyte proliferation and differentiation is disturbed in Hopx-deficient neonatal mice [68]. Moreover, Hopx modulates Gata 4 acetylation and negatively influences embryonic cardiac myocyte proliferation [69].

To delineate their functional importance, we first measured the expression of these genes in the P19Cl6 mouse cell line (Fig. 6A). We cultured the cells for six days in 1% DMSO, and this condition induced cardiomyocyte-like differentiation [70]. Doxorubicin treatment led to an upregulation of Tpcn1, Pla2g2a, Hopx and especially Sycp2, and apart from Hopx, the results agree with findings in cardiac tissue of doxorubicin-treated rats. Notwithstanding Dnm1l did not change in P19Cl6 cells (Fig. 6A).

Subsequently, we moved to human cellular systems and first, measured gene expression in iPS-derived human cardiomyocytes. We confirmed upregulation of Tpcn1 and repression of Hopx. However, the regulation of Pla2g2a and Sycp2 in iPS-derived human cardiomyocytes is opposite to that of rat heart tissue (Fig. 6B), and this points to species and cell type specific differences in the regulation of p53-target genes. Importantly, the regulation of > 1000 p53-target genes differ between the human and mouse genome [37]. Furthermore, we measured the regulation of these genes in the breast-cancer derived MCF7 cell line in response to doxorubicin treatment. We confirmed induction of Tpcn1 and Pla2g2a and repression of Hopx. Therefore, regulation of these genes in MCF7 cells agreed with results of heart tissue. However, unlike heart and the P19Cl6 cell data, Sycp2 was repressed (Fig. 6C), and Dnm1l was mildly but insignificantly upregulated.

Together, the voltage-gated calcium channel Tpcn1 was consistently upregulated in the different cellular systems, and this underpins its conserved role as a direct p53 target across different species. Similarly, we confirmed repression of Hopx in human iPS-derived cardiomyocytes and the human breast cancer cell line MCF7. According to some investigators, the p53-dependent gene repression requires the p53-p21-DREAM pathway [37].

### Search for regulatory gene networks reveales Abl1 binding sites

To reduce the complexity of genomic data, we performed genome-wide footprints and searched for overrepresented transcription factor binding sites in promoters of doxorubicin-regulated genes. We considered promoters of a length of -1000 to + 100 base pairs relative to the transcription start site (TSS) and used this information to infer regulatory gene networks in drug-induced cardiomyopathy. We considered RefSeq annotated genes only (http://www.ncbi.nlm.nih.gov/RefSeq/) and employed three different promoter sets to search for Abl1 binding sites, i.e. genes which are regulated by at least 4-fold (15 promoters) or > 2-fold (85 promoters) and used 97 promoters of non-regulated genes as controls. In regards to the > 4 and > 2-fold regulated genes about 80 and 50% of promoters were significantly enriched for Abl1 binding sites. Similar results were obtained for p53, and the genomic footprints mapped 100% and 87% of p53 binding sites for > 4- and > 2-fold regulated genes. Figure 6D shows cardiac DEGs targeted by Abl1 and p53, and supplementary Table S8 compiles independent evidence for their p53 dependent regulation in various human and mouse cell lines. Additionally, Fig. 6E&F depicts the networks of p53 target genes following treatment doses of 10 and 20 mg/kg. Apart from Abl1 and p53 binding sites, genomic footprints also discovered significantly enriched cardiac-specific transcription factors in promoters of doxorubicin regulated genes, i.e. GATA4, NKX2.5, MEF2c, TBX5. These findings are not unexpected, given their role in cardiac biology. For instance, the frequency of NKX2.5 binding sites in promoters of doxorubicin regulated genes increased from 3.2 to 4.6 per 1000 bp (*p* < 0.001).

**Fig. 7:**
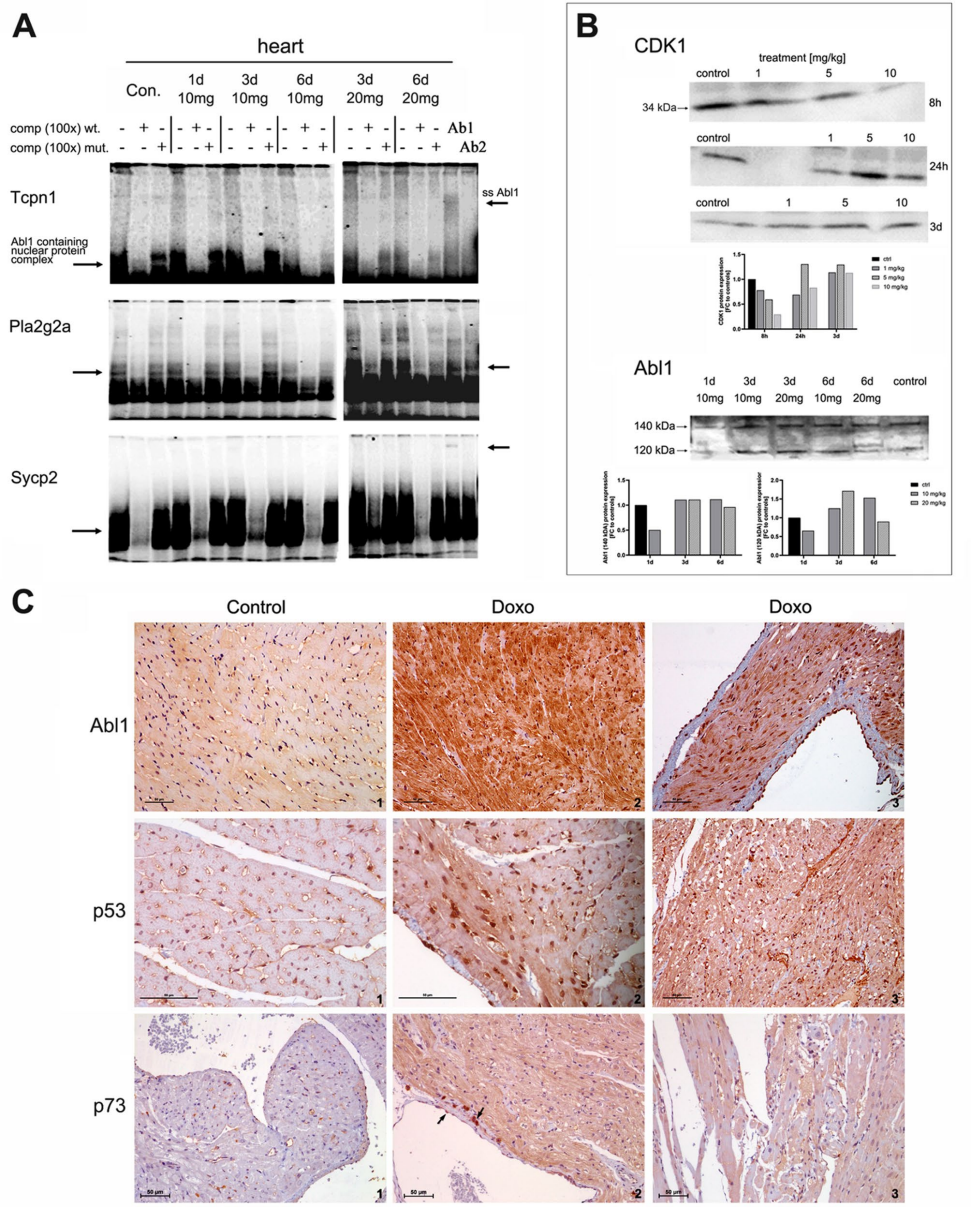
DNA binding activity of Abl1 on p53 targeted genes in the heart of doxorubicin-treated animals. **Panels A**: EMSA with nuclear protein extracts of cardiac tissue. We performed gel electrophoresis mobility shift assays with oligoprobes that harbour gene specific promoter binding sites for Abl1. Depicted are autoradiographs of heart nuclear protein complexes which were isolated from control and doxorubicin-treated animals. The 32P-labeled oligo probes are specific for Tcpn1, Pla2g2a and Sycp2, and competition assays were done with perfect matched (wt) and mutated probes (mut). Supershifts were carried out with two different antibodies which recognize Abl1 as detailed in the method section. In competition assays with 100-fold excess of the wt-probe the binding of the nuclear protein is significantly diminished whereas the mutated probes failed to shift the band. Furthermore, in super-shift assays, the antibody recognized Abl1 protein within the nuclear protein complex, and as detailed in the results section, variable amounts of the Abl1 protein were super-shifted. **Panel B**: Western blots of CDK1 and Abl1 protein. Immunoblots were performed with pooled nucler protein extracts (*N* = 3 animals) of cardiac tissue from control and doxorubicin-treated animals. We observed a clear dose-related decline in CDK1 protein after 8 h of treatment which returned to control values and slightly above after 24 and 72 h. The antibody for Abl1 recognized a 120 kDa and 140 kDa band. Initially, Abl1 expression was reduced and subsequently increased after 3 and 6 days. Note the dose-related differences in nuclear Abl1 protein expression. **Panel C**: Regulation of Abl1, p53 and p73 protein in the heart of control and doxorubicin-treated rats. We performed IHC with cardiac tissue of control and doxorubicin-treated animals (10 mg/kg for 6 days) and found Abl1 expression to be minimal to slight in cardiomyocytes of control animals. Conversely, its cytosolic expression in ventricular cardiomyocytes is marked (image 2), and we observed prominent nuclear as well as cytosolic expression of Abl1 in atrial cardiomyocytes (image 3). Cardiomyocytes of control animals do not express p53. In strong contrast, doxorubicin treatment elicited marked nuclear (image 2) and cytosolic expression of p53 in ventricular and atrial cardiomyocytes. Although p73 is not expressed in cardiomyocytes of control animals, it is strongly induced in atrial cardiomyocytes, in mast and endothelial cells (marked by the arrow, image 2). Strikingly, p73 expression is minimally in ventricular tissue and confined to a subpopulation of cardiomyocytes. Together, the strong induction of the proteins is highly suggestive for an activated Abl1-p53/p73 signaling axis in doxorubicin-dependent cardiomyopathies

To verify the computationally mapped Abl1 and p53 genomic footprints, we performed electrophoretic mobility shift assays (EMSA) with nuclear extracts of heart (Fig. 7) and liver tissue of control and doxorubicin-treated animals and rat hepatocyte cultures (supplementary Figure S3 and S4). We designed oligonucleotide probes which contained Abl1 binding sites in gene specific promoter sequences of Pla2g2a, Sycp2, Tpcn and Hopx (sequences are given in supplementary Table S2).

The EMSA band shifts with nuclear extracts of cardiac tissue confirmed the presence of Abl1 in competition assays with gene specific oligonucleotide probes whereas the use of mutated probes demonstrates specificity (Fig. 7A). Only a small fraction was supershifted with the Abl1 antibodies Ab1 and Ab2, and the results suggest that Abl1 is part of a multi-nuclear protein complex that binds to AT-rich Abl1 consensus sites while epitope masking may have contributed to an underestimation of Abl1 abundance in the probe-shifting complexes.

Shown in Fig. 7B are Western blots (WB) of CDK1 and Abl1. CDK1 phosphorylates Abl interactor (Abi) at serine 216, and this posttranslational modification attenuates Abl1 tyrosine kinase activity through an allosteric mechanism [71, 72]. We observed a strict dose-dependent decline in CDK1 protein expression in nuclear extracts of heart tissue after 8 h of treatment, and with the exception of the 5 mg dose, CDK1 protein levels remained repressed at 24 h but returned to normal after 3 days (Fig. 7B; pool of *N* = 3 animals). Given its function on Abi and its role in the regulation of Abl1 tyrosine kinase activity, CDK1 repression can be regarded as an acute response to stimulate Abl1 activity. Furthermore, cyclin B transcripts levels were significantly repressed in T1 treated animals (supplementary Table S5), and this cyclin forms a complex with CDK1 to actively phosphorylate Abl interactor. Therefore, the coordinate repression of CDK1 and Cyclin B is a critical step to stimulate Abl1 tyrosine kinase activity in response to doxorubicin treatment.

In nuclear extracts of heart tissue, expression of Abl1 was initially reduced (Fig. 7B). We observed two immunoreactive bands, and the 140 kDa band returned to control levels following treatment for 3 and 6 days (left panel). Conversely, expression of the 120 kDa band increased after 3 and 6 days of treatment (right panel). Interestingly, transcript expression of protein tyrosine phosphatase PTPN11 (= SHP2) was significantly repressed in T1 treated animals (supplementary Table S5), and the coded protein inhibits Abl1 tyrosine kinase activity [71].

We observed minimal to slight Abl1 cytosolic expression in cardiomyocytes of control animals (Fig. 7C). Strikingly, doxorubicin treatment caused marked Abl1 cytosolic expression and its nuclear translocation in atrial and ventriclular cardiomyocytes. We did not observe expression of p53 in cardiomyocytes of control animals but noted strong nuclear and cytosolic expression of p53 in atrial and ventricular cardiomyocytes following doxorubicin treatment. Similarly, p73 is not expressed in cardiomyocytes of control animals and only minimal to slightly regulated in ventricular cardiomyocytes of doxorubicin-treated animals. Conversely, its cytosolic expression is marked in atrial cardiomyocytes. Therefore, atrial and ventricular cardiomyocytes differed in p73 signaling in response to doxorubicin treatment, and although Abl1 is extraordinarily induced in ventricular cardiomyocytes, p73 is hardly changed with some ventricular cardiomyocytes showing minimal to slight regulation of p73. Noteworthy are the p73 immunoreactive mast cells and the p73 positive endothelial cells in atria of the heart following doxorubicin treatment (Fig. 7C, marked by an arrow).

**Fig. 8:**
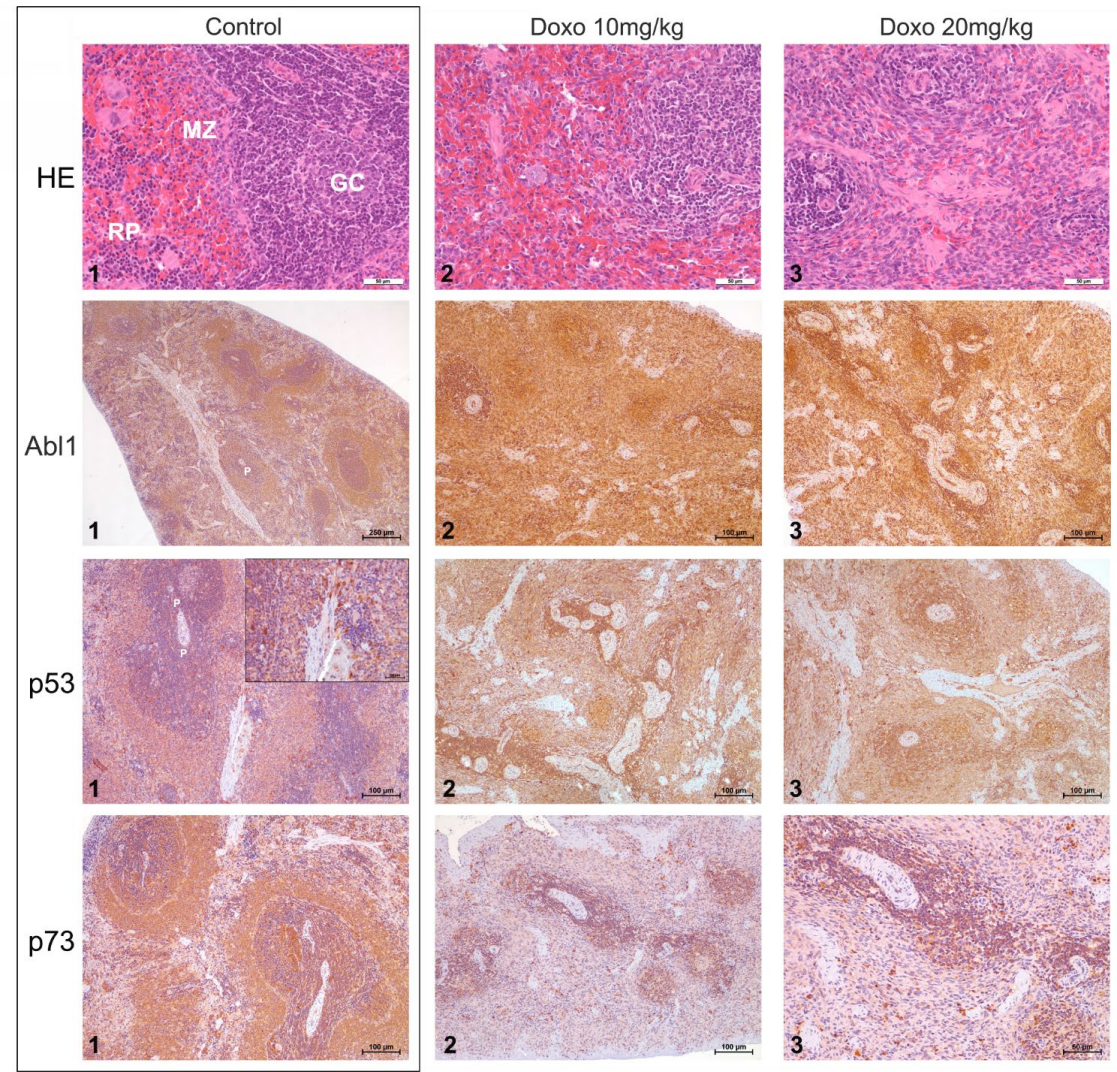
Histopathology and immunohistochemistry of Abl1, p53 and p73 in the spleen of doxorubicin treated rats. **Panel H&E stain**. Depicted is the histology of a normal spleen (row 1) with a lymph follicle (white pulp) and an active Germinal center (GC) and continuously circulating marginal zone (MZ). The red pulp (RP) displays normal trilinear hematopoiesis as indicated by a large number of mature megacaryocytes. Doxorubicin treatment caused severe and dose-dependent hematotoxicity resulting in significant reductions of hematopoiesis and lymphocytopoiesis (row 2&3). The high-dose caused nearly complete loss of hematopoiesis, and the red pulp together with the outskirts of the white pulp is mainly characterized by stroma (row 3). The Mantel and Marginal zone of the white pulp compartment is shrunken and the Germinal centers are almost vanished. Together, doxorubicin caused an extreme atrophy of the lymph follicles. Additionally, the periarteriolar lymphoid sheaths (PALS, labeled as P), which are rich in T-lymphocytes, are shrunken. **Panel Abl1**: Shown is the IHC of a control animal. We observed moderate Abl1 expression of cells in the Marginal zone of white pulp follicles which consist of macrophages, B & T lymphocytes and dendritic cells. Note the scattered expression of cells in the red pulp hematopoetic tissue. Doxorubicin treatment caused excessive Abl1 expression throughout the entire organ that was dose independent. **Panel p53**: Shown is the section of a control animal, and IHC revealed moderate p53 immunoreactive cells in the Marginal zone. The speckeld appearance of some intensely stained cells in the Mantel zone and the red pulp compartment are likely lymphocytes, macrophages and stem cells. Notwithstanding, cells of the Germinal center do not or only minimally express p53. Doxorubicin treatment caused strong p53 expression throughout the entire organ that was dose independent. **Panel p73**: Shown is the section of a control animal, and IHC revealed marked expression of p73 immunoreactive cells in the Marginal and Mantel zone as well as spotty areas within the red pulp compartment. Note the marked expression of p73 in a focal area within the Germinal center. Except for nest-like remnants of the Marginal and Mantel zone with slight expression of p73 doxorubicin caused almost complete ablation of p73 expression. Notwithstanding, sites of erythropoiesis within the red pulp retained slight to moderate p73 expression. Intermingled are presumably erythroid progenitors with marked expression of the protein

Another finding of critical importance relates to the spleen. Importantly, p53 dynamics vary between tissues, and the spleen is highly sensitive to p53 programmed cell death [73]. Furthermore, the spleen plays an essential role in hematopoiesis of rodents, and doxorubicin elicits hematotoxicity. We observed marked changes in the pulp compartments, and the results signify severe haematotoxicity at 10 mg/kg with nearly complete loss of trilinear hematopoiesis at the 20 mg/kg dose (Fig. 8 HE staining). Corroborative evidence stems from blood smears with marked repression of reticulocyte, lymphocyte and white blood cell count. Additionally, we evaluated Abl1 and p53/p73 expression by immunohistochemistry, and for splenic tissue of control animals, we observed moderate Abl1 and p53 but marked p73 expression in cells of the marginal zone (Fig. 8). This zone is composed of macrophages, B & T lymphocytes and dendritic cells [74]. We therefore demonstrate an important physiological role of the Abl1 tyrosine kinase and of p53 and p73 in leukocyte biology that is independent of its function in programmed cell death. Extraordinarily and unlike p53, we observed p73 immunreactive cells in germinal centers of the spleen. This reinforces the notion of an, as yet, undefined physiological role of p73 in the cellular differentiation of lymphocytes of control animals.

Doxorubicin treatment caused excessive Abl1 and p53 expression. The mantel and marginal zone compartments of the white pulp of the spleen is shrunken, and the germinal centers are vanished. Likewise, the periarteriolar lymphoid sheaths (PALS) known to be rich in T-lymphocytes are shrunken. The red pulp compartment is likewise shrunken, and the hemopoetic tissue, as seen with controls, is widely lost. A similar picture emerges with the regulation of p73. Unlike controls, its expression is almost completely ablated following doxorubicin treatment. The germinal centers are vanished, and the marginal zone is considerably shrunken with nests of small aggregates of p73 positive cells. Note the speckled appearance of p73 and, in part, p53 positive immune cells (presumably macrophages and plasma cells) within the red pulp of the spleen of doxorubicin-treated animals.

Apart from the spleen toxicity, we observed hepatotoxicity and therefore investigated Abl1 DNA binding activity in liver tissue nuclear extracts of doxorubicin-treated animals and in cultures of hepatocytes. The results are shown in supplementary Figure S3&S4, and the band shift assays confirmed Abl1 DNA binding activity at gene specific promoters of Pla2g2a, Sycp2, Tpcn and Hopx (supplementary Figure S4A & S4B). However, only a small fraction was supershifted with an Abl1 antibody. Therefore, Abl1 is part of a multiprotein complex that binds to AT-rich Abl1 consensus sites. Additionally, we performed WB experiments (supplementary Figure S4C, pool of *N* = 3 animals), and except for day 1 and the 120 kDa band, Abl1 expression was unchanged. In strong contrast, p53 protein was highly induced (> 30-fold) whereas CDK1 and p73 were minimally upregulated. Together, doxorubicin treatment caused similar regulation of p53 as seen in cardiac tissue.

### Abl1 is a cofactor for p53-induced transactivation

The role of c-Abl in the DNA damage stress response has been the subject of an earlier report [75]. However, there is no definitive evidence for Abl1 to act as a sequence specific transcription factor. Therefore, we developed a one-hybrid yeast-based assay for an inducible expression of full-length Abl1 protein fused to a chimeric transactivation domain with reporter strains containing the Abl1 consensus sequence upstream of a minimal promoter. Although we achieved abundant Abl1 expression and observed nuclear translocation of Abl1 (supplementary Figure S5A-S5C), we did not detect modulation of the reporter upon induced expression of Abl1 (supplementary Figure S5D).

**Fig. 9:**
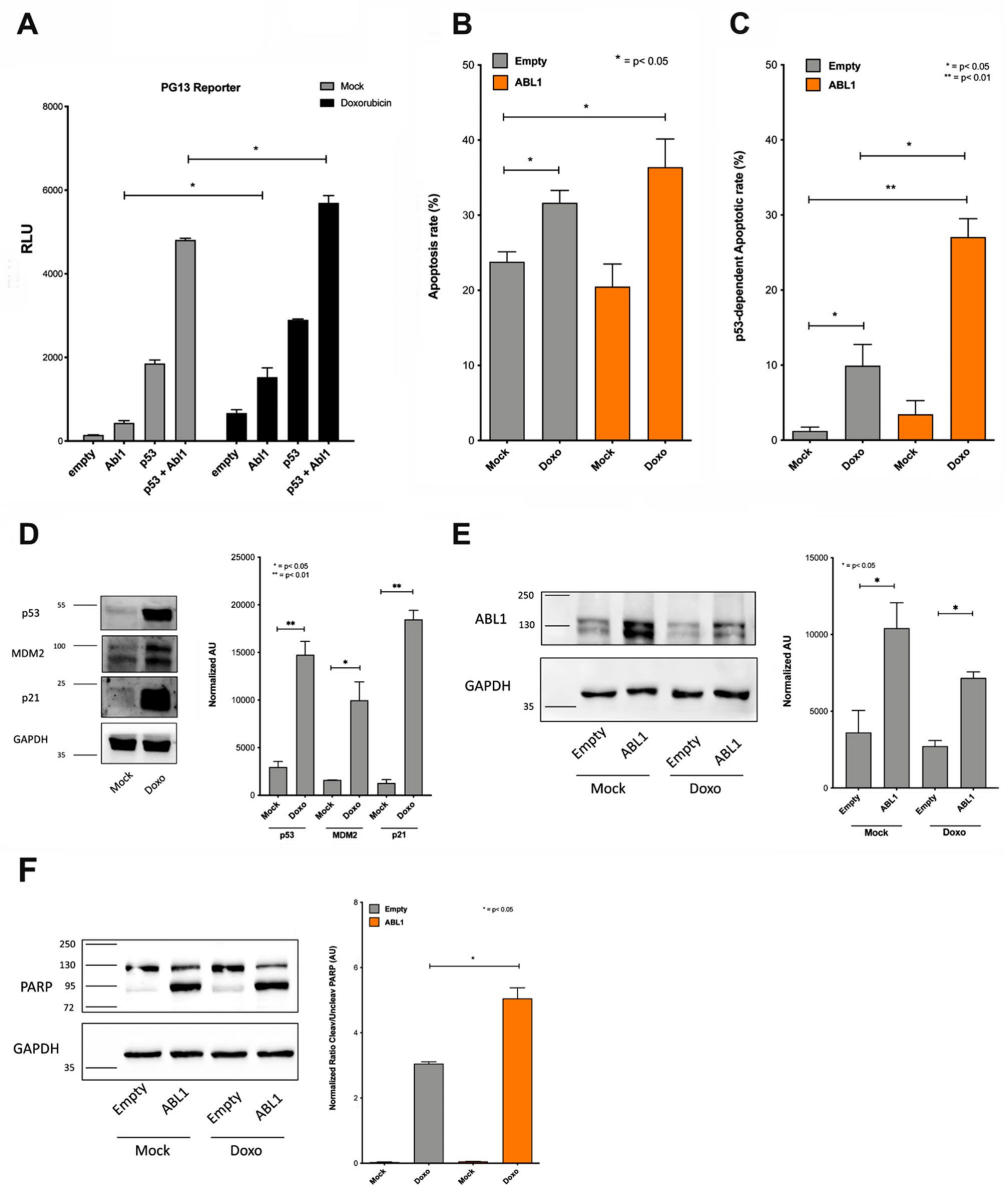
Abl1 stimulates p53-mediated apoptosis. **Panel A**: Human MCF7 cells were transiently transfected with a consensus p53 reporter vector (PG13) along with p53 or/and Abl1 expression vectors. Additionally, cell cultures were treated with 1.5 µM doxorucibin for 16 h. Results are expressed as average relative light units (RLU), normalized to the Renilla control. Abl1 overexpression induced p53-dependent activity on the PG13 reporter. The combined expression of Abl1 and p53 caused a greater than additive induced activation, therefore demonstrating cofactor activity of Abl1 on p53 dependent transcriptional responses. Using the same experimental approach treatment with doxorubicin caused a further significant increase in the reporter assay. Bars plots are averages with standard deviations for 3 independent biological replicates. * = *p* < 0.01 Student t-test. **Panels B-C**: MCF7 vector and short hairpin p53 silenced cells were transiently transfected with an empty vector or an Abl1 expression vector. Forty-eight hours after treatment cells were stained both with FITC-Annexin V and TO-PRO-3 iodide to detect apoptosis (represented by the population of Annexin V positive cells). The involvement of p53 was calculated by subtracting the apoptotic rate obtained with the p53 proficient cell line (MCF7 vector, panel B) from the one stably silenced for p53 (MCF7 shp53, panel C). Doxorubicin treatment alone induced apoptosis that was superseded by Abl1 (panel B). The effects of Abl1 on the apoptosis rate is even more apparent in MCF7 cells silenced for p53 (panel C). Shown are bar graphs of 3 independent biological replicates. * = *p* < 0.05 and ** = *p* < 0.01 Student’s t-test. **Panels D-F**) Examples of the Western blots in MCF7 cells demonstrating the stabilization of p53 protein and the activation of p53 targets (p21 and MDM2) in response to doxorubicin treatment (**D**); overexpression of Abl1 protein following doxorubicin treatment (**E**) and cleavage of PARP-1 by caspases to hallmark apoptosis. Protein molecular weights are indicated according to the ladder. GAPDH was used as housekeeping protein. The blots were quantified with the Image J64 software, and the data are arbitrary units (AU) of the normalized (with GAPDH) signals. In case of PARP-1 (**F**), the histograms represent the ratio of normalized signal for cleaved PARP to total PARP protein. * = *p* < 0.05 and ** = *p* < 0.01 Student’s t-test

Subsequently, we constructed gene reporter assays in MCF7 cells and evaluated Abl1 activity in response to doxorubicin treatment. We performed two sets of experiments that is one in the absence and one in the presence of doxorubicin. Abl1 overexpression induced p53-dependent activity on the PG13 reporter, and doxorubicin treatment caused a further significant increase in the reporter assay (Fig. 9A). Specifically, overexpression of Abl1 alone caused an increase in p53 dependent reporter activity which was superseded by an ectopic expression of p53. The combined expression of both proteins caused a greater than additive induced transactivation therefore demonstrating cofactor activity of Abl1 on p53 dependent transcriptional responses. Using the same experimental approach, treatment with doxorubicin caused a further significant increase in the reporter assay (Fig. 9A).

### Abl1 stimulates p53-dependent induction of apoptosis

Given the pivotal role of p53 in programmed cell death, we evaluated the impact of increased levels of Abl1 on the p53-mediated induction of apoptosis. Since the transfection procedure can be toxic to cells, we adjusted the protocol to reduce the basal level of cell death. To this aim, we transfected MCF7 cells with different transfectant agents (TransIT-LT1 and Lipofectamine 3000) at increasing concentrations (1, 2 and 5 µg) of the Abl1 expression vector (supplementary Figure S6A). Eventually, we selected TransIT-LT1 and 1 µg of plasmid vector as the most suitable experimental condition. Based on optimized experimental conditions, we transfected ABL1 into MCF7 cells which are stably silenced for p53 (MCF7-shp53). We compared the results to equally transfected empty vector control “MCF7-vector” cells, and following doxorubicin treatment, overexpression of ABL1 resulted in an increased apoptotic response in MCF7-vector cells (Fig. 9B and supplementary Figure S6B). To dissect the role of Abl1 in the doxorubicin-mediated p53-dependent induction of apoptosis, we repeated the same experiment in MCF7 cells stably silenced for p53 (Fig. 9C). This allowed us to quantify the contribution of Abl1 on the p53 dependent apoptosis rate which increased significantly (Fig. 9C). Therefore, ectopic Abl1 expression stimulated p53-dependent activation of apoptosis. We also considered expression of the p53 dependent regulation of p21 and its inhibitor MDM2 which were highly induced upon doxorubicin treatment of MCF7 cells (Fig. 9D). Additionally, we show the expression levels of Abl1 following its transfection into MCF7 cells (Fig. 9E).

Lastly, we considered the caspase dependent activation of the apoptotic program and measured the cleavage of the PARP enzyme. We consistently observed significantly increased doxorubicin-dependent PARP cleavage in Abl1 overexpressing MCF7 cells (Fig. 9F), thus supporting its role in the stimulation of p53 dependent apoptosis. Noteworthy, cytochrome c is a critical effector of p53-induced apoptosis, and doxorubicin treatment caused a significant induction of cytochrome c expression especially in T4 animals (supplementary Table S5).

## Discussion

Doxorubicin has been used for decades; yet, its mechanism of pathogenic sequelae remains insufficiently understood. Our study underscores the broad importance of Abl1 in doxorubicin-induced cardiomyopathies and the fine-tuning of checkpoints through modulation of p53/p73 signaling activity.

The cardiotoxicity of doxorubicin is strictly dose-dependent with reductions in heart weight of 30 to 40% while the transcriptomic data appeared well segregated by the different treatments (Fig. 3A). We observed unique gene regulations for each treatment, and the difference in DEGs are testimony to heterogeneity in genomic responses. Histopathology revealed dose-related damage of the heart, i.e. a combination of myocardial atrophy and dilatative cardiomyopathy. We observed vacuolar degenerations of cardiomyocytes and severe damage of the myofibrillar apparatus resulting in contraction bands. Ultimately, the decreased contractibility of the remaining but harmed cardiomyocytes leads to heart failure.

Contraction bands are one of the four defined lesions causing cell death in the heart [76]. Conversely, myofibrillar degeneration, especially at an early stage, is reversible. We consider the proximity of lesions in the myocardial areas close to the heart tip as influenced by the generally increased mechanical stress of this zone, and the genomic study revealed induced expression of genes coding for sarcomeric proteins, e.g. alpha-MHC and troponin T in addition to Mlc-2a (T4). Interestingly, a similar increased Mlc-2a expression was reported for patients with hypertrophic cardiomyopathy [77], but reports on the regulation of alpha-MHC in cardiac hypertrophy are inconsistent [78]. Furthermore, the ultrastructural changes (Fig. 3) agree with findings of an earlier report [79] where changes in adult rat heart tissue were observed as early as 3 days after doxorubicin treatment.

The transcriptomic study is suggestive for cardiomyocyte subpopulations, and the differences in gene expression programs underscore cell death and tissue recovery, as evidenced by the largely non-overlapping gene expression programs and their enriched annotated functions. This raises the possibility for Abl1 to display a broad range of functions on targeted gene promoters of p53-responsive and non-responsive genes in support of programmed cell death but also tissue regeneration. In fact, the functions of p53 appeared ambivalent in the context of doxorubicin-induced cardiotoxicity with cell death dominating an initial/acute phase and protection from tissue damage in the recovery (= adaptive) phase. Obviously, surviving cardiomyocytes are more resilient to the adverse effects of doxorubicin. While the first action is related to the transcriptional modulation of cell cycle and cell death effectors, the later response is related to an impact on mitochondrial genome fidelity, mitochondrial functions and a broad role of p53 as regulator of the cardiac transcriptome [12, 13, 80–82]. In fact, a dual role of p53 in early and late responses has been reported [83] and, generally, the p53 family of transcription factors is regulated through several mechanisms including phosphorylation of MDM2, HIPK2 and p73 [20, 25, 84–86].

So far only a few studies investigated the role of Abl1 in modulating p53 responses, either through its conditional ablation [87] or *de novo* gain of function mutations in patients [88]. Both Abl1 and p53 have been proposed as broad and important players in heart development and cardiomyocyte homeostasis [12, 17] and to play an active role in the context of DNA damage responses [89].

In the following, we describe four major aspects of doxorubicin-induced cardiomyopathies, namely mitochondrial toxicity, programmed cell death, inflammation and toxic cardiomyopathy.

### Doxorubicin-induced mitochondriopathies

The gene enrichment analysis defined mitochondrial biology to be completely affected by doxorubicin treatment, and prominent examples include mitochondrial biogenesis and fission, organization and localization (supplementary Table S7). Although previous studies already highlighted the importance of doxorubicin-induced mitochondrial toxicity, our study underscores the significance of p53 in mitochondriopathies and modulation of p53 by Abl1.

We consider the regulation of Pip4k2c, Ehmt2, Gper1, Dicer 1, Cul4b, Fyco1, Gn3l and TNNT2 in the context of doxorubicin-induced cardiomyopathy as particularly relevant. Pip4k2c codes for a phosphatidylinositol kinase and responds to reactive oxygen species and senescence by positively regulating autophagosome assembly, while inhibition of Pip4k2c and p53 causes synthetic lethality [90]. Likewise, Ehmt2 codes for a histone H3-K9 methyltransferase whose human homolog has been shown to stimulate p53 transcriptional activity [91], and in an epigenetic manner Ehmt2 regulates pro-apoptotic p53 target genes. When overexpressed, Ehmt2 and the related Ehmt1 promote p53-dependent apoptosis [92]. Especially the T1 treatment induced Ehmt2 expression nearly 3-fold. Consistently, we and other investigators evidenced marked ultrastructural changes of harmed cardiomyocytes (Fig. 2), and the genomic data “echoes” mitochondrial toxicity. Conversely, Ehmt2 inhibits cell death of primary aortic vascular smooth muscle cells by suppressing autophagy [93], and research demonstrated miR-207 to repress Ehmt1 and Ehmt2 expression leading to cardiac hypertrophy [94]. Together, we consider upregulation of Ehmt2 as part of a rescue program, and it will be the subject of future studies to investigate the epigenetic regulation of genes following doxorubicin treatment. Indeed, such an “epigenetic conditioning” of doxorubicin responsive genes could play a critical role in delayed congestive heart failures that are seen in patients even after years of doxorubicin treatment [95, 96].

Doxorubicin induces nearly 6-fold Gper1 (G protein-coupled estrogen receptor 1) expression, and the coded protein functions in cardioprotection [97]. Similarly, Dicer 1 is about 4-fold induced, and its deletion in the heart muscle causes dilative cardiomyopathy [98]. Dicer1 activity will grossly affect the processing of miRNAs, and other investigators likewise reported induced Dicer 1 expression upon doxorubicin treatment [99]. Furthermore, a functional interaction between Dicer1 and p53 in the context of skin carcinogenesis has been reported [100].

A further example relates to cullin-4b (Cul4b). This 4-fold induced protein suppresses p53-mediated cellular senescence and responds to reactive oxygen species [101]. Mechanistically, Cul4b mediates p53 protein degradation [102] while Gn3l, i.e. another p53 responsive DEG which is also highly regulated in T4, promotes p53 responses by destabilizing the p53 inhibitor MDM2 [103]. Lastly, Troponin T2 was significantly upregulated, and given that doxorubicin treatment caused a massive loss of heart tissue (see above), it appears that some cardiomyocytes resisted overt toxicity and did not undergo apoptotic cell death but reverted during the prelethal phase. Therefore, about 60% of the cardiomyocytes developed molecular strategies of survival, and the examples given above are testimony to mitochondrial survival signaling. Note, loss of function mutations in the human TNNT2 gene is linked to hypertrophic cardiomyopathy [104].

Apart from its ability to induce gene expression, doxorubin caused repression of mitochondrial genes. For their important interaction with p53, we focus on the regulation of Prkn, Ube2c, Bnip3, Cenpf, Avp and Fgf13.

Prkn/parkin inhibits the mitophagy-promoting function of p53 in mouse heart [105], and its repression supports survival. Conversely, p53 represses the E2 ubiquitin-conjugating enzyme Ube2c [106], and its inhibition sensitizes breast cancer cells to the DNA damaging effects of doxorubicin [107].

Additionally, the mitochondrial BCL2-interacting protein 3 (Bnip3) supports the clearance of abnormal mitochondria and is suppressed by p53 to protect against hypoxia induced cell death [108, 109]. Genetic studies revealed Bnip3 to be a target of the circadian clock master regulator Bmal1, and it functions in mitochondrial fission and mitophagy. Interestingly, BMAL1 knockouts show reduced Bnip3 protein level and impaired cardiomyocyte function [110]. Moreover, the ROS sensitive Hif1α transcription factor represses Bnip3 activity in the context of myocardial injury [111] while depletion of Bnip3 strongly reduced pyroptosis of cardiomyocytes following doxorubicin treatment [112]. Moreover, genetic ablation of Bnip3 in mice reduced doxorubicin-induced damage of mitochondria and heart failure rates [113]. Therefore, we consider its repression as part of a cardiomyocyte rescue program.

Additionally, in T1 and T4, the centromere protein F (Cenpf) is repressed and although its functions are only partly understood, its cardiac-specific deletion resulted in dilated cardiomyopathy, disruption of the microtubule network and aberrant cellular morphology [114]. Repressed Cenpf transcript expression was also observed in doxorubicin-treated iPSC-derived cardiomyocytes [115].

We observed arginine vasopressin repressed in heart tissue of T4 animals. The gene codes for a preprotein that is processed to 3 mature proteins. These signal through distinct G-protein coupled receptor. The V1A receptor causes reversible left ventricular dysfunction through Gαq-mediated cell signaling [116] and exhibits a profibrotic effect in cardiac fibroblasts. Moreover, Avp signaling activates NF-κB [117], and its increased expression has been linked to heart failure [118], specifically in response to doxorubicin treatment [119]. Finally, Fgf13 is a novel regulator of NF-κB, and its expression is upregulated in cardiomyopathy. Upon nuclear translocation, it interacts with p65/relA to potentiate its role in cardiac hypertrophy [120]. The fact that Avp and Fgf13 are repressed indicates that both genes are part of an adaptive response to doxorubicin treatment.

### Programmed cell death

Doxorubicin stimulates different death pathways [10], and our study provides strong evidence for a critical role of p53 in doxorubicin-induced cell death. Apart from its tumor suppressor function, p53 induces cell death especially in response to DNA damage. Based on genomic footprinting studies, we hypothesize Abl1 cofactor activity for p53 target genes, and bioinformatics evidenced enrichment for p53 and Abl1 binding sites in promoters of regulated genes (supplementary Table S1B). Furthermore, we show ectopically expressed Abl1 to stimulate p53-mediated induction of apoptosis. We therefore infer a novel role of Abl1 in mediating p53-dependent programmed cell death. Especially at T1 and T4, we observed a cluster of upregulated genes. We found Foxo3 to be 3-fold upregulated in cardiac tissue of doxorubicin-treated rats, which together with Foxo1 is expressed in developing and adult cardiomyocytes [121]. While Foxo1 knock-out mice are embryonic lethal, mice lacking Foxo3 are viable but display defects, including hypertrophic growth of cardiomyocytes [122]. Foxo3 functions as a transcription factor, and its nuclear translocation is stimulated via phosphorylation on serine 7 by p38-MAPK. In the present study, we observed upregulation of one of the four p38-MAPKs, i.e. MAPK14 in response to doxorubicin treatment [123]. Additionally, Foxo3 activation mediates doxorubicin-induced apoptosis through increased expression of pro-apoptotic genes, such as TRAIL, Bim and FasL [124].

Moreover, trombospondin-1 (Thbs1) was > 2-fold upregulated, and the gene encodes an anti-angiogenesis protein [125]. Tsp1-mediated apoptosis predominantly affects endothelial cells and requires a delicate interplay of the CD36 receptor with caspases 3 and 8 [126]. Additionally, Tsp1 and CD36 receptor signaling activates JNK and p38 kinases, and the two mediators were repeatedly regulated in our study. While stimulation of JNK1 is essential for the Tsp1-mediated apoptosis, its functions are impaired in JNK1^−/−^ mice [127]. Meanwhile, treatment with SB203580, i.e. an inhibitor of p38 kinases, blocks Tsp1-induced apoptosis in vitro and in vivo [125]. Furthermore, Tsp1 is a transcriptional target of p53 [128]. Consistent with our genomic study, we identified a p53 response element in the Thbs1 gene promoter.

Doxorubicin treatment induced > 3-fold the heme binding protein 2 (Hebp2), and this BH3-only protein functions in mitochondrial pathways of apoptosis [129]. Cells exposed to H_2_O_2_ respond with induced Hebp2 expression, and its activation triggers the release of cytochrome c. Conversely, its silencing protects from apoptosis [130].

A further example relates to an upregulation of tumor protein p53-inducible nuclear protein-1 (Tp53inp1). Tp53inp1 is a target of p53 but also of p73 [131], and its overexpression leads to apoptosis. The expression of Tp53inp1 is markedly increased in cells treated with doxorubicin or irradiation, and its ectopic expression potentiated the apoptotic response [132]. Upon its activation, Tp53inp1 localizes into the ProMyelocyticLeukemia-Nuclear Bodies (PML-NBs) in a complex consisting of p53, PML and HIPK2 [133]. Meanwhile, increased expression of Tp53inp1 stimulates enhanced p53 phosphorylation on serine 46 by HIPK2, and this post-translational modification triggers p53-mediated apoptosis [132].

Especially in T2, Hspa1a1 is upregulated, and its functions span from protein folding, degradation, DNA repair and protection from cell death [134]. Overexpression of Hspa1a1 in a knock-in mouse model protected against doxorubicin-induced toxicity [135]. We therefore consider induced Hspa1a1 expression as an adaptive response to the harmful effects of doxorubicin. Besides, genomic footprinting confirmed p53 binding sites in its promoter to suggest a coordinated p53 response in the upregulation of the Hspa1a1.

We observed regulation of genes involved in chromosome segregation and found Ube2c (Ubiquitin-conjugating enzyme E2C) significantly repressed in T1 and T4 regimens. The protein assists in the transition between metaphase to anaphase, and its inhibition sensitizes breast cancer cells to doxorubicin and radiation therapy [107]. Note, p53 represses Ube2c expression while the mutant p53 has the opposite effect on Ube2c gene expression; the results highlight an aberrant gain-of-function characteristics of the mutant protein (R273H, R175H) [106]. Moreover, treatment of various cancer cells with doxorubicin, 5-fluorouracil or etoposide show wild-type p53 to endorse proper spindle assembly checkpoints, while the mutant p53 forces premature anaphase initiation. Key to this effect are NF-Y transcription factors, and NF-Y mediates the opposite regulatory function of wild type and mutant p53 [136].

### Doxorubicin-induced myocarditis

The gene set enrichment analysis revealed NF-κB-dependent signaling in response to doxorubicin treatment, and our findings agree with results reported by other investigators [137, 138]. A recent study demonstrates a novel role for NF-κB during endothelial-to-mesenchymal transition and vascular injury in a rat model of doxorubicin-induced cardiotoxicity [139], and we likewise observed altered vascular endothelial cells following doxorubicin treatment by TEM (Fig. 3). Importantly, the genes involved in the inflammatory response contain enriched Abl1 and p53 binding sites and function as upstream regulators [140, 141] in the doxorubicin-mediated NF-κB activation.

We found the Ikbkg gene (also known as Nemo) to be upregulated; it encodes for the gamma subunit of the IKK kinase complex and catalyzes the phosphorylation of IκBα. This results in the degradation of the main inhibitor of the NF-κB pathway, i.e. IκB, and the increased expression of Ikbkg stimulates Rel members of the NF-κB family. The IKK complex is also important for the phosphorylation of RelA (= p65) at Ser 536, and this post-translational modification is needed for its nuclear localization and activation of NF-κB target genes [142]. Meanwhile, excessive stimulation of IKK signaling causes cardiomyopathies and heart failure in transgenic mice. Conversely, the Ikbkg/NEMO KO mouse model mitigated this phenotype [143].

Moreover, we observed induced cardiac expression of Mapk14. The gene codes for p38 alpha, a serine-threonine kinase known to be highly responsive to genotoxic stress. p38 also activates the p53 tumor suppressor and functions as an upstream regulator of NF-κB. Together, p38 links pro-inflammatory and pro-apoptotic signaling pathways and was shown to be activated by doxorubicin treatment [144]. Upregulation of p38 activity stimulates p300 degradation that is associated with apoptosis in neonatal cardiomyocytes. Conversely, p38 inhibition prevented cell death [145].

Doxorubicin treatment caused upregulation of the tumor necrosis factor alpha-induced protein (Tnfaip3), and its gene promoter analysis revealed enriched Abl1 binding sites. Thus, we obtained evidence for a potential involvement of Abl1 in the upregulation of this gene. Of note, the transcript is highly responsive to TNF and therefore implicated in cytokine-dependent inflammatory and immune responses [146]. Tnfaip3 is a target of the NF-κB pathway and functions in a negative feedback loop to inhibit NF-κB signaling [147]. Tnfaip3 transcription is also increased in doxorubicin-treated breast cancer cell lines [138].

Additionally, doxorubicin augmented expression of the pro-inflammatory molecule Ccl9 (C-C motif chemokine 9), and this cytokine is named macrophage inflammatory protein 1-gamma. It functions in chemotaxis of dendritic cells, osteoclasts and macrophages [148, 149], and a p53-mediated upregulation of Ccl9 in response to doxorubicin and Nutlin-3 in murine adipocytes has been reported [150]. CCR2^+^ macrophages promote the recruitment of monocytes to infarcted myocardial tissue and express higher levels of inflammatory molecules including Ccl9 [151].

Within the group of upregulated genes, Sema3a was one of the most induced ones (FC > 6-fold). Sema3a is secreted into the extracellular microenvironment and functions in autocrine and paracrine circuits [152]. An increased expression of Sema3a in cancer cells inhibits angiogenesis and tumor growth by reducing tumor vasculature [153]. Moreover, inhibition of Sema3a mitigated doxorubicin-mediated podocyte apoptosis [154]. Consistent with our results, Sang and colleagues demonstrated Sema3a to be strongly upregulated in response to doxorubicin treatment in mice [154].

Additionally, complement component C6 is especially induced in T1 and T4 treated animals. The protein functions in the terminal pathway forming the Membrane Attack Complex (MAC) by the assembly of the components C5b, C7, C8 and C9 [155]. The MAC complex, once activated, induces mitochondrial damage and plasma membrane perforation, and the C5b-9 complement proteins (including C6) are upregulated in doxorubicin-induced nephropathy of mice [156].

A further T4 treatment finding relates to an upregulation of IL-6. This cytokine is a potent inducer of the two main inflammatory pathways, i.e. NF-κB and STAT3. Specifically, IL-6 is a direct transcriptional target of NF-κB, and its levels are strongly induced by TNFα [157]. Together with LIF (Leukemia Inhibitory Factor), STAT3 reinforces the pro-inflammatory responses induced by NF-κB, and the IL-6/JAK/STAT3 signaling pathway is hyperactive in inflammatory conditions [158]. STAT3 signaling involves binding to the IL-6R receptor that stimulates the association with GP130 and Jack1,2 tyrosine kinases, which in turn phosphorylate and activate STAT3 [159]. In macrophages, IL-6 is synergistically upregulated by the combined treatment with lipopolysaccharide (LPS) and doxorubicin [160]. Particularly in cardiomyocytes, IL-6 mediates a cardioprotective response that is dependent on an activation of PI3K/AKT and NO [161]. Conversely, the production of IL-6 by cardiac fibroblasts induces myocardial fibrosis through activation of the TGFß pathway [162]. In addition, IL-6 may function as cytoprotective cytokine in cardiomyocytes by stimulating an upregulation of Bcl-xL [163].

While most genes involved in the inflammatory response are upregulated, a few are repressed, especially in T1. Of particular interest is Prkn (Parkin RBR E3 Ubiquitin protein ligase) and Nlrp6 (NLR family pyrin domain-containing 6). As already mentioned, Parkin prevents stress-mediated cell death by ubiquitination of NEMO, which results in an NF-κB mediated upregulation of OPA1, thereby inhibiting apoptosis [164]. Moreover, Prkn interacts with and inhibits the functions of AIF (Apoptosis-Inducing Factor), a mitochondrial protein that translocates to the nucleus to exert its pro-apoptotic functions [165]. In our study, repressed Prkn supported the survival of mitochondria.

Doxorubicin treatment repressed Nlrp6 expression, and the coded protein is part of an inflammasome multi-protein complex. Loss of Nlrp6 results in defective autophagy [166], and the accumulation of ROS-producing mitochondria stimulated programmed cell death [167]. It is of considerable importance that the mitochondrial fission genes GDAP1 and Dnm1l were highly induced, and the findings emphasize mitochondrial biogenesis in surviving cardiomyocytes (supplementary Tables S5&S7). Furthermore, we identified p53 binding sites within the Nlrp6 promoter. This implies a direct role for p53 in Nlrp6 repression.

Doxorubicin treatment induced expression of the G Protein-coupled Estrogen Receptor 1 (GPER1, alias GPR30), and GPER1 augments production of the potent immune suppressor IL-10 [168] and inhibits NF-κB-dependent signaling [169, 170]. We regard the nearly 6-fold GPER1 mRNA upregulation as cardioprotective, given that its activation via G-1 (a well-known agonist for GPER1 receptor) reduced myocardial infarction and fibrosis [171].

Additonally, we observed significant downregulation of interferon-induced protein with tetratricopeptide repeats 3 (Ifit3) in T4 treated animals. Its regulation depends on interferon signaling, and in general, interferon-induced protein with tetratricopeptide repeats (IFIT) play an important role in the defense against viral infections [172]. Although the role of Ifit3 in cardiac biology is uncertain, very recently, it was reported that downregulation of Ifit3 relieved the inflammatory response and myocardial fibrosis of mice with myocardial infarction and improved their cardiac function [173]. Therefore, we regard repressed Ifit3 transcripts as an adaptive response to reduce the inflammatory response induced by doxorubicin treatment. Interestingly, exposure of HeLa cells to doxorubicin caused an increased production and secretion of Ifng and activation of several interferon-stimulated genes [174]. Conversely, IFNγ transgenic mice develop chronic myocarditis leading to severe cardiomyopathy [175], and IFNγ plays a critical role in doxorubicin-induced cardiomyopathy [8].

### Doxorubicin-induced cardiomyopathy

Among highly regulated genes, we noticed increased sarcolipin (SLN) expression from > 4 to > 6-fold in response to T3 and T4 treatments. Sarcolipin influences Ca^2+^ uptake in cardiac muscle by inhibiting SERCA1a and SERCA2a activity, and these ion transporters function as Ca^2+^ pumps of the sarcoplasmic reticulum to facilitate contraction [176]. Conclusive evidence for sarcolipin’s function stems from its cardiac-specific overexpression that causes reduced myocyte contractility and impaired SERCA2a calcium cycling [177]. Consistent with an earlier report [178], the SERCA2 Ca^2+^ ATPase expression was slightly repressed but did not reach statistical significance. Importantly, the zinc finger protein early growth response 1 (EGR1) is a transcriptional repressor of SERCA2, and we observed a significant 3-fold repressed EGR1 expression in cardiac tissue of treated animals. Given its function as a transcriptional repressor, it is not surprising that SERCA2 expression was only mildly changed. Together, we provide evidence for doxorubicin to induce sarcolipin in cardiac tissue and to repress EGR1 with major implications on Ca-homeostasis. A recent opinion paper highlighted the complexity surrounding the question of whether sarcolipin upregulation is beneficial or detrimental to muscle function, and in an effort to regain Ca-homeostasis it was hypothesized that an increased SLN/SERCA ratio promotes Ca signaling and mitochondrial function [179].

As described above, GPER1 is 6-fold induced in response to T1 treatment, and the importance of GPER1 in the Ca^2+^ signaling of the cardiovascular system is the subject of a recent review [180]. Obviously, GPER1 is part of a complex calcium signaling machinery [181], and its upregulation arbitrates cardio-protection following doxorubicin-induced ischemia, oxidative stress and apoptosis [182].

Moreover, a number of kinases were upregulated including fibroblast growth factor receptor 1 (FC ∼ 3-fold), and this protein is essential for cardiomyocyte development [183]. Conversely, the fibroblast growth factor FGF13 was 3-fold repressed at T4 treatment, and this factor is a novel regulator of NF-κB which potentiates cardiac hypertrophy [120]. Additionally, we observed induced expression of NIMA-related kinase 1 at T1, and this kinase signals in response to DNA damage to control DNA repair, mitochondrial activity and cell fate determination [184] while the significant 3-fold upregulation of secretogranin II protects from ischemia-reperfusion injury and cardiomyocyte apoptosis [185]. Furthermore, the 3-fold upregulation of the RNA binding motif protein 4B (RBM4) examplifies the significant genomic responses with its reactivation in heart failure driving the transcriptome into a fetal state [186].

Lastly, we wish to discuss some highly regulated genes in heart development in response to doxorubicin treatment. The T1 treatment caused > 4-fold induced roundabout guidance receptor 1 (Robo1) transcript expression. Although research into the Slit–Robo signaling in heart development is still in its infancy, genetic studies already demonstrated its importance in cardiac cell migration, heart chamber and ventricular septum formation and the development of the pericardium [187]. Furthermore, the T4 treatment elicted induced expression of the Robo ligand Slit1 whereas Slit3 was either mildly repressed or unchanged. Doxorubicin treatment also led to epigenetic modifications, and Robo1 is one of the genes with three CpG islands which were differentially methylated [188]. We also identified sarcoglycan beta as highly significantly repressed to < 20% of controls, and this protein is a component of the dystrophin-glycoprotein complex of the sarcolemma and protects the muscle fibre from damage. Disruption of the heart sarcoglycan complex causes severe cardiomyopathy and muscular dystrophy [189]. Conversely, nephrocystin 1, i.e. a structural protein of primary cilium that functions as sensory organelle, was 6-fold induced especially at T1. Although an existence of primary cilia in cardiomyocytes is controversial, an immunohistochemistry and immunofluorescence microscopy study provided evidence for its existence in the rat heart, and it may contribute to Ca^2+^ homoeostasis [190].

Other genes coding for the myofibrillar apparatus included the myosin light chain 7 which we found increased in expression from initially 2-fold (T1 treatment) to 10-fold in response to T4 treatments. Alike the myosin heavy chain, polypeptide 6 and the titin-cap was nearly 3- and 2-fold induced following T4 and T3 treatments. Intriguingly, the titin cap functions as a molecular spring and is responsible for the elasticity during muscle contraction. It also serves as a molecular scaffold for the actin and myosin components, which polymerize to the thin and thick filaments, respectively of the myofibrillar apparatus. Therefore, their regulation highlights damage to the myofibrillar apparatus.

Another important finding relates to the nearly 6-fold upregulation of myosin binding protein H-like in T4 animals. Very recently, it was reported that myosin-binding protein H-like regulates myosin-binding protein distribution and functions in atrial cardiomyocytes [191]. Given the significant damage of atrial cardiomyocytes following doxorubicin treatment, and the fact that partial and complete loss of myosin binding protein H-like causes cardiac conduction defects and dilated cardiomyopathy [192], we considered its upregulation as an adaptive response to alleviate conduction disturbance following doxorubicin treatment.

Meanwhile, the assembly factor for spindle microtubules (ASPM) was consistently repressed, and its reduction to 20% of controls defines the high-dose treatment regimens. Importantly, microtubules are an integral part of the cytoskeleton and hallmark cellular differentiation [193]; its down-regulation signifies doxorubicin-induced cardiomyopathy. A further example is the highly > 6-fold upregulation of semaphorin 3A especially at T2. There is growing knowledge on the role of semaphorin signaling in cardiovascular development, and SEMA3A is essential for heart rate control [194]. Intriguingly, a recent study highlighted a semaphorin3A-inhibitor to ameliorate doxorubicin-induced podocyte injury/apoptosis in kidney [154]. Besides, dicer 1, i.e. a ribonuclease III endoribonuclease, was nearly 4-fold induced at T1 treatment, and next to its fundamental role in the maturation of miRNAs also functions as a metabolic switch in cardiac mesenchymal stem cells and regulates mitochondrial metabolism to possibly encourage their proliferation and adaptation to adverse conditions [195].

Key markers of congestive heart failure, i.e. ANP and troponin T2 were induced; however, other troponins and tropomyosin did not change in response to doxorubicin treatment, presumably due to differences in cardiomyocyte subpopulations especially the ones which survived toxic injury.

In future studies we will employ single cell RNA sequencing to decipher the genetic program specifically associated with resistance of cardiomyocytes to doxorubicin treatment. Nonetheless, the present study informed on distinct transcriptomic responses, which greatly differed among the various treatments, and the genome wide scans provided evidence for divergent genetic programs. Furthermore, we gained information on adaptive responses, and this includes the significant 3-fold repression of arginine vasopressin. Typically, this peptide hormone is upregulated in heart failure. Reverting neurohormonal imbalance is a hot topic with research focusing on V1a and V2 receptor antagonism [118], and we observed a significantly repressed V1a receptor in cardiac tissue of doxorubicin-treated rats. Additionally, dynamin 1 like protein (dnm1l) is a mitochondrial fission protein and regulator of apoptosis, which we found 5- and 4-fold induced at T2 and T3 treatments but returned to normal at T4. This protein is upregulated in heart ischemia and has become a therapeutic target for cardiac arrest [196]. We also view the 3-fold induced myoglobin expression as an adaptive response to hypoxia. Conversely, the > 6-fold induced elastin microfibril interfacer 1 (emilin-1) highlights a complex interplay with elastin in the extracellular matrix and functions in cell adhesion, migration and proliferation. It is a negative regulator of the TGF-beta signaling with diverse roles in cardiovascular biology, i.e. cardiac repair, remodeling and regeneration but also activates the extrinsic apoptotic pathway [197, 198].

### Study limitations

We wish to highlight the following caveats: First, although indispensible in biomedical research and with important similarities to the human heart, the rodent contractile apparatus differs in regards to heart rate and ventricular action potential duration. Second, the myofilament isoform composition of rat cardiomyocytes, the myofilament Ca2 + sensitivity and calcium homoestasis differ to that of humans. Third, ultrasound imaging of cardiac function would have been a valuable addition to our study, and future works need to evaluate the role of Abl1 at later time points, i.e. after weeks of treatment cessation. Forth, differences in doxorubicin pharmacokinetics, metabolism and therefore species sensitivity are confounding factors that need to be considered. Fifth, we did not perform single cell transcriptomics. Nonetheless, a recent study defined cellular heterogeneity and distinct differences between atrial and ventricular subsets of cardiomyocytes [7]. Based on the present findings, i.e. transcriptomic, histopathology and immunohistochemistry, we obtained clear evidence for cardiomyocyte subpopulations to differ in their resilience and reparative capacity following doxorubicin treatment.

## Conclusions

Abl1 is an important mediator of the genomic responses to doxorubicin treatment. Although Abl1 itself does not function as a transcription factor, this tyrosine kinase operates as a co-activator and modulates transcriptional responses through its interaction with multiple targets, with p53 playing a decisive role in doxorubicin-induced apoptosis. Consistent with its known activity in the elongation phase of gene transcription through tyrosine phosphorylation of RNA Pol II CTD [199, 200], Abl1 could then be proposed to act coordinatively in the transcriptional modulation of doxorubicin-regulated genes. Our study extends the growing list of Abl1 functions that ranges from survival signaling, proliferation and programmed cell death. Hence, fine-tuning and modulation of Abl1 kinase activities may represent a protective mechanism to ameliorate doxorubicin unwanted side effects. Furthermore, given Abl1’s role in cancer, we hypothesize that modulating the Abl1/p53/p73 axis will be of therapeutic benefit not only in alleviating cardiac toxicity but also in regards to disease progression as shown in heavily pretreated gastrointestinal sarcoma tumor patients where low-dose combinations of Imatinib enhanced the anti-tumour activity of doxorubicin [201].

## Electronic supplementary material

Below is the link to the electronic supplementary material.

Supplementary Material 1

## Data Availability

The datasets supporting the conclusions of this article are included within the article and its additional files.

## Abbreviations

ANOVA: Analysis of variance
ChIP: Chromatin immunoprecipitation
CM: Composite module
Cmax: Peak drug concentration
CONS: Consensus
DAPI: 4′,6-diamidino-2-phenylindole
DEG: Differentially expressed gene
DMEM: Dulbecco’s Modified Eagle’s Medium
EGFR: Epidermal growth factor receptor
EMSA: Gel electrophoresis mobility shift assays
FACS: Fluorescence activated cell sorting
FDR: False discovery rate
FITC: Fluorescein isothiocyanate
GAPDH: Glyceraldehyde 3-phosphate dehydrogenase
GO: Gene ontology
HE: Hematoxylin and eosin
HRP: Horseradish peroxidase
IHC: Immunohistochemistry
LFB: Luxol fast blue
LIF: Leukemia inhibitory factor
LPS: Lipopolysaccharide
MAC: Membrane attack complex
MAPK: Mitogen-activated protein kinase
NF-κB: Nuclear factor kappa B
NF-Y: Nuclear factor Y
PALS: Periarteriolar lymphoid sheaths
PARP: Poly ADP-ribose polymerase
PWM: Positional weight matrix
ROS: Reactive oxygen species
SDS-PAGE: Sodium dodecyl sulfate – polyacrylamid gel electrophoresis
TAD: Topologically associating domain
TEM: Transmission electron microscopy
TF: Transcription factor
TNF: Tumor necrosis factor
TSS: Transcription start site
WB: Western blot
Y1H: Yeast one-hybrid
