# Supplementary material for: The Abl1 tyrosine kinase is a key player in doxorubicin-induced cardiomyopathy and its p53/p73 cell death mediated signaling differs in atrial and ventricular cardiomyocytes
Source: J Transl Med. 2024 Sep 16;22:845. doi: 10.1186/s12967-024-05623-8 (PMC11403941; doi:10.1186/s12967-024-05623-8)
Supplement: Supplementary file 1 — Supplementary Material 1 [file 12967_2024_5623_MOESM1_ESM.pdf]

SUPPLEMENTARY FILE

Table of Contents

Supplementary Figures

Supplementary Figure S1. Lung, kidney and liver organ weight changes following doxorubicin treatment.

Supplementary Figure S2. Commonly regulated genes amongst two doxorubicin treatment groups.

Supplementary Figure S3. Electrophoretic mobility shift assays (EMSA) with nuclear protein extracts of control and doxorubicin-treated rat hepatocyte cultures.

Supplementary Figure S4: DNA binding activity of Abl1 on p53 targeted genes in the liver of doxorubicin-treated animals and rat hepatocyte cultures.

Supplementary Figure S5. Abl1 cannot act as a sequence-specific transcription factor in yeast.

Supplementary Figure S6. Abl1 transfection in MCF7 cells and annexin 5 FACS analysis.

Supplementary Tables

Supplementary Table S1A. Significantly enriched Abl1 and p53 transcription factor binding sites in promoters of Doxorubicin regulated genes.

Supplementary Table S1B. Significantly enriched Abl1 and p53 transcription factor binding sites in genes uniquely regulated in T1 - T4 treatments.

Supplementary Table S2. Oligonucleotides used for EMSA.

Supplementary Table S3. Primer for qRT-PCR (human and mouse).

Supplementary Table S4. Primer sequences.
S4A. Abl1 cloning and sequencing primers.
S4B. Primers used for the construction of Abl1 reporter strains.

Supplementary Table S5. Differentially expressed genes in rat heart tissue following doxorubicin treatment (T1-T4).

Supplementary Table S6. Inconsistently regulated DEGs among different doxorubicin treatments (T1-T4).

Supplementary Table S7. Common and specific GO terms for the T1-T4 doxorubicin treatments.

Supplementary Table S8. Independent confirmation of p53/Abl1 target genes.

Supplementary Figures

Supplementary Figure S1. Lung, kidney and liver organ weight changes following doxorubicin treatment.

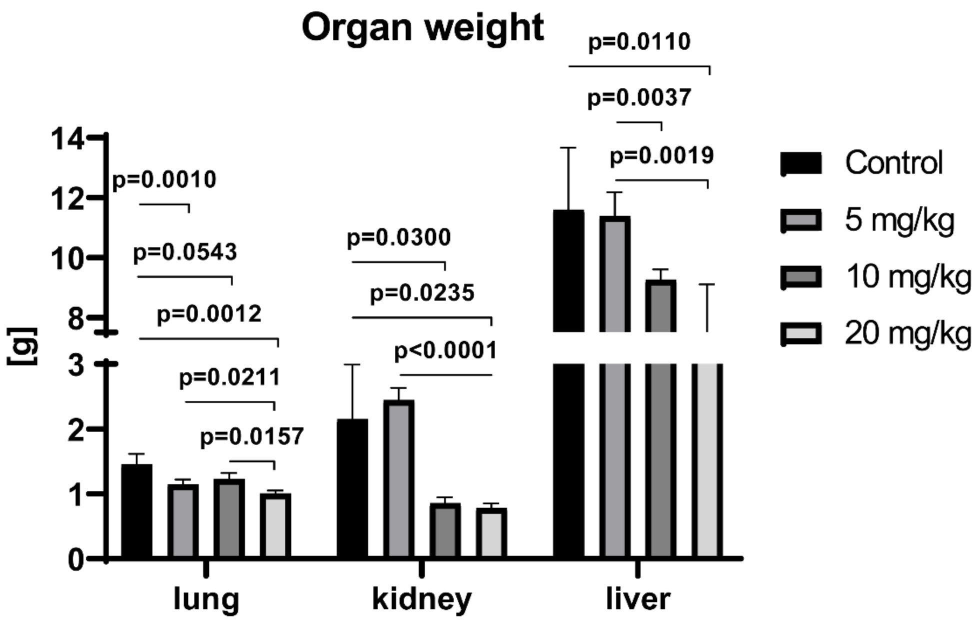

Supplementary Figure S2. Commonly regulated genes amongst two doxorubicin treatment groups.

Data are fold change ± SD. *p<0.05, **p<0.01, ***p<0.001 and ****p<0.0001.

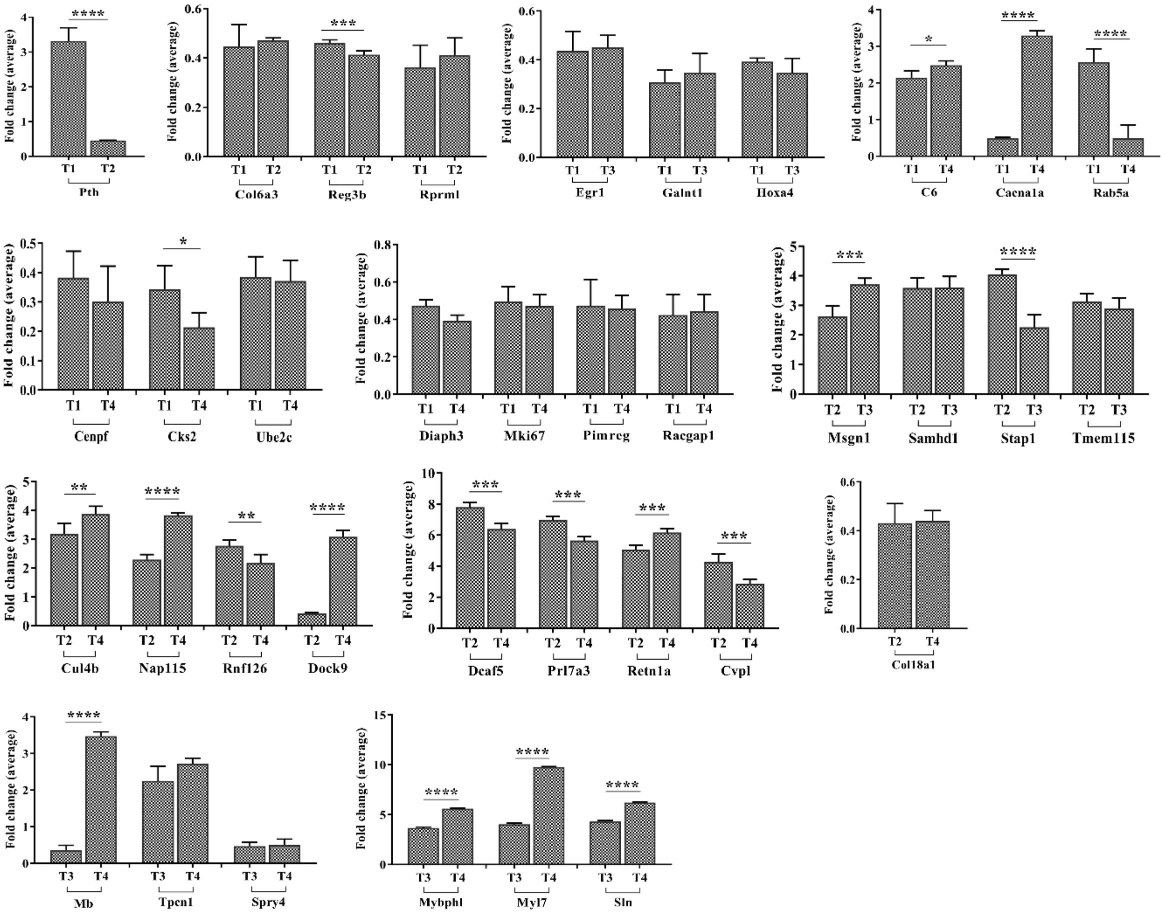

Supplementary Figure S3. Electrophoretic mobility shift assays (EMSA) with nuclear protein extracts of control and doxorubicin-treated rat hepatocyte cultures.

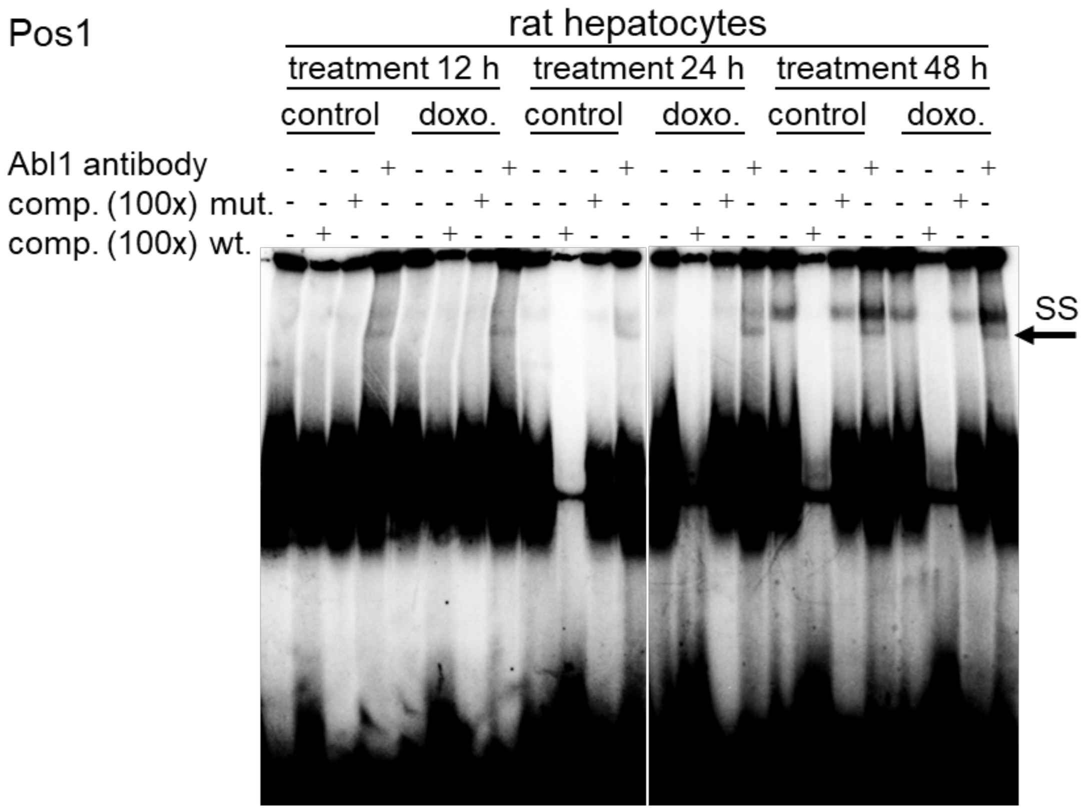

Supplementary Figure S4: DNA binding activity of Abl1 on p53 targeted genes in the liver of doxorubicin-treated animals and rat hepatocyte cultures.

Panel A) EMSA with nuclear protein extracts of rat hepatocyte cultures. We performed gel electrophoresis mobility shift assays with oligoprobes that harbour gene specific promoter binding sites for Abl1. Depicted are autoradiographs of nuclear protein complexes which were isolated from control and doxorubicin-treated rat hepatocyte cultures. The ^{32}P-labelled oligoprobes are specific for Tcpn1, Pla2g2a and Sycp2 and Hopx, and competition assays were done with perfect matched (wt) and mutated probes (mut). Supershifts were carried out with two different antibodies which recognize Abl1 as detailed in the method section. In competition assay with 100-fold excess of the wt-probe, the binding of the nuclear protein is significantly diminshed whereas the mutated probes failed to shift the band. Furthermore, in super-shift assays, the antibody recognized Abl1 protein of the bound nuclear protein complex. As detailed in the results section, variable amounts of the Abl1 protein could be super-shifted.

Panel B) EMSA assays with nuclear protein extracts isolated from control and doxorubicin-treated animals. The band shift assays confirm Abl1 DNA binding. For further details see panel A.

Panel C) Liver organ weights and Western blots of CDK1, Abl1, p53 and p73. When compared to controls, doxorubicin treatment caused a clear dose-related and highly significant reduction in liver weights. The difference in organ weight between treatments is likewise significant. Immunoblots were performed with pooled nucler protein extracts (N=3 animals) of liver tissue from control and doxorubicin-treated animals and rat hepatocyte cultures. CDK1 expression is slightly and dose-related increased. On day 1 at 10 mg/kg Abl1 protein expression is initially decreased but returned to control values subsequently. P53 expression is markedly induced and remained elevated throughout the treatment schedule. Conversely, p73 is slightly but consistently elevated at the different treatment doses and time points.

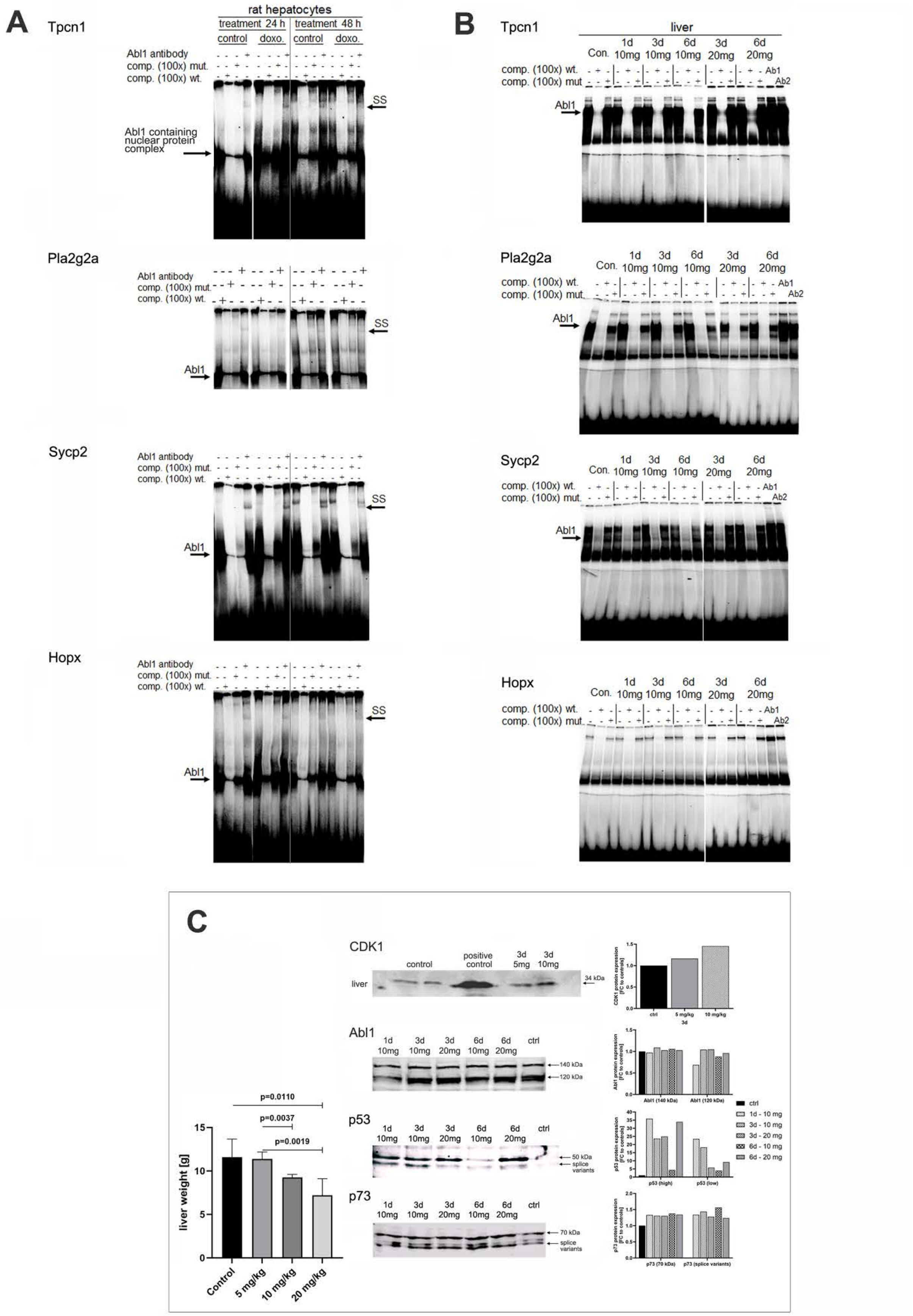
A
Tpcn1
rat hepatocytes
treatment 24 h
treatment 48 h
control
doxo.
Abl1 antibody
comp. (100x) mut.
comp. (100x) wt.
SS
Abl1 containing nuclear protein complex
Pla2g2a
Abl1
Sycp2
Hopx
B
liver
Con.
1d 10mg
3d 10mg
6d 10mg
3d 20mg
6d 20mg
Ab1
Ab2
C
CDK1
control
positive control
3d 5mg
3d 10mg
liver
34 kDa
Abl1
1d 10mg
3d 10mg
3d 20mg
6d 10mg
6d 20mg
ctrl
140 kDa
120 kDa
p53
50 kDa
splice variants
p73
70 kDa
p=0.0110
p=0.0037
p=0.0019
liver weight [g]
Control
5 mg/kg
10 mg/kg
20 mg/kg

Supplementary Figure S5. Abl1 cannot act as a sequence-specific transcription factor in yeast.

Panel A) Western Blot and **Panel B)** immunofluorescence of Abl1 and p53 proteins. Abl1 fused to a yeast-active Transactivation Domain (TAD) and p53 were expressed at high levels (2% galactose); proteins were detected using the same antibody (SantaCruz, sc-126) directed against the TAD. The amount of soluble extract loaded on the gel is indicated. Nuclear staining with DAPI and p53 or Abl1 indirect immunofluorescence is presented as separate images and also overlaid. Cells transformed with an empty vector were used as controls. Images are representative for the cell population. **Panel C)** Yeast reporter strains containing the indicated Abl1 binding sites upstream of the luciferase reporter were tested for transactivation upon induction of Abl1 at moderate (0.016% galactose) and high (2% galactose) levels. Presented are the average light units relative to 1 μg of protein extract (RLU) and the standard deviations of three biological replicates.

Panel D) Yeast reporter strains containing the p53-responsive element PUMA (grey bars) or PUMA RE fused with Abl1 consensus binding site (black bars) upstream of the luciferase reporter were tested for transactivation upon induction of Abl1 at moderate (0.016% galactose) and high (2% galactose) levels. Presented are the average light units relative to 1 μg of protein extract (RLU) and the standard deviations of three biological replicates.

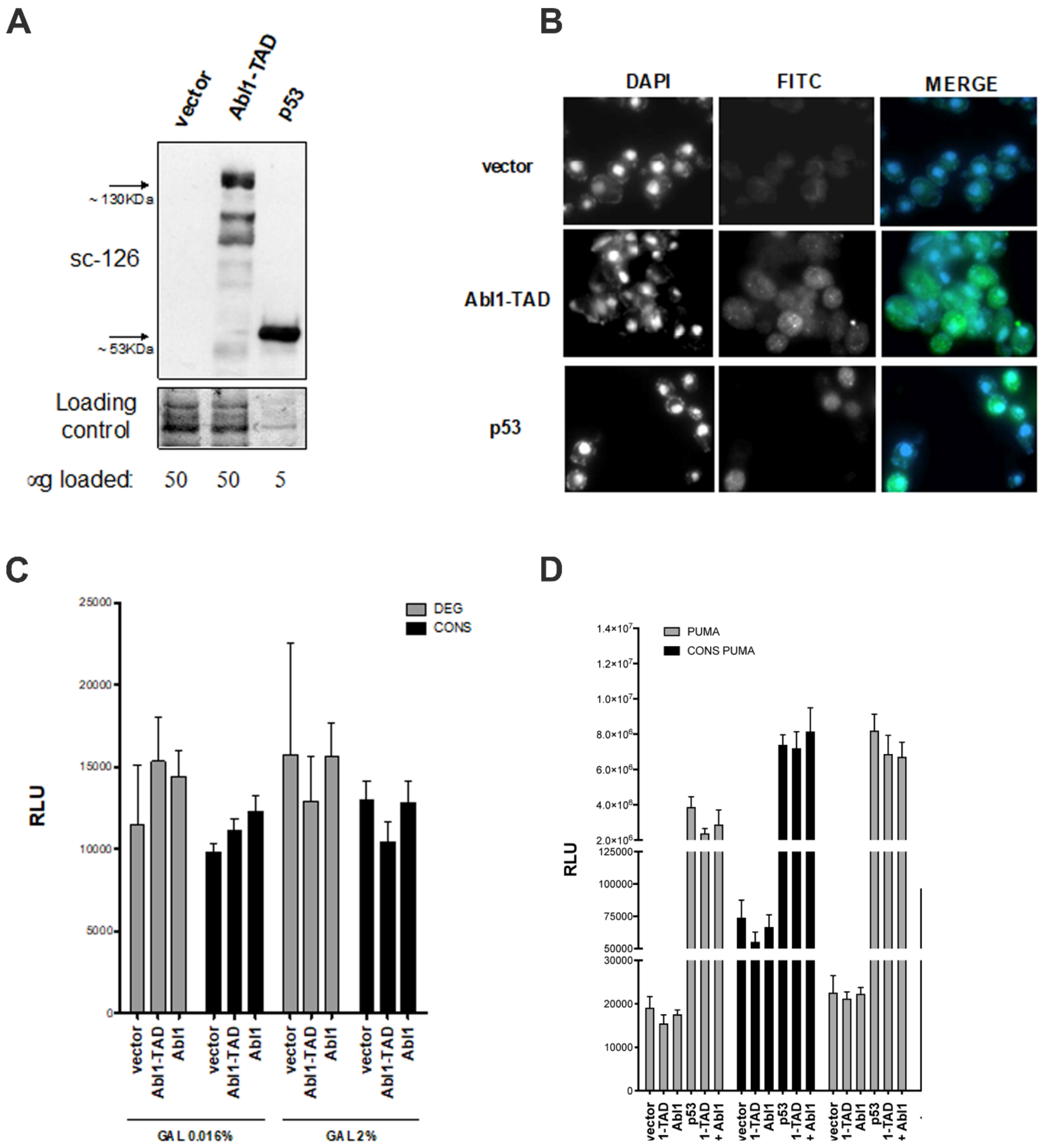
A
vector
Abl1-TAD
p53
~130KDa
sc-126
~53KDa
Loading control
µg loaded: 50 50 5
B
DAPI
FITC
MERGE
vector
Abl1-TAD
p53
C
RLU
DEG
CONS
25000
20000
15000
10000
5000
0
vector
Abl1-TAD
Abl1
GAL 0.016%
GAL 2%
D
PUMA
CONS PUMA
RLU
1.4×10^7
1.2×10^7
1.0×10^7
8.0×10^6
6.0×10^6
4.0×10^6
2.0×10^6
125000
100000
75000
50000
30000
20000
10000
0
vector
1-TAD
Abl1
p53
1-TAD
+ Abl1

Supplementary Figure S6. Abl1 transfection in MCF7 cells and annexin 5 FACS analysis. Panel A) To determine the best experimental set up for analyzing the role of Abl1 in the p53-dependent apoptosis stimulated by doxorubicin, we performed a series of transient transfections in MCF7 vector cells with different transfectant agents (TransIT-LT1 and Lipofectamine 3000) and increasing concentrations of the Abl1 expressing vector (1 and 2 μg) which was followed by western blotting. Cells were harvested 56 hours post-transfection to mimic the experimental conditions used to evaluate the induction of apoptosis. Protein molecular weights are indicated according to the ladder. GAPDH served as reference protein. **Panel B)** An example of FACS scatter plot to evaluate both Annexin V positivity (X axis) and TO-PRO-3 iodide (Y axis): Q1= necrotic cells; Q2 and Q4 = apoptotic cells; Q3 = live cells.

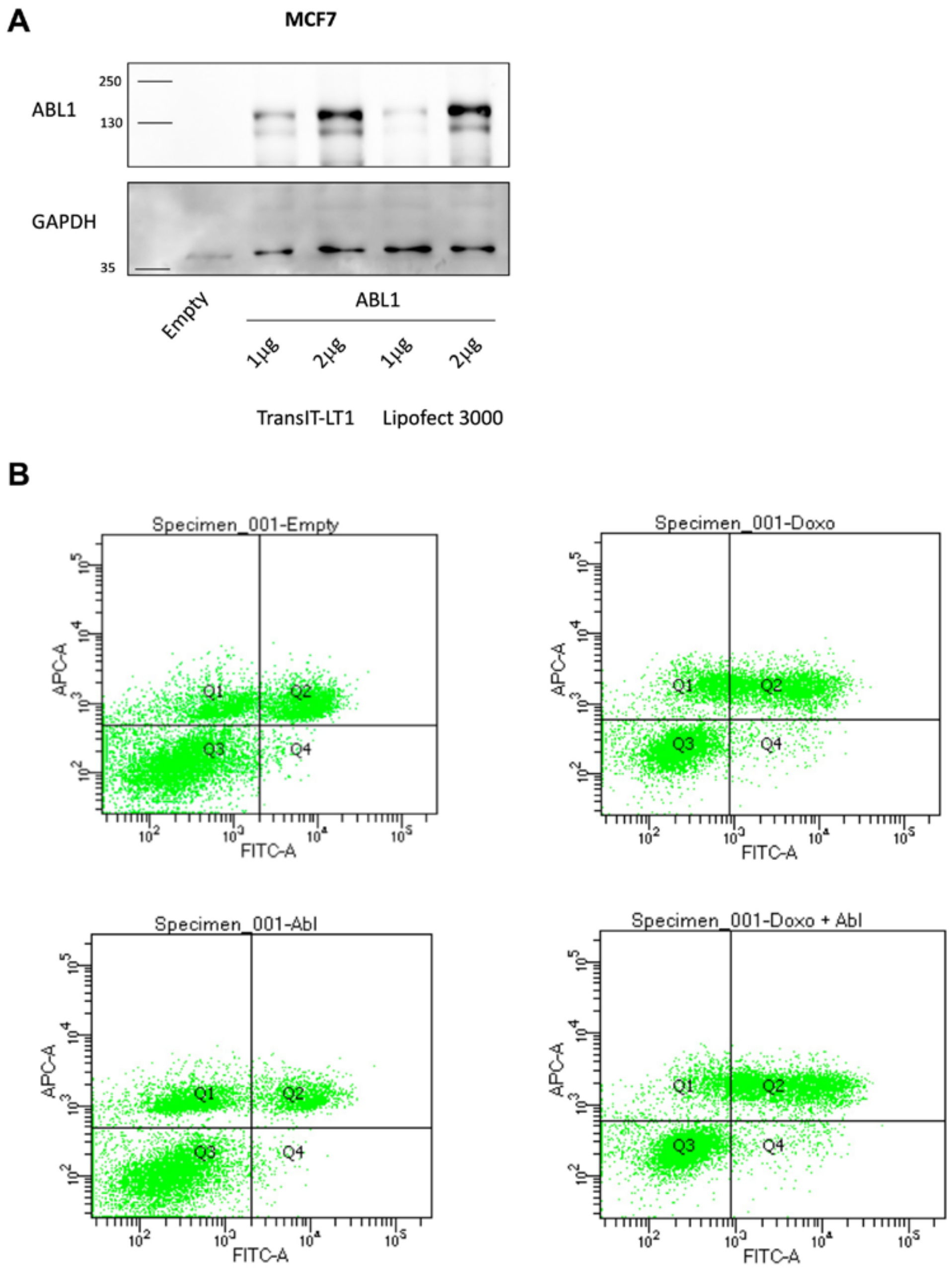

Supplementary Table S1A: Significantly enriched Abl1 and p53 transcription factor binding sites in promoters of Doxorubicin regulated genes.

PWM ID	Treatment	Yes density per 1000bp	No density per 1000bp	Yes-No ratio	P-value
V$ABL1_01	T1	0,140428677	0,104004076	1,350222827	0,013920352
V$TRP53_01	T1	0,103473762	0,023563423	4,391287305	4,20E-05
V$TRP53_02	T1	0,657797487	0,45908049	1,432858728	0,00146328
V$TRP53_01	T2	0,460448642	0,204758024	2,248745292	1,33E-05
V$TRP53_02	T2	1,452184179	0,873471729	1,662542852	3,52E-07
V$TRP53_03	T2	0,306965762	0,146255731	2,098828939	8,35E-04
V$TRP53_01	T4	0,408673895	0,20313296	2,011854178	2,34E-05

Supplementary Table S1B: Significantly enriched Abl1 and p53 transcription factor binding sites in genes uniquely regulated in T1 - T4 treatments.

PWM ID	Treatment	Yes density per 1000bp	No density per 1000bp	Yes-No ratio	P-value
V$ABL1_01	T1	0,293847567	0,142193072	2,066539263	3,43E-04
V$TRP53_01	T1	0,110192837	0,023563423	4,676435832	6,97E-05
V$TP53_03	T2	0,325900515	0,130817626	2,49125843	5,69E-04
V$TRP53_01	T2	0,497427101	0,204758024	2,429341188	3,73E-05
V$TRP53_02	T2	1,595197256	0,897035153	1,778299603	5,17E-07
V$TRP53_03	T2	0,480274443	0,213695874	2,247467081	1,67E-04
V$TRP53_01	T4	0,401416765	0,20313296	1,97612817	4,03E-04

Abl1 and p53 transcription factor binding sites are significantly enriched in DEGs following Doxorubicin treatment.

PWM for Abl1 consensus binding sites

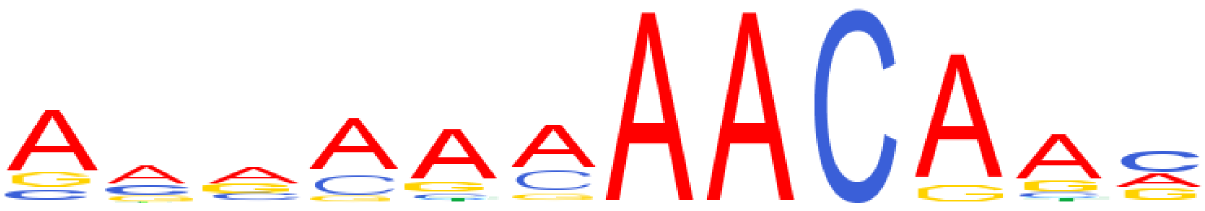

PWM for p53 consensus binding sites

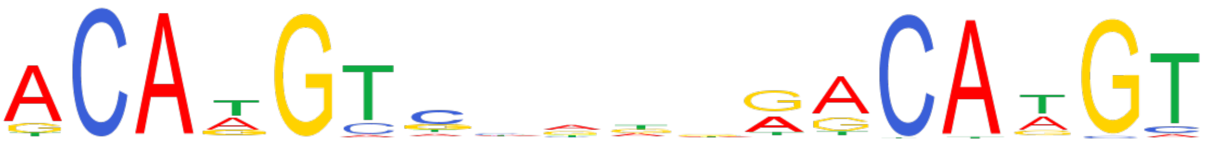

Supplementary Table S2. Oligonucleotides used for EMSA.

Mutations are underlined.

Gene (RefSeq accession number)	Sequence
Pos 1[a]	5’- CACAAACAACAAGACACGA
Pos 1 mutated	5'- CACAGGCGGCAAGACACGA
Neg 1 (Crest NM_138918)	5’- GCCTTTAAAAAACAAAAATGGGT
Mist1 (NM_012863)	5’- AAAAAAACAAAAAAACAAAAGCC
Dnm1l (NM_053655)	5’- TATTTTAAAAACAAAGATTTCAG
Sycp2, site 1 (NM_130735)	5’- TTAAATAAAAACAAACAGTTCAG
Sycp2, site 2 (NM_130735)	5’- AAAAAAAAACAAAACAAAAAGCA
Nppb (NM_031545)	5’- CAGCATAAAACAACACGGAGGGC
Tpcn1 (LOC246215, NM_139332)	5’- AAAACCAAAACAAACAACAAACC
Tpcn1 mutated	5'- AAAACCAAGGCAAGCAGCAAACC
Pla2g2a (NM_031598)	5’- GTTTCAAAAAACAAACAAACAAA
Pla2g2a mutated	5'- GTTTCAAAAGGCAGGCAAACAAA
Add2, site 1 (NM_012491)	5’- ACAAGCAAAAAACAAATAAAAAA
Add2, site 2 (NM_012491)	5’- CCTGTGAAAACAAACAAACAAAC
Giig15b, site 1 (NM_133621)	5’- TACAAAAAACAAAACAAAAACAA
Giig15b, site 1 mutated	5'- TACAAAAGGCAAGGCAAAAACAA
Giig15b, site 2 (NM_133621)	5’- AACAAAAACAAAAACAAACAAAA

[a] Oligonucleotides for positive control, proved Abl1 binding sites published by David-Cordonnier et al. (1998).

Supplementary Table S3. Primer for qRT-PCR.

RT-qPCR Primer (human)	Sequence 5’→3’
B2M-Fw	AGGCTATCCAGCGTACTCCA
B2M-Rv	TGGATGAAACCCAGACACA
GAPDH-Fw	TCCAAAATCAAGTGGGGCGA
GAPDH-Rv	AGTAGAGGCAGGGATGATGT
TPCN1-Fw	CCCTTCATGGACATCCTCCT
TPCN1-Rv	GGAAATTGGCTGTGGTCAGA
HOPX-Fw	GGAGGAGACCCAGAAATGGT
HOPX-Rv	TGGTTAAGCGGAGGAGAGAA
PLA2G2A-Fw	TCATGATCTTTGGCCTACTGC
PLA2G2A-Rv	TGCGGCTTCCTTTCCTGTCG
SYCP2-Fw	TCCACGACCACTGTTTTTGCCCA
SYCP2-Rv	TCACAGGGCATGTCAAACCACGT
DNM1L-Fw	AGACTTTGCTGATGCTTGTGGGCT
DNM1L-Rv	ACCACCTCCAGATGCAACCTTGT
**RT-qPCR Primer (mouse)**	**Sequence 5’→3’**
mGapdh-Fw	GTGCAGTGCCAGCCTCGTCC
mGapdh-Rv	GCACCGGCCTCACCCCATTT
mTpcn1-Fw	GTGACCCGGCTGCTGGACAC
mTpcn1-Rv	GTTCCCGGGCATGCTCCTCG
mHopx-Fw	CCCTAGGGCAGCTCCGGATCT
mHopx-Rv	GTCCGTGCGCGTCTGACTAAG
mPla2g2a-Fw	GGATGCCACAGACCGGTGCT

mPla2g2a-Rv	GCGAAACATTCAGCGGCGGC
mSycp2-Fw	TGCGTCTGTGTCCAGGAGCTCG
mSycp2-Rv	TGGTCGGAGTGTGTCTTGGCGAA
mDnm1l-Fw	TGGAGTTGAAGCAGAAGAATGGGGT
mDnm1l-Rv	GGCTGATCGCCTACAGGTACTTTGG
mGata4-Fw	CACCACGCTGTGGCGTCGTA
mGata4-Rv	TGAATGTCTGGGACATGGAGCTGC

Supplementary Table S4. Primer sequences.

A. Abl1 cloning and sequencing primers.

Name	Sequence (5’ ->3’)
**TAD- Abl1 Nter F**	TGCTCCCCCGTGGCCCCTGCACCAGCAGCTCCTACACCGGCG* ATGTTGGAGATCTGCCTGAAGCTG
**TAD-Abl1 Nter R**	CCGCCGGAGGGGTTTTGGAGTCAG
**cAbl1 Cter F**	GGGCGGCCTGAATGAAGATGAG
**Abl1 Cter CYC R**	ATTACATGATGGTGGCGGCCGCTCTAGAACTAGTGGATCCGTCG AC*CTACCTCTGCACTATGTCACTGAT
**Abl1Nter -F**	TATACCTCTATACTTTAACGTCAAGGGAAAAAACCCCGGTTAAC CTCGAGCC*ATGTTGGAGATCTGCCTGAAGCT
**Abl1 Nter -R**	GGGGACACACCATAGACAGT
**Abl1 P1**	ACAGGATCAACACTGCTTCT
**Abl1P3**	TCATCCACAGAGATCTTGCT
**Abl1P5**	GTCTCCATTGCTCCCTCGAA
**Abl1P7**	CGCCCAACCTGACTCCAAAA
**CYC R**	AGGGACCTAGACTTCAGGTT

Asterisks (*) mark the separation between tails of homology and c-Abl cDNA sequences

B. Primers used for the construction of Abl1 reporter strains.

Name	Sequence (5’ ->3’)
**CONS**	GCGGAATTGACTTTTTCTTGAATAATACAT*AAAAAACAACAAGT TGTTGTTTTTT*GCAGATCCGCCAGGCGTGTATATAGCGTGG
**DEG**	GCGGAATTGACTTTTTCTTGAATAATACAT*CACAACAAAGAGCA CAACAAAGA*GCAGATCCGCCAGGCGTGTATATAGCGTGG
**CONS-PUMA F**	GCGGAATTGACTTTTTCTTGAATAATACAT*AAAAAACAACAAGT TGTTGTTTTTT*CCGCTGCAAGTC
**CONS-PUMA R**	CCACGCTATATACACGCCTGGCGGATCTGC*GGACAAGTCAGGAC TTGCAGCGG*AAAAAACAACAA

Asterisks (*) mark the separation between tails of homology and consensus sequences for Abl1 or p53

Supplementary Table S5. Differentially expressed genes in rat heart tissue following doxorubicin treatment (T1).

Sequence ID	Sequence Code	Gene symbol	Gene description	Fold Change	P-value	Fold change±SD
35180	AA963765	Ogn	osteoglycin	-3,66	0	-3.66±0.06
24196	AW528001	Aspm	abnormal spindle microtubule assembly	-3,29	0	-3.29±0.06
35974	BQ204201	Cnksr1	connector enhancer of kinase suppressor of Ras 1	-3,25	2,48E-43	-3.25±0.07
25473	AI045810	Galnt1	polypeptide N-acetylgalactosaminyltransferase 1	-3,25	0	-3.25±0.05
32989	NM_134375	Nlrp6	NLR family, pyrin domain containing 6	-3,01	0,01	-3.01±0.41
33144	AW525201	Cks2	CDC28 protein kinase regulatory subunit 2	-2,91	5,60E-33	-2.91±0.08
24442	BQ210664	Cilp	cartilage intermediate layer protein	-2,83	0	-2.83±0.06
27657	CB546143	Prkn	parkin RBR E3 ubiquitin protein ligase	-2,78	1,49E-04	-2.78±0.26
28628	BQ209668	Rprml	reprimo-like	-2,76	1,84E-28	-2.76±0.09
30822	AI044806	Ccnb2	cyclin B2	-2,71	2,43E-18	-2.71±0.11
27031	CB606334	Dpt	dermatopontin	-2,71	0	-2.71±0.31
35087	AA901174	Ptpn11	protein tyrosine phosphatase, non-receptor type 11	-2,66	0	-2.66±0.35
35431	NM_012543	Dbp	D-box binding PAR bZIP transcription factor	-2,62	2,88E-42	-2.62±0.07
18399	BF388623	Cenpf	centromere protein F	-2,61	5,23E-31	-2.61±0.09
34148	AI102920	Ube2c	ubiquitin-conjugating enzyme E2C	-2,6	4,76E-44	-2.6±0.07
35015	L03557.1	Hoxa4	homeo box A4	-2,55	4,43E-11	-2.55±0.15
19413	AW524529	Slc6a4	solute carrier family 6 member 4	-2,55	1,67E-25	-2.55±0.1
27348	CB544699	Nsun6	NOP2/Sun RNA methyltransferase family member 6	-2,54	5,45E-07	-2.54±0.2
30395	BF563897	Cytip	cytohesin 1 interacting protein	-2,53	3,93E-06	-2.53±0.22
27954	AJ299016.1	Ntrk2	neurotrophic receptor tyrosine kinase 2	-2,41	1,07E-06	-2.41±0.2
32261	CB545620	Kpna4	karyopherin subunit alpha 4	-2,39	0,01	-2.39±0.4
35416	NM_022211	Fgf5	fibroblast growth factor 5	-2,38	0,03	-2.38±0.46
32559	BF281709	Mob1b	MOB kinase activator 1B	-2,37	4,36E-08	-2.37±0.18
22823	NM_031545	Nppb	natriuretic peptide B	-2,37	0	-2.37±0.04
33679	BQ201398	Racgap1	Rac GTPase-activating protein 1	-2,36	6,46E-19	-2.36±0.11
33396	NM_012551	Egr1	early growth response 1	-2,29	7,43E-34	-2.29±0.08
31595	CB545100	Selenoi	selenoprotein I	-2,29	7,75E-04	-2.29±0.3
28508	NM_022297	Ddah1	dimethylarginine dimethylaminohydrolase 1	-2,28	2,15E-19	-2.28±0.11
33313	BG375279	Pinlyp	phospholipase A2 inhibitor and LY6/PLAUR domain containing	-2,26	1,54E-08	-2.26±0.18
28272	BM387852	Col6a3	collagen type VI alpha 3 chain	-2,24	6,11E-26	-2.24±0.09
34430	NM_033485	Pawr	pro-apoptotic WT1 regulator	-2,24	1,85E-04	-2.24±0.27
34111	BE098732	Uhrf1	ubiquitin-like with PHD and ring finger domains 1	-2,24	1,20E-09	-2.24±0.16
33964	BQ782027	Ska1	spindle and kinetochore associated complex subunit 1	-2,21	8,39E-08	-2.21±0.19
18928	BE108837	Cenpe	centromere protein E	-2,2	4,55E-14	-2.2±0.13
25391	AW251274	Nit2	nitrilase family, member 2	-2,18	2,55E-42	-2.18±0.07
32839	CB545206	Pclo	piccolo (presynaptic cytomatrix protein)	-2,18	5,67E-05	-2.18±0.25
34132	NM_130819	Dhrs9	dehydrogenase/reductase 9	-2,17	0	-2.17±0.32
32179	NM_053289	Reg3b	regenerating family member 3 beta	-2,17	8,13E-14	-2.17±0.13
35191	CB545754	Slc35a3	solute carrier family 35 member A3	-2,17	8,20E-07	-2.17±0.2
32848	CB548245	Sall1	spalt-like transcription factor 1	-2,15	0,01	-2.15±0.41
29007	NM_053356	Col1a2	collagen type I alpha 2 chain	-2,14	5,38E-30	-2.14±0.09
33215	CB545471	Cd2ap	CD2-associated protein	-2,13	0	-2.13±0.33
32960	CB545410	Ect2	Epithelial cell transforming 2	-2,12	6,09E-05	-2.12±0.25
25078	BF398599	Diaph3	diaphanous-related formin 3	-2,11	0	-2.11±0.31
25372	AI712694	Pimreg	PICALM interacting mitotic regulator	-2,11	1,79E-13	-2.11±0.14
34236	AI408045	Prim2	DNA primase subunit 2	-2,08	4,69E-16	-2.08±0.12
27276	BE113362	Cdkn3	cyclin dependent kinase inhibitor 3	-2,07	5,36E-22	-2.07±0.1
27293	M58040.1	Tfrc	transferrin receptor	-2,07	2,08E-14	-2.07±0.13
33522	CB544619	Arhgef7	Rho guanine nucleotide exchange factor 7	-2,06	0,04	-2.06±0.18
34606	NM_031029	Gabrp	gamma-aminobutyric acid type A receptor pi subunit	-2,06	0,01	-2.06±0.37
34122	NM_012918	Cacna1a	Calcium channel alpha 1A	-2,02	1,80E-07	-2.02±0.02
32680	BQ196689	Mki67	marker of proliferation Ki-67	-2,02	1,27E-33	-2.02±0.08
24760	NM_031044	Hnmt	histamine N-methyltransferase	-2,01	0	-2.01±0.34
35957	BF557435	Papln	papilin, proteoglycan-like sulfated glycoprotein	-2,01	0	-2.01±0.33
28164	AI031053	Pdk4	pyruvate dehydrogenase kinase 4	2,02	2,75E-13	2.02±0.14
18969	NM_080480	Pip4k2c	phosphatidylinositol-5-phosphate 4-kinase type 2 gamma	2,06	0,01	2.06±0.38
18117	NM_053656	P2rx2	purinergic receptor P2X 2	2,07	0,02	2.07±0.43
23836	BF523054	Ddi2	DNA-damage inducible protein 2	2,09	6,43E-07	2.09±0.2
22499	BE119668	Dnajb7	heat shock protein family (Hsp40) member B7	2,12	0	2.12±0.34
28823	D00688.1	Maoa	monoamine oxidase A	2,12	9,09E-26	2.12±0.1
18273	CB545304	Sorcs2	sortilin-related VPS10 domain containing receptor 2	2,13	0,01	2.13±0.38
23971	AI045144	C6	complement C6	2,14	0	2.14±0.19
19665	CB548210	Mapk14	mitogen activated protein kinase 14	2,18	0,03	2.18±0.47
18390	BE099443	Dner	delta/notch-like EGF repeat containing	2,19	0	2.19±0.35
28886	NM_031598	Pla2g2a	Phospholipase A2 group IIA	2,2	4,12E-41	2.2±0.07
19046	CB545696	Usp1	ubiquitin specific peptidase 1	2,26	0,04	2.26±0.31
22741	CB545722	Dpp10	dipeptidylpeptidase 10	2,3	0,01	2.3±0.38
19463	CB547540	Hars2	histidyl-tRNA synthetase 2, mitochondrial	2,3	0,01	2.3±0.41
18435	CB545807	Slc11a2	solute carrier family 11 member 2	2,3	0,03	2.3±0.45
19600	NM_057206	Pnliprp2	pancreatic lipase related protein 2	2,31	0,03	2.31±0.46
19057	CB545121	Slc4a1	solute carrier family 4 member 1	2,31	0	2.31±0.34
19188	CB546197	Ddhd1	DDHD domain containing 1	2,33	0,01	2.33±0.4
19968	CB606240	Mthfd2	methylenetetrahydrofolate dehydrogenase (NADP+ dependent) 2	2,36	0	2.36±0.34
35818	BF558909	Itih2	inter-alpha-trypsin inhibitor heavy chain 2	2,37	0	2.37±0.35
22029	CB546760	Fam98a	family with sequence similarity 98, member A	2,39	0,03	2.39±0.45
18805	CB547343	Tnfaip3	TNF alpha induced protein 3	2,41	0,02	2.41±0.41
36193	BM387978	Ikbkg	inhibitor of kappa light polypeptide gene enhancer in B-cells, kinase gamma	2,42	7,40E-07	2.42±0.2
18505	CB545997	Sf3b1	splicing factor 3b, subunit 1	2,42	2,33E-04	2.42±0.27
19612	BF408434	Ehmt2	euchromatic histone lysine methyltransferase 2	2,45	0,01	2.45±0.37
18281	AW917994	C-myb	Myb proto-oncogene protein	2,46	9,30E-04	2.46±0.3
19206	NM_031085	Prkch	protein kinase C	2,48	0,01	2.48±0.39
18744	BF410803	Bpifb1	BPI fold containing family B, member 1	2,49	0	2.49±0.34
26876	CB547449	Ccl9	C-C motif chemokine ligand 9	2,5	0,02	2.5±0.43
23660	M18331.1	Prkce	protein kinase C, epsilon	2,5	0,03	2.5±0.46

23949	AW142399	Nek1	NIMA-related kinase 1	2,53	0	2.53±0.32
19675	BG664942	Herc4	HECT and RLD domain containing E3 ubiquitin protein ligase 4	2,54	0,03	2.54±0.47
18887	NM_022692	Rab5a	RAB5A, member RAS oncogene family	2,57	0,01	2.57±0.36
21669	CB546533	Herc2	HECT and RLD domain containing E3 ubiquitin protein ligase 2	2,58	0,03	2.58±0.45
18723	AI711152	Lingo1	leucine rich repeat and Ig domain containing 1	2,61	0,04	2.61±0.48
33560	NM_019290	Btg3	BTG anti-proliferation factor 3	2,64	0,03	2.64±0.47
29051	CB548073	Kmt2d	lysine methyltransferase 2D	2,66	0,01	2.66±0.39
19223	NM_017139	Penk	proenkephalin	2,69	3,71E-35	2.69±0.08
18810	CB547351	Mtrr	5-methyltetrahydrofolate-homocysteine methyltransferase reductase	2,75	6,82E-06	2.75±0.22
19599	AW144455	Rbm4b	RNA binding motif protein 4B	2,75	3,12E-04	2.75±0.28
18724	BG381331	Igsf21	immunoglobin superfamily, member 21	2,78	1,25E-10	2.78±0.16
19739	NM_030865	Myoc	myocilin	2,82	2,09E-06	2.82±0.21
18119	AI233699	Etv1	ets variant 1	2,83	0	2.83±0.32
19210	AJ312745.1	Fgfr1	Fibroblast growth factor receptor 1	2,86	5,13E-04	2.86±0.29
19962	AI555697	Oxa1l	mitochondrial inner membrane protein	2,9	0,01	2.9±0.4
17738	CB546158	Slf1	SMC5-SMC6 complex localization factor 1	2,91	6,74E-11	2.91±0.15
19194	AI407434	Plxdc2	plexin domain containing 2	2,93	0	2.93±0.33
22782	BF558277	Bloc1s3	biogenesis of lysosomal organelles complex-1, subunit 3	2,97	0,04	2.97±0.36
17959	CB544325	Gan	gigaxonin	3,08	0,04	3.08±0.43
19609	CB545922	Usp53	ubiquitin specific peptidase 53	3,08	0	3.08±0.31
18437	CB544702	Nfrkb	nuclear factor related to kappa B binding protein	3,1	2,45E-04	3.1±0.27
18037	NM_012556	Fabp1	fatty acid binding protein 1	3,26	3,87E-06	3.26±0.22
25114	BF547197	Smarcd2	SWI/SNF related, matrix associated, actin dependent regulator of chromatin, subfamily d, membe	3,27	8,02E-05	3.27±0.25
17547	CB606458	Pth	parathyroid hormone	3,32	0,01	3.32±0.38
18722	BF418352	Mzt2b	mitotic spindle organizing protein 2B	3,38	0,04	3.38±0.42
18738	CB547518	Cdh11	cadherin 11	3,42	8,63E-05	3.42±0.25
18880	CB547355	Dicer1	dicer 1 ribonuclease III	3,6	0,03	3.6±0.47
18311	NM_013012	Prkg2	protein kinase, cGMP-dependent, type II	3,62	0,01	3.62±0.38
18114	NM_031745	Clip1	CAP-GLY domain containing linker protein 1	3,92	1,54E-05	3.92±0.23
19449	NM_022188	Robo1	roundabout guidance receptor 1	4,2	0	4.2±0.32
17745	CB547120	Ascc3	activating signal cointegrator 1 complex subunit 3	4,25	0,01	4.25±0.38
18285	CB544302	Gdap1	ganglioside-induced differentiation-associated-protein 1	5,31	1,22E-07	5.31±0.19
19822	CB545711	Nphp1	nephrocystin 1	5,66	0,01	5.66±0.36
19603	NM_133573	Gper1	G protein-coupled estrogen receptor 1	5,85	3,24E-11	5.85±0.15
17747	BE108409	Camk4	calcium/calmodulin-dependent protein kinase IV	5,93	0,02	5.93±0.42
18799	NM_130820	Pnma1	paraneoplastic Ma antigen 1	6,13	1,87E-12	6.13±0.14
19446	CB545546	Aif1l	allograft inflammatory factor 1-like	6,18	3,76E-09	6.18±0.17
17641	BM391389	Emilin1	elastin microfibril interfacer 1	6,28	6,50E-11	6.28±0.15
19041	AW523329	Ift27	intraflagellar transport 27	6,31	2,38E-06	6.31±0.21
18817	NM_031038	Gnrhr	gonadotropin releasing hormone receptor	6,49	0	6.49±0.36
19132	AI111635	Dcaf17	DDB1 and CUL4 associated factor 17	7,59	1,12E-07	7.59±0.19
19958	BF393903	Cnnm1	cyclin and CBS domain divalent metal cation transport mediator 1	8,98	9,05E-04	8.98±0.3
19356	NM_130735	Sycp2	synaptonemal complex protein 2	11,4	1,62E-12	11.4±0.13
18613	NM_019207	Neurog1	neurogenin 1	18,54	5,93E-17	18.54±0.12
20379	BF550270	Zfp746	zinc finger protein 746	20,29	3,82E-04	20.29±0.28

Supplementary Table S5. Differentially expressed genes in rat heart tissue following doxorubicin treatment (T2).

Sequence ID	Sequence Code	Gene symbol	Gene description	Fold Change	P-value	Fold change±SD
22389	AW522900	Glrb	glycine receptor, beta	-4,2	0,02	-4.2±0.43
31849	AW528825	Kcna6	potassium voltage-gated channel subfamily A member 6	-3,69	5,31E-07	-3.69±0.2
25009	CB547439	Nav1	neuron navigator 1	-3,58	7,32E-04	-3.58±0.3
21931	AW521816	Pop5	POP5 homolog, ribonuclease P/MRP subunit	-3,08	6,29E-24	-3.08±0.1
21100	AA901299	Hoxa3	homeobox A3	-2,93	0	-2.93±0.33
24442	BQ210664	Cilp	cartilage intermediate layer protein	-2,71	3,18E-12	-2.71±0.14
32151	AI712699	Rps4y2	ribosomal protein S4, Y-linked 2	-2,69	3,42E-41	-2.69±0.07
26408	BF549671	Cinp	cyclin-dependent kinase 2-interacting protein	-2,59	2,19E-04	-2.59±0.27
35180	AA963765	Ogn	osteoglycin	-2,58	1,99E-25	-2.58±0.1
26343	AF268593.1	Itga2	integrin subunit alpha 2	-2,56	0	-2.56±0.35
25183	AI412209	Kcnq2	potassium voltage-gated channel subfamily Q member 2	-2,53	0,05	-2.53±0.51
27077	NM_134379	Ust4r	integral membrane transport protein UST4r	-2,51	0,02	-2.51±0.42
28628	BQ209668	Rprml	reprimo-like	-2,43	0	-2.43±0.07
32179	NM_053289	Reg3b	regenerating family member 3 beta	-2,42	8,07E-10	-2.42±0.16
25716	AA957461	Dock9	dedicator of cytokinesis 9	-2,41	0,02	-2.41±0.43
32082	AI030681	Ikbke	inhibitor of kappa light polypeptide gene enhancer in B-cells, kinase epsilon	-2,38	0,03	-2.38±0.45
26401	CB544950	Camta2	calmodulin binding transcription activator 2	-2,33	0,03	-2.33±0.45
25878	AJ001290.1	Slc5a11	solute carrier family 5 member 11	-2,33	0,01	-2.33±0.41
18017	AA818342	Col8a1	collagen type VIII alpha 1 chain	-2,32	9,41E-39	-2.32±0.08
18009	BM385216	Prdm1	PR/SET domain 1	-2,32	3,45E-06	-2.32±0.22
29007	NM_053356	Col1a2	collagen type I alpha 2 chain	-2,3	0	-2.3±0.06
27954	AJ299016.1	Src	SRC proto-oncogene, non-receptor tyrosine kinase	-2,28	4,46E-08	-2.28±0.18
26692	BF563757	Tex35	testis expressed 35	-2,22	0,01	-2.22±0.4
32770	BF419904	Pth	parathyroid hormone	-2,2	2,39E-18	-2.2±0.11
26383	NM_138833	Snrk	SNF related kinase	-2,2	2,40E-04	-2.2±0.27
25873	NM_153621	Dab1	DAB1, reelin adaptor protein	-2,19	0	-2.19±0.34
31880	BU759478	Adamts1	ADAM metallopeptidase with thrombospondin type 1 motif, 1	-2,14	1,66E-26	-2.14±0.09
25575	NM_013008	Pou1f1	POU class 1 homeobox 1	-2,14	0,05	-2.14±0.51
28272	BM387852	Col6a3	collagen type VI alpha 3 chain	-2,12	9,96E-22	-2.12±0.1
24196	AW528001	Aspm	abnormal spindle microtubule assembly	-2,09	5,47E-05	-2.09±0.25
25839	BQ211783	Col5a1	collagen type V alpha 1 chain	-2,06	1,85E-08	-2.06±0.18
31161	BQ198941	Col6a2	collagen, type VI, alpha 2	-2,06	3,03E-21	-2.06±0.11
23104	CB544281	Nudt13	nudix hydrolase 13	-2,04	0,03	-2.04±0.47
31823	BG378435	Ptrh1	peptidyl-tRNA hydrolase 1 homolog	-2,03	3,29E-09	-2.03±0.17
32081	NM_138853	Ubxn11	UBX domain protein 11	-2,03	0,01	-2.03±0.36
24862	NM_031825	Fbn1	fibrillin 1	-2,02	1,74E-04	-2.02±0.27
26572	NM_031557	Ptgis	prostaglandin I2 synthase	-2,01	1,47E-06	-2.01±0.21
34227	BF413193	Fbxo2	F-box protein 2	2	0	2±0.32
32855	CB606280	Msl2	male-specific lethal 2 homolog	2,02	0,01	2.02±3.72
36103	CB546267	Baz2a	bromodomain adjacent to zinc finger domain, 2A	2,03	0,05	2.03±0.5
35982	CB545156	Serpinb1a	serpin family B member 1A	2,04	0	2.04±0.43
19144	NM_031971	Hspa1a	heat shock protein family A (Hsp70) member 1A	2,05	1,64E-36	2.05±0.08
34787	BF545451	Ppl	periplakin	2,07	0,02	2.07±0.46
35736	AF050660.1	Ania8	activity and neurotransmitter-induced early gene 8	2,21	0,02	2.21±0.41
16668	AA946371	Tp53inp1	tumor protein p53 inducible nuclear protein 1	2,21	0	2.21±0.06
22801	BF562863	Nap1l5	nucleosome assembly protein 1-like 5	2,29	6,28E-08	2.29±0.18
36029	NM_020102	Mos	MOS proto-oncogene, serine/threonine kinase	2,33	0	2.33±0.32
35703	CB545552	Ldb1	LIM domain binding 1	2,34	4,17E-06	2.34±0.22
28164	AI031053	Pdk4	pyruvate dehydrogenase kinase	2,39	3,38E-26	2.39±0.09
36095	NM_138912	Ppp1r3b	protein phosphatase 1, regulatory subunit 3B	2,39	7,42E-04	2.39±0.3
36166	CB544449	Bmi1	BMI1 proto-oncogene, polycomb ring finger	2,53	1,61E-09	2.53±0.28
35394	CB545570	Zranb3	zinc finger RANBP2-type containing 3	2,56	0,05	2.56±0.51
36012	D85798	Ly9	lymphocyte antigen 9	2,61	3,73E-04	2.61±0.28
35990	AI060267	Msgn1	mesogenin 1	2,62	0,01	2.62±0.36
17729	BG661061	Wfdc21	WAP four-disulfide core domain 21	2,63	1,77E-38	2.63±0.08
27542	BF567064	Epb41	erythrocyte membrane protein band 4.1	2,7	0,01	2.7±0.37
22803	BI285025	Bag1	Bcl2 associated athanogene 1	2,72	0,02	2.72±0.42
22236	CB547666	Syncrip	synaptotagmin binding, cytoplasmic RNA interacting protein	2,75	5,71E-09	2.75±0.17
36185	CA338912	Rnf126	ring finger protein 126	2,77	3,06E-07	2.77±0.2
35818	BF558909	Itih2	inter-alpha-trypsin inhibitor heavy chain 2	2,81	0,03	2.81±0.46
36014	NM_022669	Scg2	secretogranin II	2,81	0	2.81±0.32
29851	CB605696	Flt3	fms-related tyrosine kinase 3	3,09	9,96E-04	3.09±0.3
36140	CB544726	Tmem115	transmembrane protein 115	3,14	1,55E-04	3.14±0.26
35438	NM_053785	Tm4sf4	transmembrane 4 L six family member 4	3,17	0,05	3.17±0.52
36188	CB547916	Cul4b	cullin 4B	3,19	0,01	3.19±0.36
35795	BQ203909	Ankrd49	ankyrin repeat domain 49	3,45	1,80E-05	3.45±0.24
28686	BF396223	Smc2	structural maintenance of chromosomes 2	3,45	0,05	3.45±0.51
36009	CB547648	Acsl6	acyl-CoA synthetase long-chain family member 6	3,47	4,58E-14	3.47±0.13
36060	CB547226	Samhd1	SAM domain and HD domain-containing protein 1	3,59	0	3.59±0.34
34145	AA962973	Ppox	protoporphyrinogen oxidase	3,8	0,05	3.8±0.52
36013	AA925185	Stap1	signal transducing adaptor family member 1	4,05	1,99E-09	4.05±0.17
35328	CB546981	Cpvl	carboxypeptidase, vitellogenic-like	4,26	0,05	4.26±0.52
36200	NM_053333	Retnla	resistin like alpha	5,07	5,74E-04	5.07±0.29
17125	NM_053655	Dnm1l	dynamin 1 like	5,08	7,35E-04	5.08±0.3
33666	NM_017310	Sema3a	semaphorin 3A	6,26	4,15E-04	6.26±0.28
36169	NM_022530	Prl7a3	prolactin family 7, subfamily a, member 3	6,97	1,93E-05	6.97±0.24
36181	AW533710	Dcaf5	DDB1 and CUL4 associated factor 5	7,8	0	7.8±0.31

Supplementary Table S5. Differentially expressed genes in rat heart tissue following doxorubicin treatment (T3).

Sequence Code	Gene symbol	Gene description	Fold Change	P-value	Fold change±SD
BQ209483	Sgcb	sarcoglycan, beta	-5,31	0	-5.31±0.04
NM_053785	Tm4sf4	transmembrane 4 L six family member 4	-3,53	3,78E-04	-3.53±0.28
NM_012551	Egr1	early growth response 1	-2,89	5,17E-40	-2.89±0.08
AI045810	Galnt1	polypeptide N-acetylgalactosaminyltransferase 1	-2,89	0	-2.89±0.06
AW521816	Ogn	osteoglycin	-2,83	7,98E-27	-2.83±0.09
AA964911	Mb	Myoglobin	-2,77	5,49E-14	-2.77±0.13
CA507680	G0s2	G0/G1switch 2	-2,7	0	-2.7±0.06
NM_053798	Sacm1l	SAC1 like phosphatidylinositide phosphatase	-2,62	1,91E-22	-2.62±0.1
NM_022211	Fgf5	fibroblast growth factor 5	-2,58	2,60E-07	-2.58±0.19
L03557.1	Hoxa4	homeobox A4	-2,46	9,48E-05	-2.46±0.26
NM_031530	Ccl2	C-C motif chemokine ligand 2	-2,25	0	-2.25±0.07
NM_031545	Nppb	natriuretic peptide B	-2,22	0	-2.22±0.05
AI227596	Spry4	sprouty RTK signaling antagonist 4	-2,14	1,82E-21	-2.14±0.11
CB546638	Ppm1k	protein phosphatase, Mg2+/Mn2+ dependent, 1K	-2,02	0,01	-2.02±0.39
AF189709.1	Col18a1	collagen type XVIII alpha 1 chain	2,06	0,02	2.06±0.44
AW918626	Tcap	titin-cap	2,1	8,72E-11	2.1±0.15
AI175779	Neat1	nuclear paraspeckle assembly transcript 1	2,11	7,61E-05	2.11±0.25
CB546457	Atp1a1	ATPase Na+/K+ transporting subunit alpha 1	2,14	1,98E-16	2.14±0.12
AW527300	Nuak1	NUAK family kinase 1	2,15	3,64E-44	2.15±0.07
CB605612	Tmem8a	transmembrane protein 8A	2,16	3,17E-18	2.16±0.11
BG373221	Fam46b	family with sequence similarity 46, member B	2,22	2,52E-15	2.22±0.13
NM_139332	Tpcn1	two pore segment channel 1	2,25	0,01	2.25±0.4
AA925185	Stap1	signal transducing adaptor family member 1	2,26	0,02	2.26±0.42
BF558909	Itih2	inter-alpha-trypsin inhibitor heavy chain 2	2,32	0,04	2.32±0.31
D85798	Ly9	lymphocyte antigen 9	2,46	0,01	2.46±0.4
NM_031834	Sult1a1	sulfotransferase family 1A member 1	2,51	0	2.51±0.06
BG668035	Rpl34	ribosomal protein L34	2,6	0,01	2.6±0.41
AI712638	Dvl2	dishevelled segment polarity protein 2	2,62	0,01	2.62±0.37
NM_031089	Pth2r	parathyroid hormone 2 receptor	2,75	0,03	2.75±0.46
BF548548	Zbtb16	zinc finger and BTB domain containing 16	2,82	1,49E-30	2.82±0.09
CB544726	Tmem115	transmembrane protein 115	2,89	0	2.89±0.35
BQ204201	Cnksr1	connector enhancer of kinase suppressor of Ras 1	3,07	7,48E-25	3.07±0.1
AI031053	Pdk4	pyruvate dehydrogenase kinase isoenzyme 4	3,26	5,92E-32	3.26±0.09
CB547226	Samhd1	SAM domain and HD domain-containing protein 1	3,6	0,01	3.6±0.38
BF285281	Mybphl	myosin binding protein H-like	3,67	3,05E-40	3.67±0.08
AI060267	Msgn1	mesogenin 1	3,71	2,53E-06	3.71±0.21
NM_053655	Dnm1l	dynamin 1 like	3,97	0,01	3.97±0.4
AA891242	Myl7	myosin light chain 7	4,03	0	4.03±0.11
AA818120	Sln	sarcolipin	4,3	9,72E-26	4.3±0.1
AA818425	LOC100911498 (	X-inactive specific transcript	79	0	79±0.04

Supplementary Table S5. Differentially expressed genes in rat heart tissue following doxorubicin treatment (T4).

Sequence Code	Gene symbol	Gene description	Fold Change	P-value	Fold change±SD
CB544752	Phactr2	phosphatase and actin regulator 2	-9,42	4,46E-15	-9.42±0.13
AW525201	Cks2	protein kinase regulatory subunit 2	-4,68	0	-4.68±0.05
AW528001	Aspm	abnormal spindle microtubule assembly	-4,38	3,70E-20	-4.38±0.11
NM_080901	Rcvrn	recoverin	-3,91	2,98E-04	-3.91±0.28
BF388623	Cenpf	centromere protein F	-3,31	2,15E-16	-3.31±0.12
NM_031545	Nppb	natriuretic peptide B	-3,25	0	-3.25±0.04
NM_016992	Avp	arginine vasopressin	-2,96	0	-2.96±0.07
BQ210664	Cilp	cartilage intermediate layer protein	-2,81	0	-2.81±0.07
AI102920	Ube2c	ubiquitin-conjugating enzyme E2C	-2,69	4,67E-43	-2.69±0.07
AA818342	Komp	KO-first, conditional ready, lacZ-tagged mutant allele	-2,62	1,71E-19	-2.62±0.11
NM_021679	Nxph3	neurexophilin 3	-2,58	4,48E-09	-2.58±0.17
BF398599	Diaph3	diaphanous-related formin 3	-2,55	0	-2.55±0.31
NM_053356	Col1a2	collagen, type I, alpha 2	-2,54	0	-2.54±0.06
CA333722	Arcn1	archain 1	-2,52	0	-2.52±0.34
NM_012491	Add2	adducin 2	-2,49	1,39E-19	-2.49±0.11
CB548204	Bmt2	base methyltransferase of 25S rRNA 2 homolog	-2,41	7,59E-07	-2.41±0.2
BF287268	Galnt13	polypeptide N-acetylgalactosaminyltransferase 13	-2,41	2,40E-18	-2.41±0.11
BF410683	Vezf1	vascular endothelial zinc finger 1	-2,37	1,15E-24	-2.37±0.1
CB606317	Lnx2	ligand of numb-protein X 2	-2,31	0,03	-2.31±0.45
BG381632	B4galt2	beta-1,4-galactosyltransferase 2	-2,3	9,08E-11	-2.3±0.15
CB544321	Nfx1	nuclear transcription factor, X-box binding 1	-2,3	0	-2.3±0.35
S63458.1	Col8a1	collagen type VIII alpha 1 chain	-2,27	0,02	-2.27±0.42
NM_053428	Fgf13	fibroblast growth factor 13	-2,25	2,80E-45	-2.25±0.07
BQ201398	Racgap1	Rac GTPase-activating protein 1	-2,25	5,85E-31	-2.25±0.09
CA333739	Dynlt3	dynein light chain Tctex-type 3	-2,21	1,50E-13	-2.21±0.14
AI712694	Pimreg	PICALM interacting mitotic regulator	-2,18	5,21E-42	-2.18±0.07
AW531805	Ifit3	interferon-induced protein with tetratricopeptide repeats 3	-2,17	9,62E-23	-2.17±0.1
AA819788	Rtp4	receptor (chemosensory) transporter protein 4	-2,17	0	-2.17±0.06
NM_139103	Cd48	Cd48 molecule	-2,13	3,05E-12	-2.13±0.14
BQ193493	Emp1	epithelial membrane protein 1	-2,13	1,16E-37	-2.13±0.08
BQ196689	Mki67	marker of proliferation Ki-67	-2,12	0	-2.12±0.06
CB547268	Dhx38	DEAH-box helicase 38	-2,1	0	-2.1±0.34
BM389887	Hacd1	3-hydroxyacyl-CoA dehydratase 1	-2,1	0	-2.1±0.07
AW919192	Mtx2	metaxin 2	-2,1	0	-2.1±0.07
AA858882	Zfp36	zinc finger protein 36	-2,09	1,15E-27	-2.09±0.09
NM_133621	Hopx	HOP homeobox	-2,08	4,86E-39	-2.08±0.08
AI706769	Nuf2	NDC80 kinetochore complex componen	-2,08	2,67E-04	-2.08±0.27
AI237082	Hivep1	human immunodeficiency virus type I enhancer binding protein	-2,04	4,05E-09	-2.04±0.17
NM_022692	Rab5a	RAB5A, member RAS oncogene family	-2,03	0,01	-2.03±0.36
BG663818	App	amyloid beta precursor protein	-2,02	0,02	-2.02±0.43
NM_053420	Bnip3	BCL2 interacting protein 3	-2,02	0	-2.02±0.07
NM_017249	Esyt1	extended synaptotagmin 1	-2,02	0,02	-2.02±0.44
CB548192	Fbxl17	F-box and leucine-rich repeat protein 17	-2,01	1,50E-05	-2.01±0.23
CB606208	Net1	neuroepithelial cell transforming 1	-2	0,01	-2±0.37
AI227596	Spry4	sprouty RTK signaling antagonist 4	-2	2,27E-10	-2±0.16
NM_012868	Npr3	natriuretic peptide receptor 3	2	4,29E-07	2±0.15
BE127095	Thbs1	thrombospondin 1	2,03	3,83E-37	2.03±0.08
AW520335	Mcm3	minichromosome maintenance complex component 3	2,04	4,19E-21	2.04±0.11
AI502535	Zfp329	zinc finger protein 329	2,04	8,98E-09	2.04±0.17
CB544748	Gnl3l	G protein nucleolar 3 like	2,06	3,40E-10	2.06±0.16
NM_017179	Uncx	UNC homeobox	2,08	0,01	2.08±0.39
AI177265	Ahi1	Abelson helper integration site 1	2,09	1,72E-18	2.09±0.11
BE106749	Macf1	microtubule-actin crosslinking factor 1	2,11	0	2.11±0.34
BU758451	Rras2	RAS related 2	2,11	3,29E-25	2.11±0.1
BQ193605	Trappc6a	trafficking protein particle complex 6A	2,11	1,40E-20	2.11±0.11
BQ780891	Cdc23	Cell division cycle 23	2,14	1,39E-18	2.14±0.11
NM_012589	Il6	interleukin 6	2,15	0,01	2.15±0.38
BF550631	Rbm18	RNA binding motif protein 18	2,15	0,01	2.15±0.36
BE111437	Rspo3	R-spondin 3	2,16	2,54E-16	2.16±0.12
CA338912	Rnf126	ring finger protein 126	2,17	9,91E-04	2.17±0.3
CB546838	Kcnt2	potassium channel, subfamily T, member 2	2,18	0,04	2.18±0.36
CB544602	Shprh	SNF2 histone linker PHD RING helicase	2,19	1,09E-14	2.19±0.13
AI030634	Rbp7	retinol binding protein 7	2,22	5,77E-25	2.22±0.1

BF567930	Lgalsl	galectin-like	2,24	7,86E-13	2.24±0.14
NM_023974	Synpr	synaptoporin	2,24	0,02	2.24±0.42
NM_031975	Ptms	parathymosin	2,26	5,08E-15	2.26±0.13
CB546406	Frmd5	FERM domain containing 5	2,27	0,03	2.27±0.46
CB544886	Chchd6	coiled-coil-helix-coiled-coil-helix domain containing 6	2,29	0,04	2.29±0.36
NM_024402	Akap4	A-kinase anchoring protein 4	2,34	3,49E-04	2.34±0.28
BE116973	Bbs1	Bardet-Biedl syndrome 1	2,45	2,75E-11	2.45±0.15
BQ204201	Cnksr1	connector enhancer of kinase suppressor of Ras 1	2,45	3,87E-24	2.45±0.1
NM_022225	Htr1b	5-hydroxytryptamine receptor 1B	2,45	0,01	2.45±0.38
CB605954	Armc6	armadillo repeat containing 6	2,48	0	2.48±0.34
AI045144	C6	complement C6	2,48	5,68E-16	2.48±0.12
NM_012676	Tnnt2	troponin T2, cardiac type	2,55	1,55E-12	2.55±0.13
AA899832	Cox6a1	cytochrome c oxidase subunit 6A1	2,56	3,24E-04	2.56±0.28
NM_012612	Nppa	natriuretic peptide A	2,57	2,96E-18	2.57±0.11
CB545080	Taf6l	TATA-box binding protein associated factor 6 like	2,59	4,09E-06	2.59±0.22
NM_030865	Myoc	myocilin	2,63	1,40E-34	2.63±0.08
NM_023022	Rhag	Rh-associated glycoprotein	2,63	5,50E-05	2.63±0.29
NM_017239	Myh6	myosin heavy chain 6	2,69	1,72E-13	2.69±0.14
NM_012614	Npy	neuropeptide Y	2,69	0	2.69±0.05
CB546223	Fyco1	FYVE and coiled-coil domain containing 1	2,7	5,23E-12	2.7±0.14
NM_139332	Tpcn1	two pore segment channel 1	2,72	8,63E-12	2.72±0.15
BE109543	Akap7	A-kinase anchoring protein 7	2,73	0,02	2.73±0.43
U39943.1	Cyp2j3	cytochrome P450 monooxygenase 2J3	2,8	0	2.8±0.32
CB547304	Tbx6	T-box 6	2,82	7,06E-09	2.82±0.15
CB546981	Cpvl	carboxypeptidase, vitellogenic-like	2,87	1,61E-04	2.87±0.27
D85798	Ly9	lymphocyte antigen 9	2,87	2,43E-04	2.87±0.27
CB547638	Wdfy1	WD repeat and FYVE domain containing 1	2,91	0,04	2.91±0.38
BE102666	Hebp2	heme binding protein 2	2,96	0,01	2.96±0.39
NM_031615	Zfp148	zinc finger protein 148	2,98	1,98E-04	2.98±0.27
BE115196	Foxo3	forkhead box O3	3,02	2,17E-12	3.02±0.37
NM_012842	Egf	epidermal growth factor	3,06	0,01	3.06±0.38
CB546968	Dock9	dedicator of cytokinesis 9	3,08	1,25E-05	3.08±0.23
CB548004	Ppp4r3b	protein phosphatase 4, regulatory subunit 3B	3,15	1,18E-06	3.15±0.21
NM_012918	Cacna1a	calcium voltage-gated channel subunit alpha1 A	3,29	8,11E-15	3.29±0.13
CB606456	Angptl4	angiopoietin-like 4	3,34	3,68E-05	3.34±0.24
AA964911	Mb	myoglobin	3,47	1,18E-17	3.47±0.12
AI717110	Scel	sciellin	3,64	1,75E-11	3.64±0.15
BF562863	Nap1l5	nucleosome assembly protein 1-like 5	3,83	2,59E-31	3.83±0.09
CB547916	Cul4b	cullin 4B	3,87	1,96E-04	3.87±0.27
AI169104	Pf4	platelet factor 4	4,25	0	4.25±0.05
CB547998	Epm2aip1	EPM2A interacting protein 1	4,44	0,01	4.44±0.41
BF285281	Mybphl	myosin binding protein H-like	5,59	0	5.59±0.04
NM_022530	Prl7a3	prolactin family 7, subfamily a, member 3	5,65	1,80E-04	5.65±0.27
NM_053333	Retnla	resistin like alpha	6,17	3,40E-05	6.17±0.24
AA818120	Sln	sarcolipin	6,21	4,68E-36	6.21±0.08
AW533710	Dcaf5	DDB1 and CUL4 associated factor 5	6,39	0,01	6.39±0.36
NM_053785	Tm4sf4	transmembrane 4 L six family member 4	9,16	5,95E-09	9.16±0.17
AA891242	Myl7	myosin light chain 7	9,78	0	9.78±0.04

Supplementary Table S6. Inconsistently regulated DEGs among different doxorubicin treatments (T1-T4).

Gene Symbol	Gene Description	Fold Change±SD			
		T1	T2	T3	T4
**Cacna1a**	Calcium channel alpha 1A	-2.02±0.02			3.29±0.13
**Cnksr1**	Connector enhancer of kinase suppressor of Ras 1	-3.25±0.07		3.07±0.1	2.45±0.1
**Dock9**	Dedicator of cytokinesis 9		-2.41±0.43		3.08±0.23
**Mb**	Myoglobin			-2.77±0.13	3.47±0.12
**Pth**	Parathyroid hormone	3.32±0.38	-2.2±0.11		
**Rab5a**	RAB5A, member RAS oncogene family	2.57±0.36			-2.03±0.36
**Tm4sf4**	Transmembrane 4 L six family member 4		3.17±0.52	-3.53±0.28	9.16±0.17

Supplementary Table S7. Common and specific GO terms for the T1-T4 doxorubicin treatments.

Common terms_T1_up

Terms	p-value	DEGs/total number of genes specific for terms	Genes
inflammatory response	-3,177570658	10/742	C6,Camk4,Pla2g2a,Fgfr1,Mapk14,Pnma1,Gper1,Dicer1,Ccl9,Tnfaip3
response to vitamin D	-2,823889003	3/34	Pth,Penk,Gdap1
muscle structure development	-2,126863391	8/503	Neurog1,Fgfr1,Mapk14,P2rx2,Gper1,Dicer1,Dner,Etv1

Specific for T1_GeneXplain

Terms	Number of hits	Group size	p-value	Genes
cellular response to stress	11	1544	0,00747306	Ehmt2,Fabp1,Herc2,Ikbkg,Kmt2d,Nek1,Penk,Prkg2,Slf1,Tnfaip3,Usp1
organelle fission	6	378	0,001141058	Ddhd1,Ehmt2,Gdap1,Neurog1,Slf1,Sycp2
nuclear division	4	336	0,021057977	Ehmt2,Neurog1,Slf1,Sycp2
macroautophagy	3	166	0,015310002	Ehmt2,Ikbkg,Pip4k2c
mitochondrial fission	2	37	0,006018839	Ddhd1,Gdap1
positive regulation of autophagy	2	114	0,049962025	Ikbkg,Pip4k2c
autophagosome organization	2	83	0,028062512	Ehmt2,Pip4k2c
regulation of DNA repair	2	111	0,047636117	Slf1,Usp1
regulation of response to DNA damage stimulus	3	190	0,02183755	Nek1,Slf1,Usp1
meiotic cell cycle	3	198	0,02430318	Dicer1,Ehmt2,Sycp2
response to oxidative stress	3	235	0,037589766	Fabp1,Penk,Tnfaip3
striated muscle cell development	3	145	0,010654393	Dicer1,Dner,P2rx2
smooth muscle contraction	2	75	0,02324964	Neurog1,P2rx2
striated muscle cell differentiation	3	243	0,040865392	Dicer1,Dner,P2rx2
neurogenesis	11	1391	0,003397999	Cdh11,Clip1,Dicer1,Dner,Ehmt2,Etv1,Ift27,Lingo1,Neurog1,Penk,Prkch
negative regulation of programmed cell death	6	745	0,028583091	Fabp1,Ikbkg,Prkch,Pth,Sycp2,Tnfaip3
regulation of protein phosphorylation	8	1160	0,027200297	Ccl9,Dicer1,Emilin1,Ikbkg,Kmt2d,Nek1,Pla2g2a,Tnfaip3

Specific for T1_Metascape

Terms	p-value	DEGs/total number of genes specific for terms	Genes
DNA modification	0,007545823	3/101	Mtrr,Ascc3,Ehmt2
transmembrane receptor protein tyrosine kinase signaling pathway	0,002692388	8/606	Robo1,Fgfr1,Myoc,Mapk14,Pdk4,Pip4k2c,Gper1,Emilin1
neurotransmitter secretion	0,008927759	4/205	Prkce,Rab5a,P2rx2,Gper1
fatty acid oxidation	0,009528146	3/110	Fabp1,Mapk14,Pdk4
lipopolysaccharide-mediated signaling pathway	0,001425149	3/56	Prkce,Mapk14,Tnfaip3
synaptic vesicle transport	0,007516496	4/195	Prkce,Rab5a,P2rx2,Bloc1s3
IL-17 signaling pathway	0,006374067	3/95	Mapk14,Ikbkg,Tnfaip3
Fat digestion and absorption	0,000531537	3/40	Fabp1,Pla2g2a,Pnliprp2
regulation of cellular carbohydrate metabolic process	0,000619449	5/173	Pth,Slc4a1,Prkce,Pdk4,Gper1,Fabp1
olfactory bulb interneuron differentiation	7,5526E-05	3/21	Robo1,Fgfr1,Dicer1
skeletal system development	0,005837445	7/546	Pth,Neurog1,Fgfr1,Myoc,Mapk14,Dicer1,Usp1
regulation of carbohydrate metabolic process	0,001324536	5/205	Pth,Slc4a1,Prkce,Pdk4,Gper1
central nervous system neuron development	0,000774941	4/104	Robo1,Fgfr1,Cdh11,Lingo1
homeostasis of number of cells	0,00049341	7/353	Pth,Slc4a1,Slc11a2,Pla2g2a,Mapk14,Ikbkg,Tnfaip3,Prkce
response to anesthetic	0,001926058	4/133	Slc4a1,Penk,Prkce,Ehmt2
gamete generation	0,00410985	9/796	Sycp2,Nek1,Nphp1,Dicer1,Ift27,Herc2,Herc4,Ehmt2,Kmt2d
inner ear receptor cell differentiation	0,004833822	3/86	Fgfr1,Dicer1,Ift27

Supplementary Table S7. Common and specific GO terms for the T1-T4 doxorubicin treatments.

Common terms_T1_down

Terms	p-value	DEGs/total number of genes specific for terms	Genes
chromosome segregation	3,83E-06	7/272	Cenpf,Mki67,Ska1,Ube2c,Racgap1,Ect2,Cenpe
spindle organization	0,00178	4/147	Aspm,Racgap1,Cenpe,Ccnb2
mitotic cell cycle process	3,10E-04	7/543	Cenpe,Cenpf,Cks2,Ect2,Mki67,Racgap1,Ube2c
meiotic cell cycle	0,01251	3/198	Aspm,Cks2,Mki67
organelle fission	2,92E-04	6/378	Aspm,Cenpe,Cks2,Mki67,Racgap1,Ube2c
response to hypoxia	0,00612	5/431	Egr1,Nppb,Slc6a4,Ddah1,Tfrc

Specific for T1_down_GeneXplain

Terms	Number of hits	Group size	p-value	Genes
cell cycle	13	1281	6,32E-06	Aspm,Cd2ap,Cdkn3,Cenpe,Cenpf,Cks2,Ect2,Mki67,Ptpn11,Racgap1,Ska1,Slc6a4,Ube2c
mitotic cell cycle	9	703	3,94E-05	Cenpe,Cenpf,Cks2,Ect2,Mki67,Ptpn11,Racgap1,Ska1,Ube2c
protein phosphorylation	8	1502	0,02708	Cenpe,Cks2,Ect2,Mob1b,Nlrp6,Nlrp6 ,Ntrk2,Ptpn11
cell division	7	347	1,87E-05	Aspm,Cd2ap,Cks2,Ect2,Fgf5,Racgap1,Ska1
sister chromatid segregation	3	152	0,00609	Cenpe,Racgap1,Ube2c
mitotic cell cycle process	7	543	3,10E-04	
mitotic cell cycle phase transition	4	302	0,00622	Cenpe,Cenpf,Cks2,Ube2c
meiotic cell cycle	3	198	0,01251	Aspm,Cks2,Mki67
mitotic nuclear division	4	214	0,00182	Cenpe,Mki67,Racgap1,Ube2c
ERK1 and ERK2 cascade	4	284	0,00502	Nlrp6,Nlrp6 ,Ntrk2,Ptpn11
transmembrane receptor protein tyrosine kinase signaling	4	432	0,0209	Cilp,Fgf5,Ntrk2,Ptpn11
microtubule cytoskeleton organization	4	495	0,03234	Aspm,Cenpe,Racgap1,Ska1
inflammatory response	4	505	0,03444	Nlrp6,Nlrp6 ,Nppb,Ntrk2
response to growth factor	4	552	0,04537	Cilp,Fgf5,Ntrk2,Ptpn11
smooth muscle cell proliferation	2	122	0,00329	Nppb,Ogn
circadian rhythm	3	177	0,00924	Dbp,Ntrk2,Slc6a4

Specific for T1_down_Metascape

Terms	p-value	DEGs/total number of genes specific for terms	Genes
response to oxidative stress	0,00335	6/542	Reg3b,Ptpn11,Prkn,Pawr,Ect2,Kpna4
I-kappaB kinase/NF-kappaB signaling	0,0059	4/267	Prkn,Tfrc,Nlrp6,Ect2
regulation of mitotic cell cycle	0,00227	6/501	Ptpn11,Cenpf,Mki67,Ube2c,Cenpe,Cks2
nuclear division	0,00017	7/438	Aspm,Mki67,Ube2c,Racgap1,Cenpe,Ccnb2,Cks2
cell cycle phase transition	0,00624	5/433	Cenpf,Ube2c,Cenpe,Ccnb2,Cks2
mitotic sister chromatid segregation	0,00905	3/158	Ube2c,Racgap1,Cenpe
attachment of spindle microtubules to kinetochore	0,00012	3/35	Racgap1,Ect2,Cenpe
response to retinoic acid	0,00799	3/151	Slc6a4,Tfrc,Col1a2

Supplementary Table S7. Common and specific GO terms for the T1-T4 doxorubicin treatments.

Common terms_T2_Up

Terms	Number of hits	Group size	p-value	Genes
protein ubiquitination	7	627	3,24E-05	Bmi1,Cul4b,Dnm1l,Fbxo2,Hspa1b,Msl2,Rnf126
cytokine-mediated signaling pathway	4	295	3,30E-04	Flt3,Hspa1b,Samhd1,Stap1
apoptotic signaling pathway	5	613	0,008140405	Scg2,Dnm1l,Hspa1a,Tp53inp1,Bmi1
chromatin organization	9	1003	2,33E-05	Baz2a,Bmi1,Cul4b,Hspa1b2,Ldb1,Msl2,Nap1l5,Smc2,Zranb3
histone modification	5	401	0,001320534	Baz2a,Bmi1,Cul4b,Ldb1,Msl2
DNA conformation change	4	215	0,001938385	Baz2a,Zranb3,Smc2,Nap1l5
histone acetylation	3	137	0,002827592	Bmi1,Ldb1,Msl2
DNA packaging	3	147	0,004655647	Baz2a,Smc2,Nap1l5

Specific for T2_up_GeneXplain

Terms	Number of hits	Group size	p-value	Genes
cellular response to stress	7	1544	0,036234789	Cul4b,Fbxo2,Hspa1b,Hspa1b,Rnf126,Samhd1,Zranb3
immune response	8	1320	0,00112808	Bmi1,Hspa1b,Ly9,Samhd1,Serpinb1a,Stap1,Syncrip,Wfdc21
innate immune response	4	600	0,034565876	Samhd1,Serpinb1a,Syncrip,Wfdc21
adaptive immune response	2	376	0,042439483	Hspa1b,Ly9
response to endoplasmic reticulum stress	2	202	0,008331662	Fbxo2,Hspa1b
cellular response to cytokine stimulus	6	647	3,03E-04	Bmi1,Flt3,Hspa1b,Samhd1,Stap1,Syncrip
leukocyte mediated cytotoxicity	2	98	0,001083822	Hspa1b,Stap1
apoptotic mitochondrial changes	2	89	8,20E-04	Dnm1l,Hspa1b
negative regulation of apoptotic signaling pathway	2	194	0,007459152	Bmi1,Hspa1b2
chromatin organization	6	676	0,002397306	Baz2a,Bmi1,Cul4b,Ldb1,Msl2,Nap1l5
DNA metabolic process	6	738	6,70E-04	Baz2a,Bmi1,Cul4b,Hspa1b,Samhd1,Zranb3
mitotic nuclear division	2	214	0,009749335	Hspa1b,Smc2
cellular response to DNA damage stimulus	4	653	0,010530524	Cul4b,Hspa1b,Samhd1,Zranb3
mitotic cell cycle process	3	543	0,025152491	Cul4b,Hspa1b,Smc2
regulation of mitochondrion organization	2	112	0,001592393	Dnm1l,Hspa1b
defense response	6	1219	0,01118703	Hspa1b,Samhd1,Serpinb1a,Stap1,Syncrip,Wfdc21
ATP metabolic process	2	252	0,015126864	Dnm1l,Hspa1b
DNA repair	4	397	0,00126308	Cul4b,Hspa1b,Samhd1,Zranb3
protein metabolic process	18	4868	0,003576004	Baz2a,Bmi1,Cpvl,Cul4b,Dnm1l,Fbxo2,Flt3,Hspa1b,Itih2,LOC108348108,Ldb1,Msl2,Ppp1r3b,Rnf126, Serpinb1a,Stap1,Syncrip,Wfdc21
erythrocyte differentiation	2	101	0,001182519	Hspa1b,Ldb1
T cell activation	3	377	0,042720556	Bmi1,Flt3,Ly9
response to glucocorticoid	2	172	0,04937098	Bmi1,Flt3

Specific for T2_up_Metascape

Terms	p-value	DEGs/total number of genes specific for terms	Genes
purine nucleotide metabolic process	0,003931678	5/515	Pdk4,Dnm1l,Acsl6,Hspa1a,Samhd1
DNA conformation change	0,00193838	4/259	Baz2a,Zranb3,Smc2,Nap1l5
Protein processing in endoplasmic reticulum	0,004735154	3/165	Fbxo2,Hspa1a,Bag1
regulation of chemotaxis	0,001422558	4/238	Scg2,Sema3a,Dnm1l,Stap1
protein monoubiquitination	0,000494137	3/75	Cul4b,Bmi1,Rnf126
regulation of protein ubiquitination	0,000994294	4/216	Fbxo2,Dnm1l,Hspa1a,Bmi1
protein modification by small protein conjugation	0,001049607	7/793	Fbxo2,Dnm1l,Hspa1a,Cul4b,Bmi1,Rnf126,Msl2
regionalization	0,007575797	4/380	Sema3a,Bmi1,Ldb1,Msgn1
protein acetylation	0,009316622	3/211	Bmi1,Ldb1,Msl2
regulation of response to cytokine stimulus	0,002165948	3/125	Hspa1a,Stap1,Samhd1
purine-containing compound metabolic process	0,005723632	5/563	Pdk4,Dnm1l,Acsl6,Hspa1a,Samhd1
regulation of protein modification by small protein conjugation or removal	0,00172871	4/251	Fbxo2,Dnm1l,Hspa1a,Bmi1
regulation of apoptotic signaling pathway	0,001732103	5/426	Scg2,Dnm1l,Hspa1a,Tp53inp1,Bmi1

Supplementary Table S7. Common and specific GO terms for the T1-T4 doxorubicin treatments.

Common terms_T2_down

Terms	p-value	DEGs/total number of genes specific for terms	Genes
smooth muscle cell proliferation	0,001004428	3/186	Adamts1,Itga2,Ogn
glucose metabolic process	0,002324374	3/170	Fbn1,Pth,Src
smooth muscle cell migration	0,004509314	3/104	Itga2,Src
transmembrane receptor protein serine/threonine kinase signaling pathway	0,005850663	4/266	Fbn1,Src,Col1a2,Cilp

Specific for T2_down_GeneXplain

Terms	Number of hits	Group size	p-value	Genes
phosphorus metabolic process	11	2512	7,72E-04	Cilp,Cinp,Dab1,Fbn1,Ikbke,Itga2,Nudt13,Pou1f1,Pth,Snrk,Src
cell differentiation	11	3205	0,00588223	Aspm,Col6a2,Col8a1,Dab1,Fbn1,Itga2,Nav1,Pou1f1,Prdm1,Pth,Src
immune system process	7	2157	0,04384505	Fbn1,Ikbke,Itga2,Hoxa3,Pou1f1,Prdm1,Src
leukocyte differentiation	4	421	0,00394585	Fbn1,Pou1f1,Prdm1,Src
response to growth factor	4	552	0,01018486	Cilp,Fbn1,Pth,Src
organophosphate metabolic process	4	702	0,02277958	Nudt13,Pou1f1,Pth,Src
hemopoiesis	4	707	0,02331317	Fbn1,Pou1f1,Prdm1,Src
insulin-like growth factor receptor signaling pathway	2	28	8,75E-04	Cilp,Pou1f1
myeloid cell development	2	66	0,00478865	Fbn1,Src
B cell differentiation	2	93	0,00930625	Pou1f1,Prdm1
cellular response to lipopolysaccharide	2	191	0,03594637	Prdm1,Src
system development	13	3853	0,00248902	Aspm,Col6a2,Col8a1,Dab1,Fbn1,Glrb,Itga2,Hoxa3,Nav1,Pou1f1,Prdm1,Pth,Src
multicellular organism development	13	4207	0,00569915	Aspm,Col6a2,Col8a1,Dab1,Fbn1,Glrb,Itga2,Hoxa3,Nav1,Pou1f1,Prdm1,Pth,Src
anatomical structure development	13	4635	0,01359726	Aspm,Col6a2,Col8a1,Dab1,Fbn1,Glrb,Itga2,Hoxa3,Nav1,Pou1f1,Prdm1,Pth,Src
developmental process	13	5057	0,02842211	Aspm,Col6a2,Col8a1,Dab1,Fbn1,Glrb,Itga2,Hoxa3,Nav1,Pou1f1,Prdm1,Pth,Src

Specific for T2_down_Metascape

Terms	p-value	DEGs/total number of genes specific for terms	Genes
Extracellular matrix organization	3,21549E-06	6/219	Fbn1,Col1a2,Col5a1,Itga2,Col8a1,Col6a2
blood vessel development	0,002261406	6/727	Ptgis,Adamts1,Col1a2,Col5a1,Prdm1,Hoxa3
transforming growth factor beta receptor signaling pathway	0,005233633	3/186	Fbn1,Src,Col1a2
integrin-mediated signaling pathway	0,001309217	3/114	Adamts1,Src,Itga2
extracellular matrix organization	0,002639733	4/307	Adamts1,Col1a2,Col5a1,Col8a1
collagen metabolic process	0,001181158	3/110	Col1a2,Col5a1,Itga2
cardiovascular system development	0,00289875	6/764	Ptgis,Adamts1,Col1a2,Col5a1,Prdm1,Hoxa3
PI3K-Akt signaling pathway	0,003649267	4/336	Col1a2,Itga2,Col6a2,Col6a3
Platelet activation	0,001825182	3/128	Src,Col1a2,Itga2
positive regulation of cellular carbohydrate metabolic process	0,000357154	3/73	Pth,Pou1f1,Src,Col1a2,Dock9,Dab1,Fbn1,Hoxa3
muscle cell migration	0,001445712	3/118	Adamts1,Src,Itga2
response to acid chemical	0,000795995	6/593	Reg3b,Glrb,Adamts1,Src,Col1a2,Itga2
skeletal system development	0,003500902	5/546	Pth,Fbn1,Src,Col1a2,Hoxa3
formation of primary germ layer	0,001825182	3/128	Col5a1,Itga2,Col8a1
neuron migration	0,005715048	3/192	Dab1,Aspm,Nav1
vasculature development	0,00289875	6/764	Ptgis,Adamts1,Col1a2,Col5a1,Prdm1,Hoxa3

Supplementary Table S7. Common and specific GO terms for the T1-T4 doxorubicin treatments.

Common terms_T3_Up

Terms	Number of hits	Group size	p-value	Genes
heart contraction	4	233	0,002937654	Atp1a1,Dnm1l,Tcap,Sult1a1

Specific for T3_up_GeneXplain

Terms	Number of hits	Group size	p-value	Genes
response to stress	4	1043	0,017756686	Dvl2,Nuak1,Samhd1,Stap1
positive regulation of apoptotic process	3	495	0,012420632	Col18a1,Dnm1l,Zbtb16
immune effector process	3	678	0,028562031	Ly9,Samhd1,Stap1
leukocyte activation	3	781	0,040997267	Ly9,Stap1,Zbtb16
regulation of cytokine-mediated signaling pathway	2	83	0,003081983	Samhd1,Stap1
myeloid leukocyte migration	2	156	0,010491395	Dnm1l,Stap1
T cell differentiation	2	205	0,017632922	Ly9,Zbtb16
cytokine-mediated signaling pathway	2	295	0,034698911	Samhd1,Stap1
calcium ion transport	2	320	0,040250129	Dnm1l,Sln
steroid metabolic process	2	195	0,01604402	Atp1a1,Sult1a1
multicellular organismal process	12	6901	0,010658925	Atp1a1,Col18a1,Dnm1l,Dvl2,Ly9,Msgn1,Mybphl,Samhd1,Sln,Stap1,Sult1a1,Zbtb16
developmental process	11	5057	0,002651036	Col18a1,Dnm1l,Dvl2,Ly9,Msgn1,Mybphl,Nuak1,Samhd1,Stap1,Tmem115,Zbtb16
multicellular organism development	9	4207	0,011055234	Col18a1,Dnm1l,Dvl2,Ly9,Msgn1,Mybphl,Samhd1,Stap1,Zbtb16
anatomical structure development	9	4635	0,021245926	Col18a1,Dnm1l,Dvl2,Ly9,Msgn1,Mybphl,Samhd1,Stap1,Zbtb16
positive regulation of biological process	9	5022	0,03568875	Atp1a1,Col18a1,Dnm1l,Dvl2,Ly9,Msgn1,Sln,Stap1,Zbtb16
cellular developmental process	8	3260	0,008166879	Col18a1,Dnm1l,Dvl2,Ly9,Nuak1,Stap1,Tmem115,Zbtb16
regulation of multicellular organismal process	7	2541	0,007954874	Atp1a1,Dnm1l,Dvl2,Ly9,Sln,Stap1,Zbtb16
positive regulation of multicellular organismal process	6	1499	0,002426838	Atp1a1,Dnm1l,Dvl2,Ly9,Sln,Zbtb16
regulation of catalytic activity	6	1768	0,005599569	Dnm1l,Dvl2,Itih2,Nuak1,Sln,Stap1

Specific for T3_up_Metascape

Terms	p-value	DEGs/total number of genes specific for terms	Genes
purine nucleotide metabolic process	0,003508867	4/515	Sult1a1,Pdk4,Dnm1l,Samhd1
circulatory system process	0,003657515	4/521	Atp1a1,Sult1a1,Dnm1l,Tcap
heart process	0,003268981	3/242	Atp1a1,Dnm1l,Tcap
nucleoside phosphate metabolic process	0,007602733	4/641	Sult1a1,Pdk4,Dnm1l,Samhd1
blood circulation	0,003388206	4/510	Atp1a1,Sult1a1,Dnm1l,Tcap
nucleotide metabolic process	0,007277531	4/633	Sult1a1,Pdk4,Dnm1l,Samhd1
anterior/posterior pattern specification	0,002937654	3/233	Zbtb16,Tcap,Msgn1
cellular response to extracellular stimulus	0,00736301	3/324	Pdk4,Dnm1l,Nuak1
cellular response to nutrient levels	0,005318422	3/288	Pdk4,Dnm1l,Nuak1
cellular response to external stimulus	0,00203543	4/443	Atp1a1,Pdk4,Dnm1l,Nuak1
purine-containing compound metabolic process	0,004820732	4/563	Sult1a1,Pdk4,Dnm1l,Samhd1

Supplementary Table S7. Common and specific GO terms for the T1-T4 doxorubicin treatments.

Common terms_T3_down

Terms	Number of hits	Group size	p-value	Genes
response to fibroblast growth factor	3	89	4,66E-05	Ccl2,Fgf5,Spry4
smooth muscle cell proliferation	3	186	3,40E-04	Egr1,Nppb,Ogn
response to hypoxia	4	431	0,000245905	Egr1,Ccl2,Nppb,Mb
wound healing	3	499	0,005761896	Egr1,Ccl2,Tm4sf4
cellular response to fibroblast growth factor stimulus	3	120	9,32279E-05	Ccl2,Fgf5,Spry4
regulation of smooth muscle cell proliferation	3	223	0,000303861	Egr1,Nppb,Ogn
muscle cell proliferation	3	253	0,000834027	Egr1,Nppb,Ogn

Specific for T3_down_GeneXplain

Terms	Number of hits	Group size	p-value	Genes
phosphorus metabolic process	5	2512	0,041714587	Ccl2,Nppb,Ppm1k,Sacm1l,Spry4
response to fibroblast growth factor	3	89	4,66E-05	Ccl2,Fgf5,Spry4
smooth muscle cell proliferation	2	122	1,19E-04	Nppb,Ogn
response to hypoxia	3	279	0,001342499	Ccl2,Mb,Nppb
cardiac muscle cell development	2	68	0,00136412	Nppb,Sgcb
ERK1 and ERK2 cascade	2	284	0,021785813	Ccl2,Spry4
wound healing	2	309	0,025502713	Ccl2,Tm4sf4
regulation of cell population proliferation	5	1317	0,002832438	Ccl2,Fgf5,NEWGENE_1308171,Nppb,Ogn
response to endogenous stimulus	5	1409	0,003812975	Ccl2,Fgf5,Mb,Nppb,Spry4
cell population proliferation	5	1576	0,006200796	Ccl2,Fgf5,NEWGENE_1308171,Nppb,Ogn
cell surface receptor signaling pathway	5	2058	0,018961632	Ccl2,Fgf5,G0s2,Nppb,Spry4
phosphate-containing compound metabolic process	5	2495	0,040640721	Ccl2,Nppb,Ppm1k,Sacm1l,Spry4
negative regulation of cell population proliferation	4	527	6,72E-04	Ccl2,NEWGENE_1308171,Nppb,Ogn
enzyme linked receptor protein signaling pathway	4	679	0,001731898	Ccl2,Fgf5,Nppb,Spry4
circulatory system development	4	872	0,004315353	Ccl2,Mb,Nppb,Sgcb
homeostatic process	4	1471	0,026441359	Ccl2,G0s2,Mb,Nppb
negative regulation of smooth muscle cell proliferation	3	39	3,84E-06	NEWGENE_1308171,Nppb,Ogn
cellular response to fibroblast growth factor stimulus	3	83	3,78E-05	Ccl2,Fgf5,Spry4
regulation of smooth muscle cell proliferation	3	118	1,08E-04	NEWGENE_1308171,Nppb,Ogn
muscle cell proliferation	3	168	3,07E-04	NEWGENE_1308171,Nppb,Ogn

Specific for T3_down_Metascape

Terms	p-value	DEGs/total number of genes specific for terms	Genes
cellular response to fibroblast growth factor stimulus	9,32279E-05	3/120	Ccl2,Fgf5,Spry4
response to hypoxia	0,000245905	4/431	Egr1,Ccl2,Nppb,Mb
smooth muscle cell proliferation	0,000340068	3/186	Egr1,Nppb,Ogn
wound healing	0,005761896	3/499	Egr1,Ccl2,Tm4sf4
response to fibroblast growth factor	0,000112917	3/128	Ccl2,Fgf5,Spry4
cellular response to growth factor stimulus	0,002170152	4/770	Egr1,Ccl2,Fgf5,Spry4
gliogenesis	0,002620076	3/377	Egr1,Ccl2,Fgf5
transmembrane receptor protein tyrosine kinase signaling pathway	0,009844186	3/606	Ccl2,Fgf5,Spry4
regulation of smooth muscle cell proliferation	0,000303861	3/179	Egr1,Nppb,Ogn
response to decreased oxygen levels	0,000325881	4/464	Egr1,Ccl2,Nppb,Mb
response to oxygen levels	0,000539723	4/530	Egr1,Ccl2,Nppb,Mb
muscle cell proliferation	0,000834027	3/253	Egr1,Nppb,Ogn
response to mechanical stimulus	0,001952464	3/340	Egr1,Ccl2,Nppb
cellular response to external stimulus	0,004131487	3/443	Egr1,Ccl2,Nppb
striated muscle tissue development	0,004344217	3/451	Egr1,Nppb,Sgcb
muscle tissue development	0,005051977	3/476	Egr1,Nppb,Sgcb
response to peptide hormone	0,00898052	3/586	Egr1,Ccl2,Nppb
response to antibiotic	0,009406429	3/596	Egr1,Ccl2,Mb

Supplementary Table S7. Common and specific GO terms for the T1-T4 doxorubicin treatments.

Common terms_T4_Up

Terms	Number of hits	Group size	p-value	Genes
smooth muscle cell proliferation	4	122	3,86E-04	Htr1b,Il6,Npr3,Thbs1
striated muscle contraction	4	176	0,003469	Nppa,Tnnt2,Myh6,Mb
ERBB signaling pathway	4	117	0,000777	Nppa,Egf,Myoc,Rnf126
Wnt signaling pathway	7	429	0,000771	Nppa,Egf,Myoc,Foxo3,Scel,Macf1,Rspo3
epidermal growth factor receptor signaling pathway	5	304	0,004368	Il6,Mb,Foxo3,Hebp2,Thbs1
	3	107	0,006417	Nppa,Egf,Rnf126

Specific for T4_up_GeneXplain

Terms	Number of hits	Group size	p-value	Genes
response to stress	15	3033	0,006889	Angptl4,Chchd6,Cul4b,Foxo3,Hebp2,Il6,Mb,Mcm3,Nppa,Npy,Pf4,Rnf126,Shprh,Thbs1,Tm4sf4
response to oxidative stress	5	372	0,00387	Foxo3,Hebp2,Il6,Mb,Thbs1
response to hypoxia	4	279	0,007865	Angptl4,Foxo3,Mb,Nppa
cell death	8	1573	0,029948	Angptl4,Foxo3,Hebp2,Il6Npy,Pf4,Rras2,Thbs1
extrinsic apoptotic signaling pathway	3	183	0,014943	Foxo3,Pf4,Thbs1
endothelial cell apoptotic process	3	47	3,15E-04	Angptl4,Foxo3,Thbs1
organelle organization	14	3142	0,047507	Bbs1,Cdc23,Chchd6,Cul4b,Frmd5,Fyco1,Gnl3l,Hebp2,Mcm3,Nap1l5,Shprh,Taf6l,Tnnt2,Trappc6a
chromosome organization	7	1003	0,021618	Cdc23,Cul4b,Gnl3l,Mcm3,Nap1l5,Shprh,Taf6l
chromatin assembly	2	113	0,040662	Nap1l5,Shprh
positive regulation of mitotic cell cycle	2	126	0,049454	Cdc23,Cul4b
protein ubiquitination	5	627	0,031292	Cdc23,Cul4b,Gnl3l,Rnf126,Shprh
DNA conformation change	3	215	0,022824	Mcm3,Nap1l5,Shprh
cellular response to DNA damage stimulus	5	653	0,036352	Chchd6,Cul4b,Foxo3,Mcm3,Shprh
cellular response to glucocorticoid stimulus	2	69	0,0163	Foxo3,Il6
inflammatory response	5	505	0,002838	Il6,Nppa,Npy,Pf4,Thbs1
MAPK cascade	4	614	0,028941	Il6,Nppa,Npy,Thbs1
response to tumor necrosis factor	3	185	0,015381	Foxo3,Il6,Thbs1
T-helper 17 cell differentiation	2	24	0,002079	Il6,Ly9
erythrocyte differentiation	2	101	0,033146	Foxo3,Mb
myeloid leukocyte activation	3	186	0,001859	Nppa,Npy,Thbs1
granulocyte migration	2	106	0,036205	Pf4,Thbs1
granulocyte chemotaxis	2	87	0,025172	Pf4,Thbs1
humoral immune response	2	292	0,049437	Npy,Pf4
heart development	5	492	0,012272	Mb,Nppa,Tbx6,Thbs1,Tnnt2
cardiac muscle contraction	2	89	0,026256	Nppa,Tnnt2
gluconeogenesis	2	63	0,013715	Il6,Ppp4r3b
glucose metabolic process	3	170	0,012272	Epm2aip1,Il6,Ppp4r3b
mitochondrial membrane organization	2	96	0,030197	Chchd6,Hebp2
positive regulation of cytokine biosynthetic process	2	54	0,010212	Il6,Thbs1
cellular response to tumor necrosis factor	3	167	0,011698	Foxo3,Il6,Thbs1
leukocyte activation involved in immune response	3	212	0,022006	Il6,Ly9,Nppa
wound healing	4	309	0,011154	Il6,Pf4,Thbs1,Tm4sf4
angiogenesis	4	364	0,01926	Angptl4,Pf4,Rspo3,Thbs1
transmembrane receptor protein tyrosine kinase signaling pathway	4	432	0,033374	Foxo3,Nppa,Rnf126,Thbs1
G protein-coupled receptor signaling pathway	3	186	0,015603	Htr1b,Npr3,Pf4

Specific for T4_up_Metascape

Terms	p-value	DEGs/total number of genes specific for terms	Genes
regulation of acute inflammatory response	0,002276	3/74	C6,Il6,Npy
muscle hypertrophy	0,007825	3/115	Nppa,Myh6,Myoc
regulation of transmembrane transport	0,00539	7/606	Nppa,Cacna1a,Tpcn1,Ahi1,Akap7,Sln,Thbs1
regulation of membrane potential	0,006925	6/481	Il6,Nppa,Cacna1a,Myoc,Hebp2,Akap7
regulation of epithelial cell apoptotic process	0,005173	3/99	Foxo3,Angptl4,Thbs1
HIF-1 signaling pathway	0,006417	3/107	Il6,Nppa,Egf
Cardiac muscle contraction	0,002944	3/81	Tnnt2,Cox6a1,Myh6
striated muscle adaptation	0,000966	3/55	Nppa,Myh6,Myoc
striated muscle hypertrophy	0,007457	3/113	Nppa,Myh6,Myoc
ammonium transport	0,00136	4/136	Nppa,Htr1b,Cacna1a,Rhag
myeloid cell homeostasis	0,004991	4/195	Il6,Mb,Rhag,Foxo3
heart morphogenesis	0,002895	5/276	Tnnt2,Myh6,Ahi1,Tbx6,Thbs1
muscle system process	0,000825	7/434	Nppa,Tnnt2,Myh6,Mb,Myoc,Foxo3,Sln
regulation of carbohydrate metabolic process	0,005948	4/205	Il6,Egf,Epm2aip1,Ppp4r3b

Supplementary Table S7. Common and specific GO terms for the T1-T4 doxorubicin treatments.

Common terms_T4_down

Terms	Number of hits	Group size	p-value	Genes
organelle fission	7	378	1,54E-05	Aspm,Bnip3,Cks2,Mki67,Nuf2,Racgap1,Ube2c
nuclear division	6	336	8,25E-05	Aspm,Cks2,Mki67,Nuf2,Racgap1,Ube2c
chromosome segregation	5	272	3,01E-04	Cenpf,Mki67,Nuf2,Racgap1,Ube2c
mitotic cell cycle phase transition	5	302	4,87E-04	App,Cenpf,Cks2,Hacd1,Ube2c
meiotic cell cycle	4	198	9,04E-04	Aspm,Cks2,Mki67,Nuf2
G2/M transition of mitotic cell cycle	3	95	0,001178	App,Cenpf,Hacd1
sister chromatid segregation	3	126	0,00264415	Nuf2,Racgap1,Ube2c
spindle organization	3	147	0,00408262	Aspm,Nuf2,Racgap1

Specific for T4_down_GeneXplain

Terms	Number of hits	Group size	p-value	Genes
cell cycle	10	1281	3,15E-04	App,Aspm,Cenpf,Cks2,Dynlt3,Hacd1,Mki67,Nuf2,Racgap1,Ube2c
mitotic cell cycle	9	703	1,54E-05	App,Cenpf,Cks2,Dynlt3,Hacd1,Mki67,Nuf2,Racgap1,Ube2c
chromosome organization	6	1003	0,0209178	App,Hopx,Mki67,Nuf2,Racgap1,Ube2c
microtubule cytoskeleton organization	4	495	0,02267557	Aspm,Fgf13,Nuf2,Racgap1
MAPK cascade	4	614	0,04478643	App,Avp,Fgf13,Spry4
cellular response to oxidative stress	3	235	0,01473641	App,Bnip3,Net1
ERK1 and ERK2 cascade	3	284	0,02423387	App,Avp,Spry4
epithelial cell proliferation	3	332	0,03610886	Col8a1,Mki67,Nppb
calcium ion homeostasis	3	366	0,04604261	App,Avp,Bnip3
release of cytochrome c from mitochondria	2	45	0,00439322	Avp,Bnip3
apoptotic mitochondrial changes	2	89	0,01635852	Avp,Bnip3
spindle assembly	2	91	0,01705948	Aspm,Racgap1
fatty acid biosynthetic process	2	107	0,02311363	Avp,Hacd1
heart contraction	2	157	0,04662719	Fgf13,Hopx
cellular response to growth factor stimulus	4	528	0,02791724	App,Cilp,Hivep1,Spry4
muscle cell differentiation	3	303	0,02863041	Hacd1,Hopx,Nppb
cellular calcium ion homeostasis	3	357	0,04329219	App,Avp,Bnip3
response to oxidative stress	3	372	0,04792399	App,Bnip3,Net1
chromosome localization	2	66	0,00924761	Cenpf,Nuf2
mitotic spindle organization	2	93	0,01777312	Nuf2,Racgap1
regulation of lipid biosynthetic process	2	120	0,0285891	App,Avp
regulation of mitotic nuclear division	2	126	0,03127524	Mki67,Ube2c
cellular response to reactive oxygen species	2	145	0,04040377	Bnip3,Net1

Specific for T4_down_Metascape

Terms	p-value	fic for terms	Genes
mitotic sister chromatid segregation	0,005568771	3/158	Ube2c,Nuf2,Racgap1
cell cycle phase transition	0,002919902	5/433	App,Cenpf,Ube2c,Cks2,Hacd1
organelle fission	0,000110324	7/491	Bnip3,Aspm,Mki67,Ube2c,Nuf2,Racgap1,Cks2
mitotic cell cycle process	0,000155638	8/699	App,Cenpf,Mki67,Ube2c,Nuf2,Racgap1,Cks2,Hacd1
Signaling by Rho GTPases	0,008051577	4/349	Diaph3,Nuf2,Net1,Racgap1
circulatory system process	0,00638237	5/521	Avp,Nppb,App,Col1a2,Hopx
regulation of mitotic cell cycle	0,000125037	7/501	App,Cenpf,Mki67,Ube2c,Dynlt3,Cks2,Hacd1
neuroblast proliferation	0,000566133	3/71	Fgf13,Aspm,Racgap1
regulation of G2/M transition of mitotic cell cycle	0,001129208	3/90	App,Cenpf,Hacd1
regulation of cell cycle process	0,00272207	6/623	App,Cenpf,Mki67,Ube2c,Racgap1,Hacd1
regulation of cell-substrate adhesion	0,001888025	4/232	Spry4,Col8a1,Net1,Hacd1
positive regulation of cell-substrate adhesion	0,004136696	3/142	Col8a1,Net1,Hacd1
mitotic cell cycle phase transition	0,001834349	5/389	App,Cenpf,Ube2c,Cks2,Hacd1
cell cycle G2/M phase transition	0,00482043	3/150	App,Cenpf,Hacd1

Supplementary Table S8. Independent confirmation of p53/Abl1 target genes.

A. Independent validation of p53/Abl1 target genes shown in Figure 6D

gene name	ChIP-seq signal - human p53 BAER-	FISHER human p53-dependent regulation	FISHER mouse p53-dependent regulation	FISHER Occupancy TSS - enhancers	FISHER DREAM target
	~44 datasets	57 datasets	15 datasets		
**UP**					
ROBO1	NO	DW in 9 UP in 5	DW in 1 UP in 2	No - No	No
IGSF21	NO	UP in 3	DW in 2	No - No	No
BLOC1S3	NO	UP in 11	UP in 2	No - No	No
CCL15 -CCL9	NO	no data	no data	no data	no data
AIF1L	NO	DW in 18	UP in 4	No - No	No
ZNF746	NO	UP in 24	DW in 3	No - Yes	No
**DW**					
UHRF1	NO	DW >50%	DW >50%	No - No	YES
NLRP6	YES	UP in 2	UP in 1	No - No	No
NIT2	NO	DW in 5	UP in 3	No - No	No
NPPB	NO	UP in 3		No - No	No
CNKSR1	NO	UP in 5		No - No	No
PIMREG	NO	DW >50%	DW >50%	No - No	YES
UBE2C	NO	DW >50%	DW >50%	No - No	YES

B. Independent validation of p53 target genes shown in Figure 6E&F

UP

Stap1		No changes	No changes	No - No	No
Dnm1L		DW in 8, UP in 1	DW in 1	No - No	No
Bmi1		DW in 22	DW in 2	No - No	No
Ppox		DW in 5, UP in 1	No changes	No - No	No
Epb41		Dw in 9, UP in 5	DW in 4	No - No	No
Zramb3		No info	No info	No info	No info
Itih2		UP in 2	DW in 1, UP in 1	No - No	No
Pp1					
Baz2a		DW in5, UP in 3	No info	No - No	No
Hspa1a		DW in 15, UP in 5	No info	No - No	No
Sema3a		DW in 15, UP in 3	DW in 5, UP in 1	No - No	No
Nap115					
Tp53inp1		UP in 57	UP in 14	Yes-Yes	Yes
Ankrd49		DW in 2, UP in 4	DW in 3, UP in 1	No - No	No
Cpv1		UP in 6	UP in 1	No - No	No
Bag1		DW in 3, UP in 19	UP in 1	No - Yes	No
Pp1r3b					
Mos					
Prl7a3					
Rnfl26					

Tmem115		DW in 1, UP in 8	UP in 3	No - No	No
Serpinb1a		DW in 5, UP in 14	No data	No - No	No
Flt3		DW in 4, UP in 3	UP in 1	No - No	No
Tm4sf4		UP in 5	No changes	No - No	No
Acs16					
Scg2		DW in 4, UP in 12	UP in 1	No - No	No
Smc2		DW in 42, UP in 1	DW in 7	No - No	Yes
Fbxo2		UP in 31	DW in 1, UP in 4	No - No	No

DW

Cilp		UP in 5	No changes	No - Yes	No
Rps4y2					
Nav1		DW in 11, UP in 1	DW in 3	No - No	No
Fbn1		DW in 7, UP in 15	DW in 3	No - No	No
Aspm		DW in 51	DW in 8	No - No	Yes
Cul6a2					
Hoxa3		DW in 9, UP in 3	DW in 2	No - No	No
Nudt13		DW in 3	UP in 1	No - No	No
Adamts1		DW in 18, UP in 6	DW in 5	No - No	No
Reg3b					
Ogn		UP in 2	DW in 7	No - No	No
Cinp		DW in 7, UP in 1	No changes	No - No	No
Pon1f1					
Col8a1		DW in 9, UP in 11	DW in 3	No - No	No
Dock9		DW in 7, UP in 6	DW in 2, UP in 6	No - No	No
Slc5a11					
Pop5		DW in 6, UP in 2	UP in 2	No - No	No
Col5a1		DW in 7, UP in 25	DW in 3	No - No	No
Col1a2		DW in 4, UP in 6	DW in 3, UP in 1	No - No	No
Ubxn11		DW in 10, UP in 3	UP in 2	Yes- No	No
Dab1		DW in 6, UP in 5	DW in 2, UP in 1	No - No	No
Smrk					
Tcx35					
Src		UP in 22	UP in 1	Yes- No	No
Ikbkc					
Itga2		DW in 5, UP in 30	UP in 4	No - Yes	No
Pth					
Ust4r					
Glrb		DW in 4, UP in 8	DW in 2, UP in 1	No - No	No
Kcna6		DW in 2, UP in 1	UP in 6	No - No	No

Supplementary Table S8. Independent confirmation of p53/Abl1 target genes.

C. Independent validation of p53 target genes shown in Figure 6A-C.

	ChIP-seq signal - human p53 BAER-	FISHER human p53-dependent regulation	FISHER mouse p53-dependent regulation	FISHER Occupancy TSS - enhancers	FISHER DREAM target		FISHER human DNp63-dependent regulation
	~44 datasets	57 datasets	15 datasets				15 datasets
TPCN1	few/low signal	UP in 18	DW in 3	no - YES	no		DW in 4 UP in 3
HOPX	NO	DW in 3 UP in 2	DW in 3 UP in 1	no - no	no		DW in 3 UP in 2
PLA2G2A	NO	UP in 4 DW in 1	no change	no - no	no		no data
SYCP2	NO	DW in 12 UP in 2	UP in 2	no - no	no		DW in 1
DNM1L	NO	DW in 8 UP in 1	DW in 1	no - no	no		UP in 10 DW in 2

Supplementary Table S8. Independent confirmation of p53/Abl1 target genes.

D. Independent validation of p53 target genes shown in Figure 6A-C.

**Chromosome Name**	5	5	4	2	16
**Gene Start (bp)**	120984169	77515468	138387772	178079998	16312328
**Gene End (bp)**	121038622	77544186	138391101	178142390	16359123
**Strand**	-1	-1	1	-1	-1
**Ensembl Gene ID**	**ENSMUSG00000032741**	**ENSMUSG00000059325**	**ENSMUSG00000058908**	**ENSMUSG00000060445**	**ENSMUSG00000022789**
**Associated Gene Name**	**Tpcn1**	**Hopx**	**Pla2g2a**	**Sycp2**	**Dnm1l**
**p53 Expression Score mouse**	-3	-2	0	2	-1
**Tonelli 2015 IR non-B-cells log2FC**	-0,919452093	0,09484784	---	1,194573557	-0,088986676
**Tonelli 2015 IR non-B-cells adj.p-value**	6,37285E-08	0,701830045	---	0,01460946	0,593796623
**Tonelli 2015 IR non-B-cells (-1,0,1)**	-1	0	0	1	0
**Tonelli 2015 IR B-cells log2FC**	-0,754615213	-1,320404703	0,311077353	0,169174553	0,047705197
**Tonelli 2015 IR B-cells adj.p-value**	1,99397E-05	2,25908E-08	NA	0,786601568	0,61277442
**Tonelli 2015 IR B-cells (-1,0,1)**	-1	-1	0	0	0
**Younger 2015 doxorubicin MEF log2FC**	---	---	---	---	---
**Younger 2015 doxorubicin MEF q-value**	---	---	---	---	---
**Younger 2015 doxorubicin MEF (-1,0,1)**	0	0	0	0	0
**Dimitrova 2014 doxorubicin MEF log2FC**	-0,553763468	1,333741068	---	1,195452031	-0,504122833
**Dimitrova 2014 doxorubicin MEF adj.p-value**	2,31E-03	5,34E-03	---	1,02E-02	4,39E-03
**Dimitrova 2014 doxorubicin MEF (-1,0,1)**	-1	1	0	1	-1
**Kenzelmann-Broz 2013 doxorubicin MEF log2FC**	---	---	---	---	---

**Kenzelmann-Broz 2013 doxorubicin MEF adj.p-value**	---	---	---	---	---
**Kenzelmann-Broz 2013 doxorubicin MEF (-1,0,1)**	0	0	0	0	0
**Marín-Béjar 2013 siTP53 MEF log2FC**	-0,023	0,341	0,131	0,0108	-0,0496
**Marín-Béjar 2013 siTP53 MEF adj.p-value**	0,824536	0,035897	0,232555	0,943683	0,54294
**Marín-Béjar 2013 siTP53 MEF (-1,0,1)**	0	0	0	0	0
**Gambino 2013 doxorubicin MEF log2FC**	0,035025	0,2411775	0,132035	0,02895	0,047375
**Gambino 2013 doxorubicin MEF p-value**	0,737	0,45	0,243	0,732	0,615
**Gambino 2013 doxorubicin MEF adj.p-value**	0,800179	0,549674	0,341819	0,796164	0,697579
**Gambino 2013 doxorubicin MEF (-1,0,1)**	0	0	0	0	0
**Zhang 2013 doxorubicin MEF log2FC**	---	---	---	---	---
**Zhang 2013 doxorubicin MEF p-value**	---	---	---	---	---
**Zhang 2013 doxorubicin MEF (-1,0,1)**	0	0	0	0	0
**Brady 2011 p53 knock in MEF log2FC**	0,01662215	-1,15147074	0,01045934	0,01290174	-0,14120944
**Brady 2011 p53 knock in MEF adj.p-value**	0,989	0,0282	0,976	0,976	0,424
**Brady 2011 p53 knock in MEF (-1,0,1)**	0	-1	0	0	0
**Huarte 2010 siTP53 #1 MEF log2FC**	0,35978977	0,13606664	-0,06922917	-0,01508793	-0,36098661
**Huarte 2010 siTP53 #1 MEF adj.p-value**	0,2209536	0,595898	0,8296447	0,9725705	0,652233
**Huarte 2010 siTP53 #1 MEF (-1,0,1)**	0	0	0	0	0

**Huarte 2010 siTP53 #2 MEF log2FC**	0,11868594	0,67421808	0,29798947	0,20571518	0,06501008
**Huarte 2010 siTP53 #2 MEF adj.p-value**	0,5854019	0,0127943	0,1809451	0,2778161	0,7715132
**Huarte 2010 siTP53 #2 MEF (-1,0,1)**	0	-1	0	0	0
**Huarte 2010 doxorubicin MEF log2FC**	-0,167	-0,531	-0,148	-0,000409	-0,324
**Huarte 2010 doxorubicin MEF adj.p-value**	0,62351752	0,05398051	0,63309993	0,99930319	0,11504214
**Huarte 2010 doxorubicin MEF (-1,0,1)**	0	0	0	0	0
**Huarte 2010 KRAS MEF log2FC**	0,08981245	0,87169716	0,00103882	0,08633245	0,10290782
**Huarte 2010 KRAS MEF adj.p-value**	0,5628235	0,6991238	0,99802632	0,6382988	0,81041296
**Huarte 2010 KRAS MEF (-1,0,1)**	0	0	0	0	0
**Lee 2010 doxorubicin mESC log2FC**	0,16822575	-0,3911515	0,06952675	0,128979	-0,31194025
**Lee 2010 doxorubicin mESC adj.p-value**	0,0399	0,000437	0,472	0,091	0,000181
**Lee 2010 doxorubicin mESC (-1,0,1)**	0	0	0	0	0
